# Highly Selective Carbonylation of Olefins Using CO_2_ and H_2_

**DOI:** 10.1021/jacs.5c09325

**Published:** 2025-08-28

**Authors:** Mohamed Niyaz Vellala Syed Ali, Weiheng Huang, Xinxin Tian, Haijun Jiao, Ralf Jackstell, Robert Franke, Matthias Beller

**Affiliations:** 1 Leibniz Institute for Catalysis, Albert-Einstein-Straße 29a, 18059 Rostock, Germany; 2 Institute of Molecular Science, Shanxi University, Taiyuan 030006, China; 3 Lehrstuhl für Theoretische Chemie, 44780 Bochum, Germany; 4 Evonik Oxeno GmbH, Paul-Baumann-Str. 1, 45772 Marl, Germany

## Abstract

A unique multimetallic catalyst system that allows for the direct carbonylation of aliphatic and aromatic olefins with carbon dioxide and hydrogen has been developed. The employment of palladium and iridium precursors, in conjunction with 1,2-bis­(di-*tert*-butylphosphino­methyl)­benzene (d^*t*^bpx) or its derivatives as active ligands and Zn­(OTf)_2_ (OTf: trifluoromethane­sulfonate) as an essential acidic additive, has been shown to result in the selective formation of aliphatic esters in CO_2_-based carbonylations, exhibiting unparalleled regioselectivity. Detailed control experiments and mechanistic investigations suggest an initial Ir-catalyzed formation of alkyl formates from carbon dioxide, followed by Pd-catalyzed alkoxycarbonylation at a low CO concentration. The desired transformation has been observed to occur in several industrially relevant alkene substrates, thereby creating a potential avenue for further research and industrial applications.

## Graphical abstract

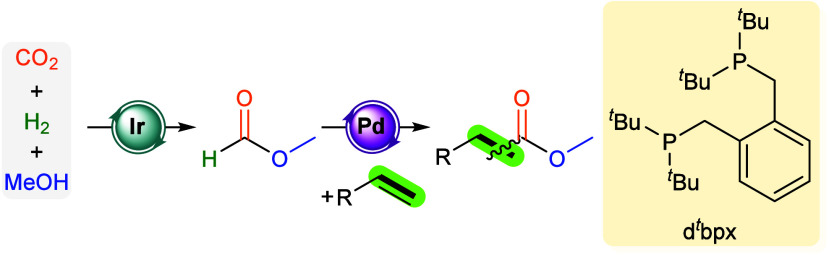

## Introduction

Carbon monoxide (CO) is a pivotal C1 chemical feedstock for the chemical industry, which is applied to produce essential bulk chemicals, e.g. oxo products,^1^ acetic acid,^2^ as well as many advanced specialties. Transformations such as carbonylation of olefins, alkynes, alcohols, and organic halides are the cornerstones of modern catalysis. In addition, combination of CO and hydrogen allows to create C–C bonds in the presence of suitable catalysts and thereby provides the possibility to obtain diverse alkanes, alkenes, and alcohols in a highly efficient manner (Fischer–Tropsch process).^3^ Consequently, industrially relevant carbonylations with CO have been a pillar of industrial chemistry in the 20th century and will be for the coming decades, too.

More specifically, hydroformylation, traditionally termed oxo synthesis, combines synthesis gas (mixtures of CO and H_2_) with olefins, resulting in aldehydes. This singular methodology yields more than 10 million metric tons of oxo chemicals annually.^4^ A comparable process of significant importance is the carbonylation of methanol, which is catalyzed by either a Rh catalyst (Monsanto process) or an Ir catalyst (Cativa process).^5^ This process has the capacity to yield more than 18 million tons per year of acetic acid. Concurrently, the Lucite-Alpha process, which is utilized in the production of methyl methacrylate (MMA), the monomer of poly­(methyl methacrylate) (PMMA), has an annual output exceeding 370,000 tons. The announcement of an additional plant employing this technology has been made (Figure 1a).^6^

**Figure 1:**
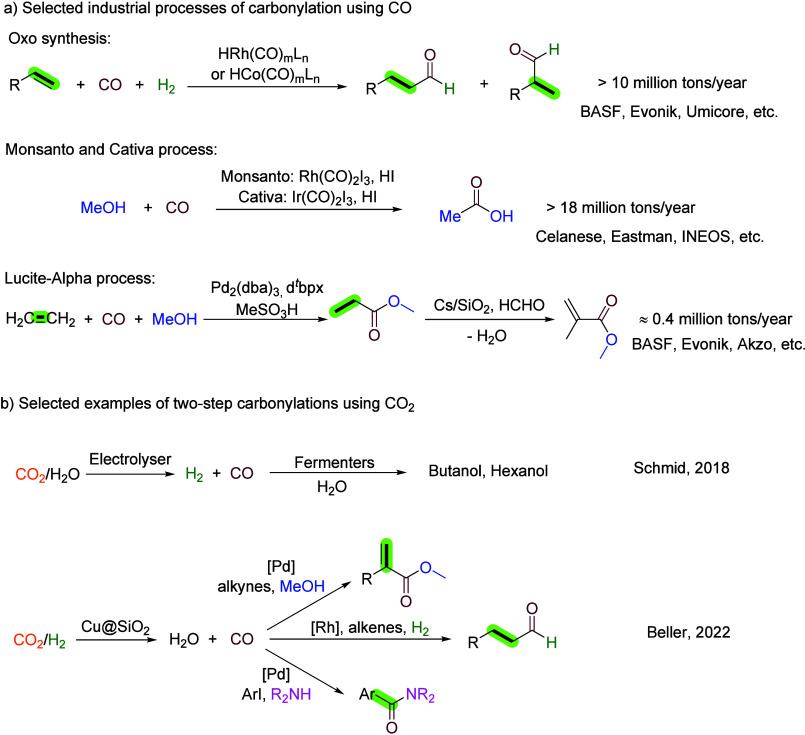
Selected industrial carbonylation processes and two-step carbonylations using CO_2_.

It is important to note that the vast majority of CO utilized in the manufacturing of the aforementioned products is produced through conventional high-temperature steam reforming processes that depend on fossil resources, specifically gas, coal, and crude oil.^7^ Consequently, the production and current utilization of CO in carbonylations are accountable for a substantial amount of carbon dioxide formation in the chemical industry. To address this problem, several strategies have been devised applying nonfossil-based CO in recent years (Figure 1b). One such approach involves the conversion of (bio)­waste or CO_2_ in an upstream reaction step to CO, which after undergoing a purification process, can be utilized. For instance, our research group has developed a cascade process that employs a distinct heterogeneous copper catalyst to generate CO from the hydrogenation of CO_2_. The resulting synthesis gas is then utilized for carbonylations of olefins.^8^ In a joint research initiative named “Rheticus” Siemens Energy and Evonik converted CO_2_ and H_2_O to syngas via electrochemical reduction, which was subsequently employed in fermentation to generate sustainable chemicals like butanol.^9,10^

While these concepts contribute to the reduction of the CO_2_ footprint in the respective production processes, they still have the disadvantage of producing vast quantities of highly toxic CO and necessitate an additional step for the subsequent utilization of CO. A more streamlined solution would be the *in situ* conversion of CO_2_ to CO and its direct valorization in carbonylation reactions, e.g., of olefins. In this hypothetical scenario, green hydrogen would the most practical and appropriate reductant; however, this transformation possesses several challenges: First, CO_2_ must be selectively hydrogenated in the presence of olefins. Second, the generated CO deactivates typical hydrogenation catalysts. Third, apart from the chemoselectivity, the regioselectivity of the reaction must be controlled, which is only possible at “low” reaction temperatures (<150 °C). Consequently, there is a paucity of reports on the direct carbonylation of alkenes using CO_2_ (Figure 2a).

**Figure 2:**
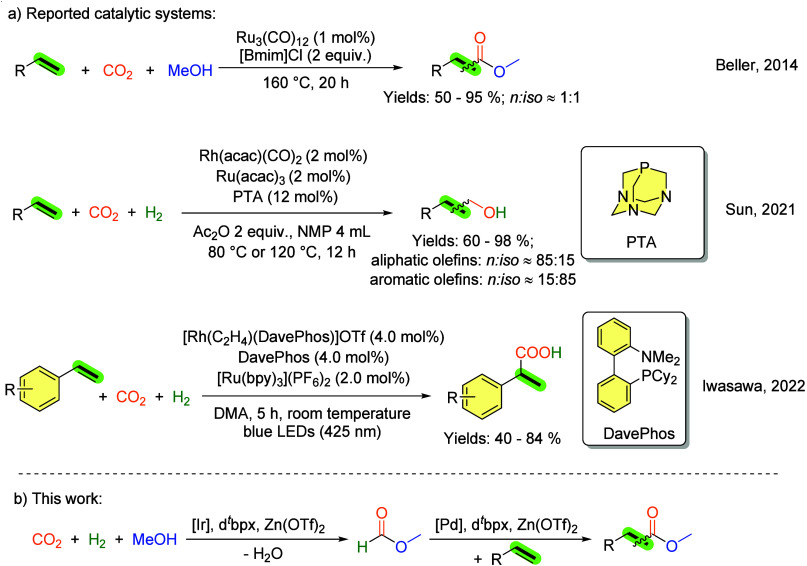
Reported catalytic systems and this work for direct carbonylation of olefins using H_2_/CO_2_.

As an example, approximately a decade ago, our group presented a catalytic system employing Ru_3_CO_12_ with a substantial amount of 1-butyl-3-methylimidazolium chloride ([Bmim]­Cl).^11^ In this reaction, methanol functioned not only as a solvent but also as a reducing agent, allowing subsequent alkoxycarbonylation of olefins. However, the application of terminal olefins invariably yielded mixtures of regioisomers (n:iso approximately 1:1). Subsequent studies have outlined various examples of alkoxycarbonylation of olefins with CO_2_, utilizing analogous catalytic systems. However, the prevailing selectivity issue remains unresolved.^12–14^ In a recent development, Sun et al. have devised a sophisticated Rh–Ru dual catalyst system for domino hydroformylation/reduction of olefins with CO_2_ and H_2_ in the presence of 2 equiv of acetic anhydride (Ac_2_O).^15,16^ In this process, both rhodium and ruthenium are ligated with 1,3,5-triaza-7-phosphaadamantane (PTA). The authors propose an initial reduction of CO_2_ to formic acid, which reacts with Ac_2_O to produce CO instantaneously. Mechanistic control experiments demonstrated that the hydroformylation of alkenes is catalyzed by the Rh-PTA catalyst and that the corresponding aldehydes were selectively reduced to the corresponding alcohols by the Ru-PTA catalyst. In 2022, Iwasawa and co-workers demonstrated a photocatalytic hydrocarboxylation of styrene using CO_2_ and H_2_ in the presence of a rhodium complex and a ruthenium photocatalyst.^17^ According to the proposed mechanism, the photocatalyst accelerates the formation of an active rhodium hydride complex under visible light.

Following our previous work on carbonylations utilizing CO surrogates such as alkyl formates^18,19^ and formic acid,^20^ we became attracted to design a multimetallic catalyst system for domino CO_2_ reduction–alkoxycarbonylation processes. Rather than the direct valorization of methyl formate in carbonylation reactions, we recognized that methyl formate generated in situ from carbon dioxide more plausibly serves as a convenient intermediate for the controlled release of carbon monoxide, which is then utilized in the subsequent carbonylation step (Figure 2b). To the best of our knowledge, such selective transformations have not been previously described.

In this study, we present the first instance of palladium–iridium catalyzed alkoxycarbonylations of olefins with CO_2_/H_2_ and alcohols (Table 1). Notably, the corresponding ester products are obtained with excellent selectivity for the linear isomer. Crucial for the success of this transformation is the combination of two specific components: the use of a specific (commercially available) ligand 1,2-bis­(di-*tert*-butylphosphino­methyl)­benzene (d^*t*^bpx) (Lucite ligand), in conjunction with Zn­(OTf)_2_ as a Lewis acid additive.

**Table 1:**
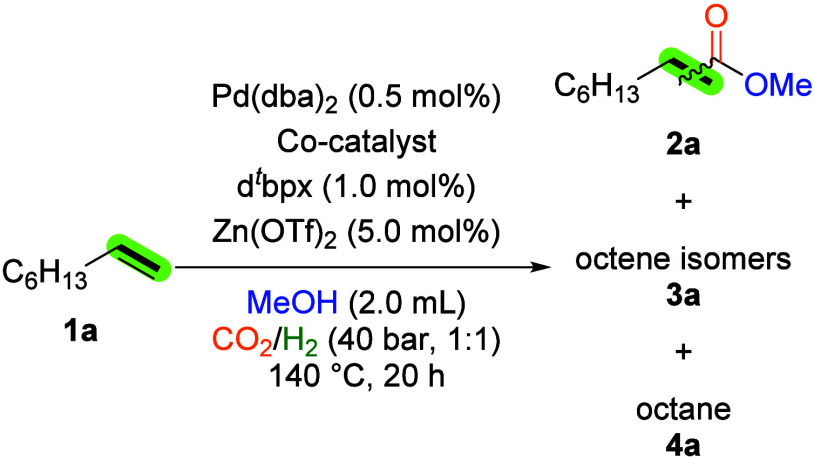
Alkoxycarbonylation of 1-Octene with Methanol, CO_2_ and H_2_ in the Presence of Selected Bimetallic Catalyst Systems^a^

**Entry**	**Co-catalyst**	**Yield of 2a**	***n*** **:** ***i***	**Yield of 3a and 4a**
1	–	<1%	–	98% (major **3a**)
2	Fe_3_(CO)_12_	<1%	–	97% (major **4a**)
3	Ru_3_(CO)_12_	8%	92:8	90% (major **4a**)
4	Co_2_(CO)_8_	12%	92:8	87% (major **3a**)
5	Ir(acac)(CO)_2_	25%	92:8	74% (major **3a**)

a Reaction conditions: **1a** (1.0 mmol), Pd­(dba)_2_ (0.5 mol %), Co-catalyst ([M] = 0.05 mol %), d^*t*^bpx (1.0 mol %), Zn­(OTf)_2_ (5.0 mol %), MeOH (2 mL), CO_2_ (20 bar), H_2_ (20 bar), 140 °C, 20 h reaction time. The ratio of linear to all branched products = *n:i*. Yields and *n:i* are determined by GC analysis using isooctane (57 mg, 0.5 mmol) as internal standard.

## Results and Discussion

Our initial investigations of potential catalyst systems and suitable reaction conditions were conducted using 1-octene (**1a**) as the model substrate. It is noteworthy that this reaction system serves as a particularly challenging benchmark reaction for such transformations primarily due to the propensity of 1-octene for isomerization reactions. Indeed, none of the known syntheses of esters and acids from olefins and carbon dioxide provide high regioselectivity for aliphatic and aromatic olefins.

Initially, the model reaction was conducted under various conditions exclusively with palladium catalysts, which are recognized as state-of-the-art metals for alkoxycarbonylations with carbon monoxide. However, this approach did not yield a significant amount of esters, as evidenced in Table 1, entry 1. Instead, mainly unwanted olefin isomerization took place. This finding indicated that the reduction of CO_2_ and the carbonylation steps are not effectively promoted by a single active catalyst center. At this point, the importance of a second metal catalyst became evident, prompting an expansion of the investigation by incorporating additional metals from Groups 8 (Fe, Ru) and 9 (Co, Ir) as cocatalysts. However, Group 8 metals, specifically Fe exhibited no activity, while Ru demonstrated marginal activity (Table 1, entries 2 and 3). Subsequently, Group 9 metals were examined, with Co yielding a notable amount of the desired esters and Ir yielding the highest yield among the metals used (Table 1, entries 4 and 5). Based on these observations, we selected Pd–Ir as the bimetallic system and initiated further optimization studies.

As shown in Table 2 and Table S1, the effect of more than 25 ligands was thoroughly examined. While the majority of the ligands displayed no reactivity, a 25% yield of methyl nonanoates (**2a**) was obtained in the presence of Pd­(dba)_2_ (dba: dibenzylideneacetone), Ir­(acac)­(CO)_2_, d^*t*^bpx ligand and Zn­(OTf)_2_ additive in a methanol solution under 40 bar of CO_2_ and H_2_ (CO_2_:H_2_ = 1:1) at 140 °C for a 20 h reaction time (Table 2, entry 1).

**Table 2:**
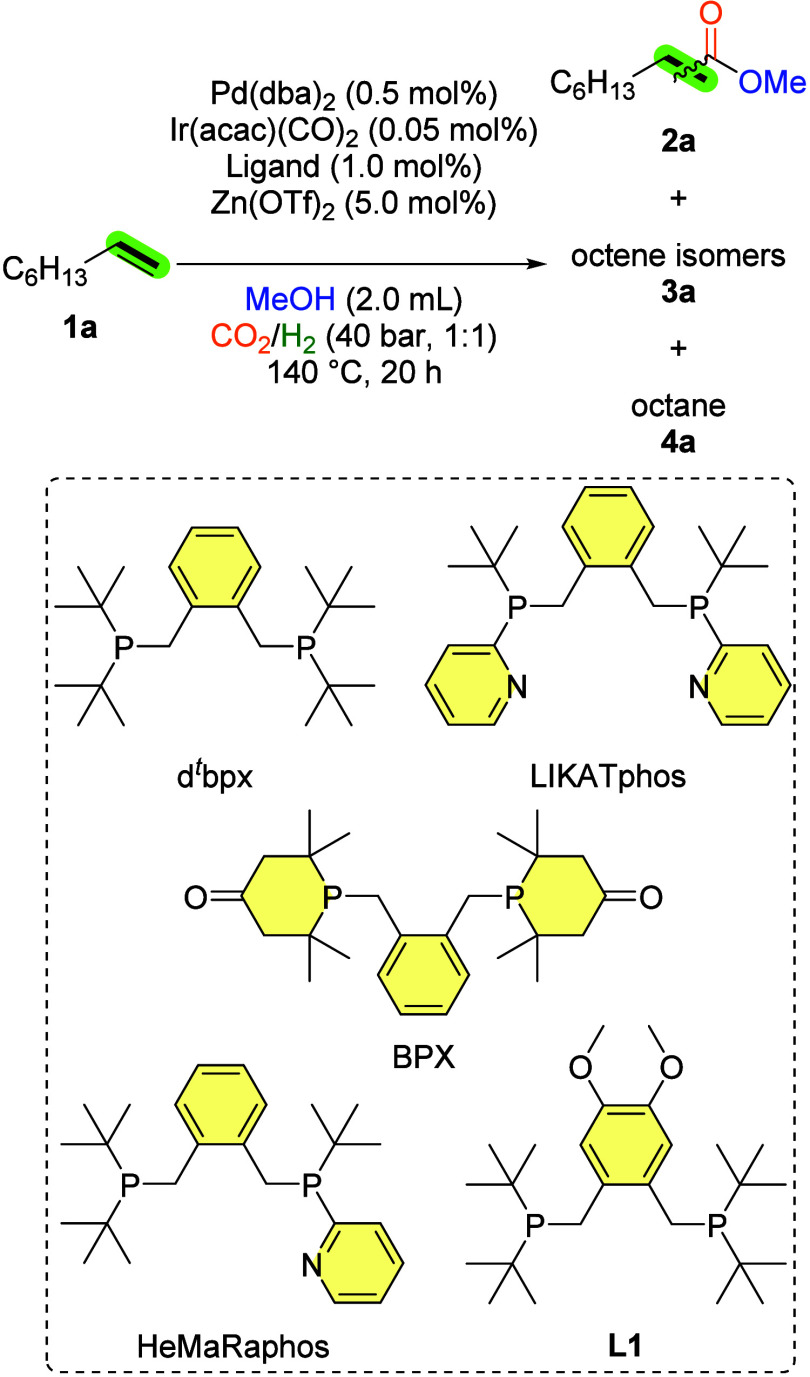
Palladium–Iridium Catalyzed Alkoxycarbonylation of 1-Octene with Methanol and CO_2_/H_2_: Selected Ligand Investigations^a^

**Entry**	**Ligand**	**Yield of 2a**	***n*** **:** ***i***	**Yield of 3a and 4a**
1	d^*t*^bpx	25%	92:8	74% (major **3a**)
2	BPX	8%	95:5	90% (major **3a**)
3	LIKATphos	<1%	–	98% (major **3a**)
4	HeMaRaphos	2%	–	97% (major **3a**)
5	L1	33%	92:8	65% (major **3a**)

a Reaction conditions: **1a** (1.0 mmol), Pd­(dba)_2_ (0.5 mol %), Ir­(acac)­(CO)_2_ (0.05 mol %), ligand (1.0 mol %), Zn­(OTf)_2_ (5.0 mol %), MeOH (2 mL), CO_2_ (20 bar), H_2_ (20 bar), 140 °C, 20 h reaction time. The ratio of linear to all branched products = *n:i*. Yields and *n:i* are determined by GC analysis using isooctane (57 mg, 0.5 mmol) as internal standard.

Perhaps the most surprising outcome of our research was the excellent linear-to-branched ratio (92:8), which has never before been observed in hydrogenative carbonylations with carbon dioxide. Next, structurally related ligands were applied in the reaction. The application of 1,2-bis­(4-phosphorinone)­xylene (BPX) ligand^21^ resulted in a lower yield of **2a** (8%, Table 2, entry 2). Intriguingly, only a small amount of esters are observed using LIKATphos^22–24^ and HeMaRaphos,^25^ both of which are extremely efficient ligands using CO for related carbonylations (Table 2, entries 3 and 4). Finally, an electron-rich derivative of d^*t*^bpx, **L1**, was synthesized and tested, demonstrating improved reactivity, and providing a 33% yield of **2a** (Table 2, entry 5). Preliminary conclusions drawn from the results of Table 2 suggest that the *tert*-butyl groups attached to the phosphine appear to be crucial for the active catalytic species. Furthermore, the presence of more electron-rich catalytic species corresponds to higher reactivity.

The initial step in palladium–iridium catalyzed alkoycarbonylations of olefins is the formation of an active Pd–H and Ir–H species. In general, the presence of acids such as *p*-toluenesulfonic acid (PTSA) is necessary for this step. Consequently, we assumed that the palladium–iridium catalyzed reduction of CO_2_ would be influenced significantly by such additives. Hence, a comprehensive investigation was undertaken, as detailed in Tables 3 and S2. However, when PTSA was utilized, the hydrogenation of 1-octene emerged as the predominant reaction (>90%), with no ester being detected (Table 3, entry 1). In contrast, when other Lewis acidic triflates, such as Al­(OTf)_3_ and Fe­(OTf)_3_, were employed, octane (**4a**) emerged as the predominant product (>80%). Furthermore, the presence of octene isomers was detected (Table 3, entries 2 and 3). However, testing zinc halide salts proved unsuccessful due to their limited solubility in methanol solutions. In this case, the predominant product was octene isomers (Table 3, entry 4). The use of Zn­(OAc)_2_ resulted in a homogeneous solution, but no carbonylation occurred, likely due to the reduced Lewis acidity (Table 3, entry 5). However, the application of zinc sulfonate additives resulted in the formation of the desired esters (**2a**). A comparison of Zn­(OTf)_2_ as the benchmark reveals that zinc *p*-toluenesulfonate provided a lower yield of **2a** (14%, Table 3, entry 6), while the presence of zinc methansulfonate resulted in a comparable yield of **2a** (23%, Table 3, entry 7). Furthermore, when the amount of Zn­(OTf)_2_ was reduced to 2.0 mol %, the yield of **2a** decreased to 12% (Table 3, entry 8). Conversely, the yield of **2a** increased when 10 mol % of Zn­(OTf)_2_ was applied; however, the yield of ester did not continue to increase with additional concentration of Zn­(OTf)_2_ additive (Table 3, entries 9 and 10).

**Table 3:**
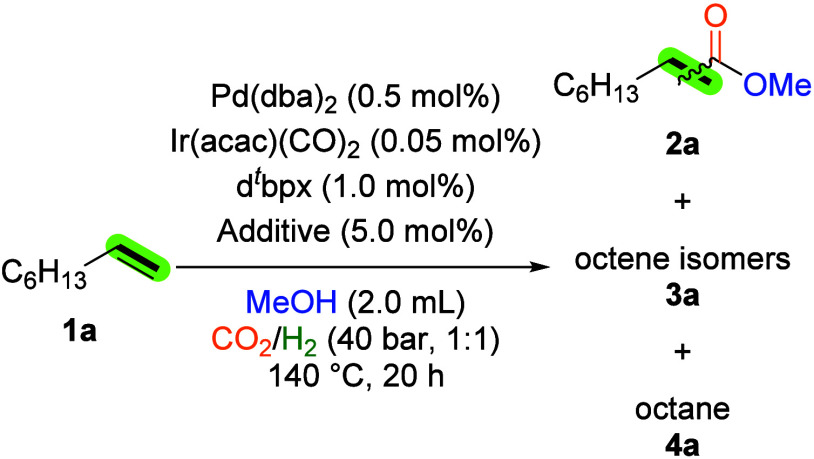
Palladium–Iridium Catalyzed Alkoxycarbonylation of 1-Octene with Methanol and CO_2_/H_2_: Effect of Selected Additives^a^

**Entry**	**Additives**	**Yield of 2a**	***n*** **:** ***i***	**Yield of 3a and 4a**
1	PTSA·H_2_O	–	–	98% (major **4a**)
2	Al(OTf)_3_	–	–	98% (major **4a**)
3	Fe(OTf)_3_	–	–	98% (major **4a**)
4^b^	ZnX_2_	–	–	98% (major **3a**)
5	Zn(OAc)_2_	–	–	98% (major **3a**)
6	Zn(SO_3_C_6_H_4_CH_3_)·xH_2_O	14%	92:8	85% (major **3a**)
7	Zn(SO_3_CH_3_)·xH_2_O	23%	94:6	76% (major **3a**)
8^c^	Zn(OTf)_2_	12%	91:9	87% (major **3a**)
9^d^	Zn(OTf)_2_	30%	91:9	68% (major **3a**)
10^e^	Zn(OTf)_2_	30%	91:9	69% (major **3a**)

a Reaction conditions: **1a** (1.0 mmol), Pd­(dba)_2_ (0.5 mol %), Ir­(acac)­(CO)_2_ (0.05 mol %), d^*t*^bpx (1.0 mol %), additives (5.0 mol %), MeOH (2 mL), CO_2_ (20 bar), H_2_ (20 bar), 140 °C, 20 h reaction time. The ratio of linear to all branched products = *n:i*. Yields and *n:i* are determined by GC analysis using isooctane (57 mg, 0.5 mmol) as internal standard.

b X = F, Cl, Br, I.

c Zn­(OTf)_2_ (2.0 mol %).

d Zn­(OTf)_2_ (10.0 mol %).

e Zn­(OTf)_2_ (20.0 mol %).

To optimize reaction conditions for this multimetallic catalyst system, various parameters were investigated, including pressure, temperature, CO_2_/H_2_ ratio and cosolvents were investigated, too (see Supporting Information, Tables S3 to S5). Remarkably, the desired transformation performed similarly well when the reaction temperature was decreased to 120 °C.

To further enhance the ester yield, we tested different palladium and iridium precursors (Supporting Information, Table S6). Notably, the hydrogenation reaction dominated, and the yield of **2a** decreased when applying halide precursors like [Ir­(cod)­Cl]_2_, IrCl_3_, Pd­(cod)­Cl_2_, and PdBr_2_. To rule out the influence of Pd^2+^ versus Pd^0^ and Ir^0^ versus Ir^1+^, Pd­(OAc)_2_ and Ir_4_(CO)_12_ were tested, yielding outcomes analogous to those observed with Pd­(dba)_2_ and Ir­(acac)­(CO)_2_.

It is noteworthy that the optimized catalyst system demonstrated a remarkable stability. It was determined that extending the reaction time and increasing the catalyst loading led to a notable increase in the yield of **2a** (Figure 3, Table S6). Applying a catalyst loading of 2 mol % of Pd­(dba)_2_, 0.2 mol % of Ir­(acac)­(CO)_2_ and 4 mol % of d^*t*^bpx gave a 66% yield of **2a** after 20 h; extending the reaction time (45 h) resulted in a 74% yield of **2a**. Using a higher catalyst loading (3 mol % Pd­(dba)_2_/0.3 mol % Ir­(acac)­(CO)_2_/6 mol % d^*t*^bpx) for 45 h the yield of **2a** reached 77%, accompanied by a 20% yield of octane. Under these latter conditions, which allowed for full conversion of all octenes, ligand **L1** was tested and resulted in an 83% yield of **2a**. When the Pd­(OAc)_2_ and Ir­(acac)­(CO)_2_ precursors were tested in conjunction with d^*t*^bpx and **L1** under these conditions, comparable yields of 80% and 84% were obtained.

**Figure 3:**
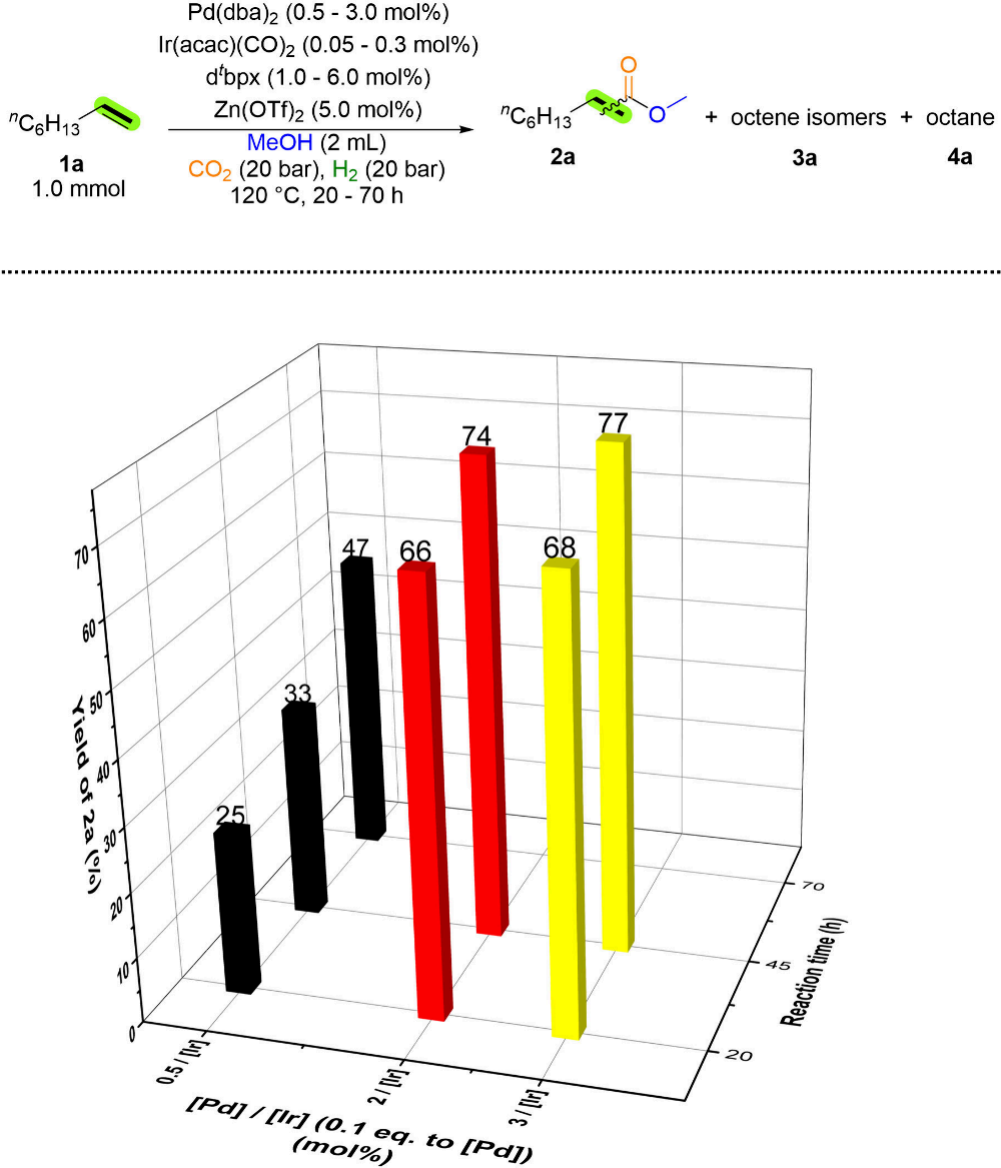
Investigations of catalyst productivity and stability. Reaction conditions: 1-octene (1.0 mmol), Ir­(acac)­(CO)_2_, Pd­(dba)_2_ (10 equiv. to Ir­(acac)­(CO)_2_), d^*t*^bpx (2 equiv. to Pd­(dba)_2_), Zn­(OTf)_2_ (5.0 mol %), MeOH (2 mL), CO_2_ (20 bar), H_2_ (20 bar), 120 °C. Yields are determined by GC analysis using isooctane (57 mg, 0.5 mmol) as internal standard.

After the optimization of the reaction conditions, the investigation of the reaction pathway and the underlying mechanism of this transformation was initiated. Initially, we hypothesized that in the presence of our catalyst system, carbon dioxide and hydrogen undergo a reverse water gas shift reaction (RWGS) generating CO. However, heterogeneous catalysts are predominantly used for this transformation at (much) higher temperatures (>200 °C).^26^ Analysis of the gas phase of catalytic reactions under optimized conditions after 6 and 20 h revealed around 0.2% of free CO in the gas phase (see Supporting Information, Figures S1 to S4). This observation is consistent with the results of an experiment conducted without an olefin. Notably, in the absence of the substrate 1-octene and palladium, a new product, methyl formate, can be detected and quantified precisely by ^1^H NMR spectroscopy without the interruption from olefins and ester products. A decomposition test of methyl formate using stochiometric amounts of water under N_2_ atmosphere showed the formation of small amounts of CO (1.3%), as well as H_2_ and CO_2_. This indicates that CO is generated from methyl formate, and the hydrogenation of CO_2_ is reversible (see Supporting Information, Figure S5). Consequently, we hypothesized that iridium would reduce CO_2_ to its corresponding alkyl formate, which would then undergo alkoxycarbonylation in olefins by palladium. In order to identify alkyl formates as intermediates in the aforementioned protocol, a series of control experiments and kinetic investigations were conducted using n-butanol as the solution (see Supporting Information, Figures S9 to S18). The kinetic profiles of the carbonylation reactions of two alkenes, 1-octene (Figure 4) and cyclohexene (Supporting Information, Figure S16), were measured. As depicted in Figure 4, the corresponding butyl formate is obtained exclusively by employing the Ir catalyst in the presence of CO_2_/H_2_ (black line). In a separate experiment, the addition of 1-octene to the same reaction system resulted in the formation of butyl formate, accompanied by octene isomers (major) and *n*-octane (minor). This finding underscores the notion that the role of the active iridium species is merely that of a reduction catalyst, which is incapable of further carbonylating alkenes. The presence of butyl formate (BF) is detected in the absence (black line) and presence (red line) of 1-octene.

**Figure 4:**
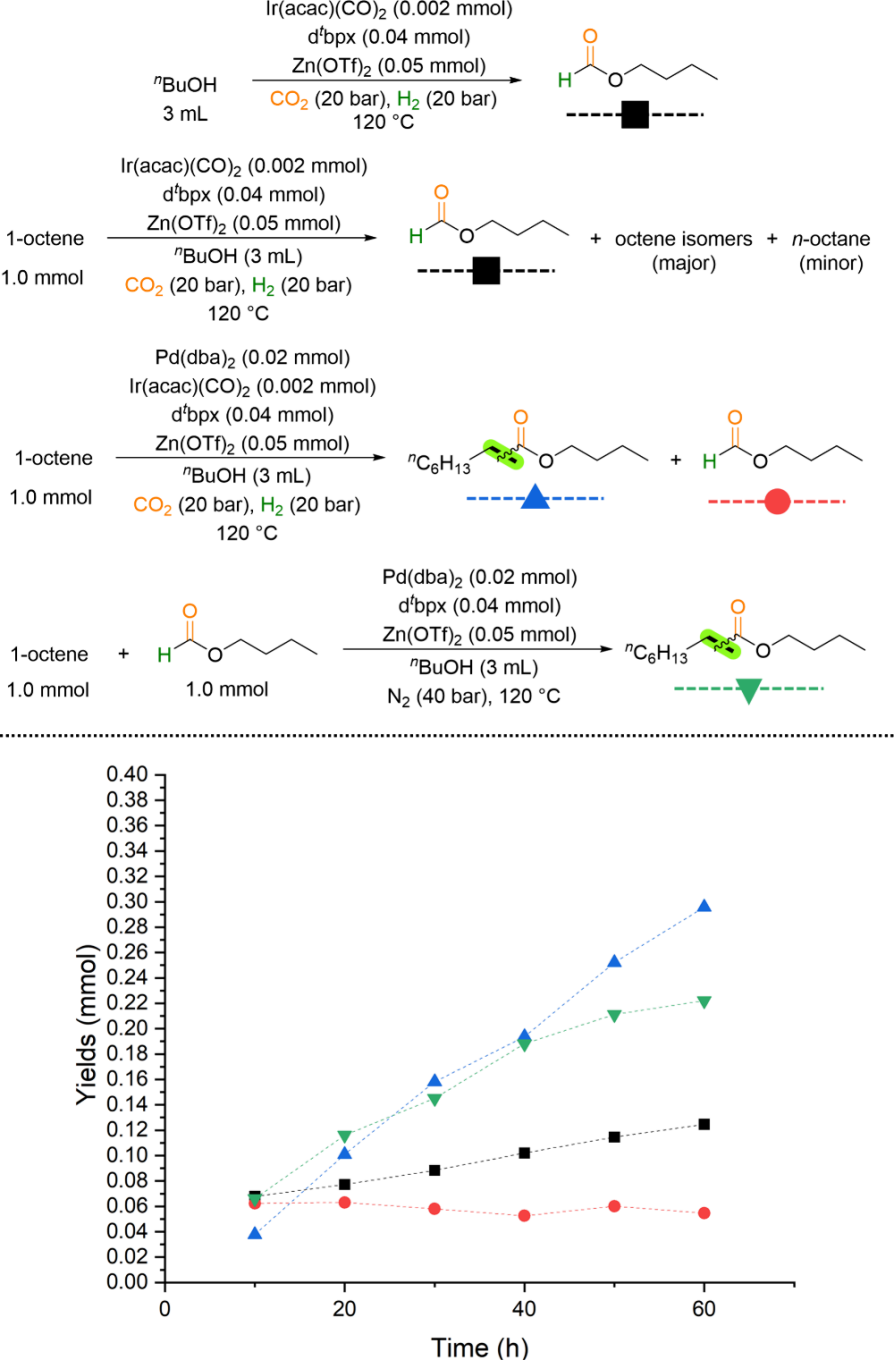
Mechanistic investigations and control experiments.

The corresponding ester product is obtained by addition of palladium additionally to the system under CO_2_/H_2_ (blue line) or in the presence of a stoichiometric amount of butyl formate (green line). Notably, utilizing cyclohexene as substrate, the hydrogenation product cyclohexane could also be quantified. In this latter case, the hydrogenation process even proceeded preferentially. It is noteworthy that comparable yields of butyl nonanoate were observed with butyl formate (utilizing solely Pd) or CO_2_/H_2_ (employing both Pd and Ir) in butanol. In the absence of 1-octene, the amount of butyl formate accumulated by Ir, whereas it was consumed in its presence by Pd. These observations provide unequivocal evidence for the formation of alkyl formate intermediates during this transformation, which undergo direct reactions in subsequent steps. A notable observation is the comparison of the yield of BF in the absence of olefin (black line) to the amounts of ester product in the presence of CO_2_/H_2_ (blue line). This comparison suggests the formation of BF by reduction of CO_2_ in butanol in the presence of the Ir catalyst is a reversible reaction. However, a significant yield (42%, see Supporting Information, Table S6, entry 16) of ester product was obtained when a low concentration of CO (1 bar of CO and 40 bar of N_2_) was applied under the optimized conditions. This suggests that the low partial pressure CO from decomposition of formate can be utilized in the alkxoycarbonylation as well. Thus, in a manner analogous to the preceding experiments for gas phase analysis, a CO_2_/H_2_ mixture with a minimal CO concentration (1.6%) was employed in the optimized conditions for 6 and 20 h within a 25 mL autoclave (see Supporting Information, Figures S6 to S8). Interestingly, the consumption of CO was comparable to the previous results using pure CO_2_/H_2_, with a concentration of approximately 0.2%, and an increase in the ester yield was also observed. To further elucidate the specific roles of the individual constituents of this multicomponent catalyst system, density functional theory (DFT) calculations were performed. Modeling the ligand binding energy indicated that the Lucite ligand exhibited a preference for coordination to palladium over iridium by 36.52 kcal/mol (Supporting Information, Figure S19). Then, the mechanism of the reverse water gas shift (RWGS) process and the effect of Zn­(OTf)_2_ as a Lewis acid additive were systematically computed using HIr­(CO)_4_ as a model catalyst.

The DFT results suggest that the complete RWGS pathway is difficult to realize under current experimental conditions because the apparent barriers of the inevitable dehydration steps are much too high (over 66 kcal/mol) (see Supporting Information, Figures S21 and S22). Furthermore, the addition of zinc as a Lewis acid additive does not appear to reduce the energy barrier (>74 kcal/mol) associated with the dehydration steps (see Supporting Information, Figure S24). However, the transformation of CO_2_ into HCOOH through the CO_2_ → HCOO (**TS6**) → HCOOH (**TS9** for hydrolysis) pathway is thermodynamically more favorable over the RWGS process (9.31 vs 14.25 kcal/mol). Consequently, it is hypothesized that the reaction will reach its conclusion at the HCOOH formation step.

As shown in Figure 5, in the absence of Zn­(OTf)_2_, the catalytic cycle commences with the CO decoordination of the precatalysts (**CAT**, HIr­(CO)_4_), designated as the initiation step, which results in the formation of the **IM1** complex (HIr­(CO)_3_). This process is endothermic by 3.77 kcal/mol. Subsequently, a gas phase CO_2_ molecule interacts with the H­(−Ir) to form the (Ir−)­HCOO group, resulting in **IM9** via a high apparent barrier of 42.06 kcal/mol for **TS6**. This process is endergonic by 10.99 kcal/mol. The **IM9** complex subsequently undergoes a hydrolysis step through the **IM9** → **TS9** → **IM12** pathway, exhibiting an apparent barrier of 40.80 kcal/mol. Subsequently, HCOOH is released from the Ir center, thereby completing the catalytic cycle. It is noteworthy that the presence of Zn­(OTf)_2_, the predominant Lewis acid, results in a significant reduction of the apparent barriers for **TS6** and **TS9**, lowering them to 30.17 and 31.12 kcal/mol, respectively. The incorporation of this Lewis acid led to a substantial reduction in the highest apparent barrier, from 42.06 kcal/mol (**TS6**) to 31.12 kcal/mol (**TS9**), by approximately 11 kcal/mol. This significant decrease in the apparent barrier is expected to result in a substantial reduction in the reaction temperature, thereby enabling the transformation of CO_2_ to HCOOH at a comparably low reaction temperature.

**Figure 5:**
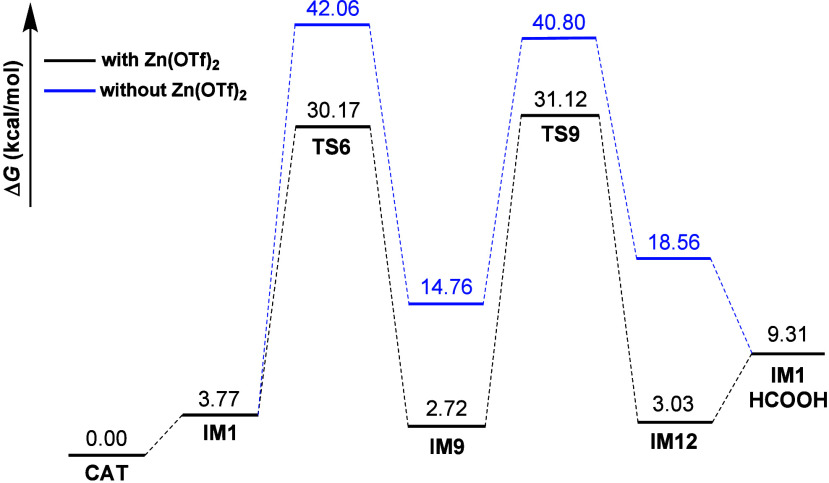
M06L-SCRF computed Gibbs free energy (Δ*G*, 393.15 K) profile of HCOOH formation catalyzed by HIr­(CO)_4_.

Based on the results of the control experiments and DFT calculations, we propose the following mechanism for this one-pot alkoxycarbonylation of olefins with carbon dioxide, as illustrated in Figure 6.

**Figure 6:**
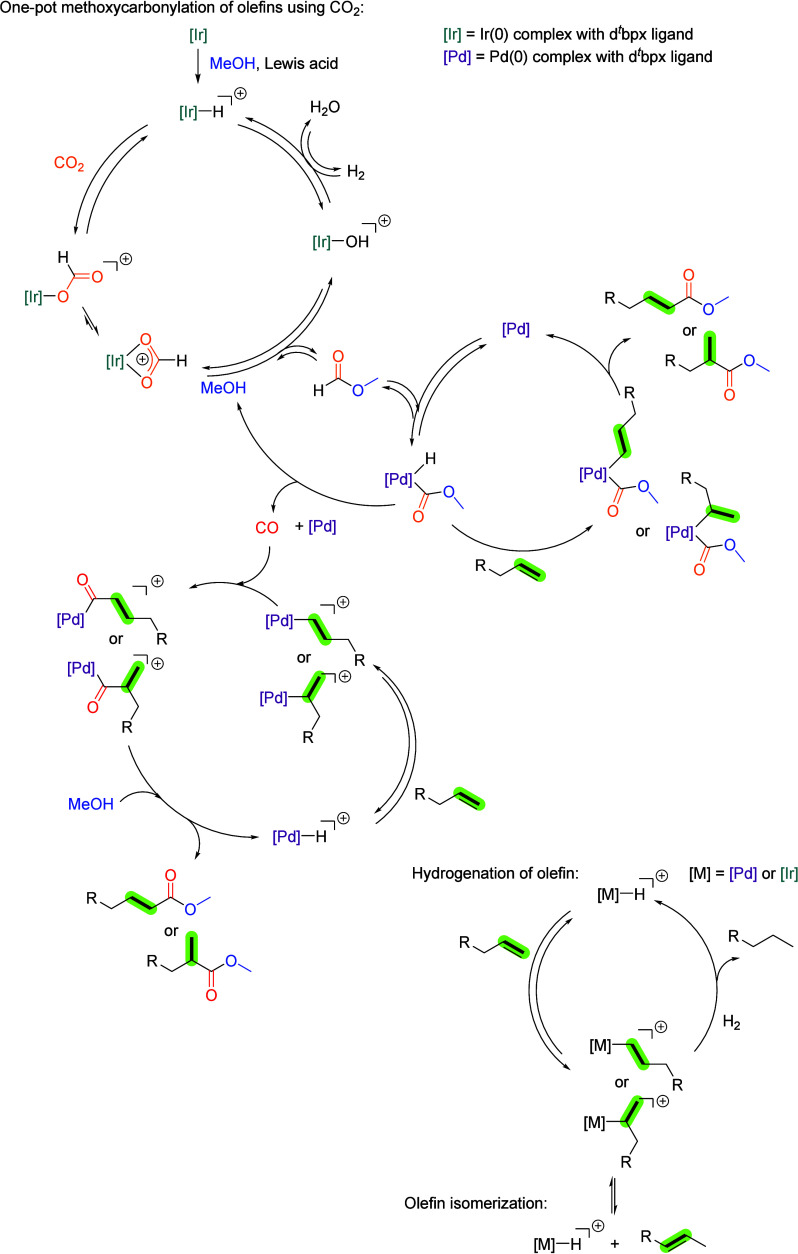
Proposed mechanism of Pd–Ir catalyzed methoxycarbonylation of olefin using CO_2_ and H_2_.

The initial formation of the crucial palladium and iridium hydride species occurs via a Lewis-acid-mediated reaction of Pd(0) and Ir(0) with methanol. Subsequently, hydrogenation of carbon dioxide to formate is catalyzed by an [Ir]–H species, a process that may be enhanced by the presence of the Lewis acidic Zn­(OTf)_2_ additive. Finally, carbonylation of the olefin is achieved through alkyl formate by an active [Pd]–H complex, thereby yielding the desired ester. Alternatively, formate can undergo decomposition back to alcohol, with the released CO being utilized by the carbonylation. In accordance with the findings of previous studies,^11,19^ we hypothesize that the oxidative addition of formate to the palladium catalyst, followed by olefin insertion, constitutes the predominant pathway. A comprehensive mechanistic investigation conducted by Weckhuysen and co-workers on the methoxycarbonylation of 1-octene using paraformaldehyde revealed that no CO gas or palladium carbonyl species was generated or observed in the presence of olefin.^27^ However, methyl formate and CO_2_ were detected, and CO was detected from the decomposition of methyl formate in the absence of 1-octene, leading to a similar mechanistic proposition.

Following the demonstration of the general possibility of achieving a high ester yield and very good regioselectivity in the benchmark reaction, the investigation turned to the generality of this novel methodology. To create a significant impact by utilizing carbon dioxide (and green hydrogen) instead of fossil-based carbon monoxide, industrially relevant alkoxycarbonylations were investigated. The olefinic feedstocks that are of utmost importance to the chemical industry are those that are structurally less complex, such as terminal and internal aliphatic olefins, as illustrated in Scheme 1. Due to its commercial availability, d^*t*^bpx was used as the ligand in these reactions. When applying 2-octene, the internal alkene isomer of 1-octene, a high yield of **2a** (82%) was obtained, accompanied by noteworthy linear selectivity. We also performed one experiment with industrial octene mixtures (see Supporting Information, Table S8). From this, we confirmed that the outcome underscores the viability of olefinic mixtures as a cost-effective feedstock, exhibiting comparable reactivity to pure terminal olefins.

**Scheme 1:**
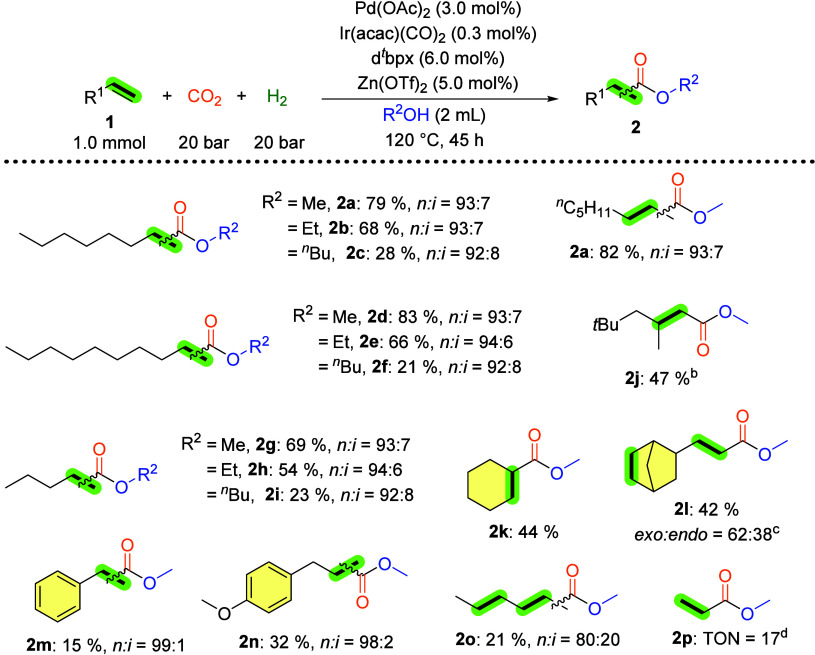
Palladium–Iridium Catalyzed Alkoxycarbonylation of Olefins with CO_2_ and H_2_ ^a^

a Standard reaction conditions: alkene (1.0 mmol), Pd­(OAc)_2_ (3.0 mol %), Ir­(acac)­(CO)_2_ (0.3 mol %), d^*t*^bpx (6.0 mol %), Zn­(OTf)_2_ (5.0 mol %), alcohol (2 mL), CO_2_ (20 bar), H_2_ (20 bar), 120 °C, 45 h reaction time. The isolated yields were obtained by silica gel chromatography, and the regioselectivity of products are determined by ^1^H NMR spectroscopy or GC analysis.
b Diisobutylene substrate, 2,4,4-trimethyl-1-pentene: 2,4,4-trimethyl-2-pentene = 81%: 19%.
c 5-Vinyl-2-norbornene substrate (*exo:endo* = 25:75).
d Methoxycarbonylation of ethylene was carried out in 25 mL autoclave, reaction conditions: Pd­(dba)_2_ (0.025 mmol), d^*t*^bpx (0.05 mmol), Zn­(OTf)_2_ (0.05 mol), MeOH (5 mL), ethylene (5 bar), CO_2_ (15 bar), H_2_ (15 bar), 120 °C, 20 h.

As anticipated, 1-decene, an exemplar of fatty olefins, yielded a substantial amount of the corresponding ester **2d** (83%) with a high linear selectivity. The yield of methyl hexanoates (**2g**) was slightly lower, which could be attributed to the lower boiling point of the 1-pentene substrate. Subsequent reactions of these linear terminal aliphatic olefins with ethanol and *n*-butanol yielded moderate to good yields of the corresponding esters (**2b**–**2i**). Notably, diisobutylene, a substrate employed in industrial carbonylations, yielded **2j** in 47% yield without the need for additional optimization. The isolation of product **2k** was achieved with a comparable yield from cyclohexene. However, here it was observed that a competitive hydrogenation reaction occurred. Notably, the double bond in the cyclic ring of vinyl norbornene was hydrogenated in the obtained corresponding ester **2l**. In contrast, styrene, a model aromatic olefin, exhibited diminished reactivity under the standard reaction conditions. Nevertheless, ester product **2m** was obtained with an exceptional linear-to-branched ratio (*n:i* = 99:1). Intriguingly, upon the application of 2,4-hexadiene, one of the double bonds underwent carbonylation, while another was hydrogenated; however, the yield of methyl heptanoates (**2o**) was not high (21%). Finally, ethylene produced methyl propanoate **2p**, which is a crucial precursor for the industrial synthesis of methyl methacrylate. Further examples can be found in Table S7.

## Summary and Conclusions

In this study, we demonstrate, for the first time, the feasibility of performing catalytic carbonylations of olefins, carbon dioxide, and hydrogen, yielding the corresponding esters with high linear regioselectivity. This is a prerequisite for many practical applications. The success of this unique multimetallic catalyzed transformation is attributed to the synergy of several critical elements: the presence of both active palladium and iridium species in the presence of 1,2-bis­(di-*tert*-butyl­phos­phino­methyl)­benzene (d^*t*^bpx) as the ligand and the specific Lewis acid additive Zn­(OTf)_2_.

The outcome of this transformation is the preparation of industrially relevant esters, characterized by their exceptional linear selectivity, directly from carbon dioxide. Kinetic studies and control experiments essentially confirm the formation of alkyl formate as crucial intermediates in the reaction sequence, bridging the reduction of CO_2_ and the carbonylation of olefins. Our findings, together with the observed acceleration of alkoxycarbonylation in the presence of low concentrations of CO, support a mechanistic scenario in which methyl formate acts primarily as a reservoir for the gradual generation of CO, which is then engaged in the palladium-catalyzed carbonylation, rather than being directly valorized. Of particular interest is the detection of only trace amounts of CO (approximately 0.2%) in the gas phase of the reaction, a finding that facilitates carbonylations in low CO concentration. This mechanistic insight aligns with established metal-mediated decomposition of alkyl formates and underscores the relevance of conventional carbonylation chemistry in our system. It is anticipated that this paradigm will serve as an inspiration for chemists in both industry and academia to perform more often green carbonylations, thereby contributing to the transformation of the chemical industry toward greater sustainability.

## Supplementary Material



## Notes

Experimental procedures, spectroscopic data, and DFT calculations (PDF)
