# Supplementary material for: Highly Selective Carbonylation of Olefins Using CO2 and H2
Source: J Am Chem Soc. 2025 Aug 28;147(36):32873–82. doi: 10.1021/jacs.5c09325 (PMC12426881; doi:10.1021/jacs.5c09325)
Supplement: Supplementary file 1 [file ja5c09325_si_001.pdf]

Highly selective carbonylation of olefins using CO_2 and H_2

Mohamed Niyaz Vellala Syed Ali[‡], Weiheng Huang[‡], Xinxin Tian[†††], Haijun Jiao*, Ralf Jackstell*, Robert Franke*[,†,††] and Matthias Beller*

Leibniz-Institut für Katalyse e. V. Albert-Einstein-Straße 29a,18059 Rostock (Germany)

[†]Evonik Oxeno GmbH, Paul-Baumann-Straße 1, 45772 Marl, Germany

[††]Lehrstuhl für Theoretische Chemie, 44780 Bochum, Germany

[†††]Institute of Molecular Science, Shanxi University, Taiyuan 030006, China

[‡] These authors contributed equally to this work.

*Corresponding author. Email: matthias.beller@catalysis.de; ralf.jackstell@catalysis.de; haijun.jiao@catalysis.de; robert.franke@evonik.com

Table of Contents

1. General information: Materials, experimental and computational methods S2
2. Synthesis of ligands S3
3. Non-active ligands for palladium-iridium catalyzed alkoxycarbonylation of 1-octene using CO_2 .... S5
4. Supplementary results of reaction condition optimization S6
5. Gas components analysis experiments S12
6. Preliminary reaction pathway investigation S20
7. Theoretical reaction pathway investigation S26
8. NMR data S33
9. NMR spectra S39
10. Supplementary References S69

1. General information: Materials, experimental and computational methods

All commercial reagents were ordered from Alfa Aesar, Abcr, Aldrich, TCI or Strem, CO (4.7) gas was ordered by Linde. Unless otherwise stated, commercial reagents were used without purification. Dry solvents were prepared according to standard procedures.[33] Air- and moisture-sensitive syntheses were performed under argon atmosphere in glassware after heating gun dried under vacuum. Analytical data of literature known compounds were in accordance with reported data. NMR spectra were recorded on Bruker Avance 300 (300 MHz) NMR spectrometers. Multiplets were assigned as s (singlet), d (doublet), t (triplet), dd (doublet of doublet), m (multiplet) and br. s (broad singlet). All measurements were carried out at room temperature unless otherwise stated. Gas Chromatography analysis in performed by an HP 6890 chromatograph with a 29 m HP5 column was used. Linear to branched ratios were determined by GC analysis of the crude reaction mixture. The products were isolated from the mixture by following column chromatography on silica gel 60, 0.064-0.2 mm, 70-230 mesh (Merk). Gas GC analysis is performed by Agilent Technologies 7890A GC system (HP Plot Q / FID – hydrocarbons, Carboxen / TCD - permanent gases, He carrier gas). The CO quantification limit is 10 ppm. Specially, all the olefins are purified by distillation or silica gel chromatography before testing.

Detailed procedure for the alkoxycarbonylation of 1-octene (1.0 mmol scale reaction): The solid starting materials palladium precursor, iridium precursor, ligand, and additive were weighed and added to a 12 mL screw-cap vial with 6 mm magnetic stirring bar in glovebox. The vial was sealed by a Teflon/Silicone septum and phenolic plastic cap before transferring out of the glovebox. The vial was connected to Schlenk line with a needle under argon flow. Specific amount of distilled alcohol solution and distilled 1-octene (157 µL, 1.0 mmol) were injected by normal syringe and microliter syringe respectively. The vial was held on an alloy plate and put into a Parr 4560 series autoclave (300 mL). At room temperature, the autoclave was flushed with nitrogen gas for twice. Carbon dioxide was also flushed twice and charged to specific pressure, then hydrogen was fed to a specific total pressure. The reaction was heated to specific temperature and stirred (600 rpm) for specific reaction time. Afterwards, the autoclave was cooled to by ice water then the gas was carefully released. Isooctane (83 µL, 0.5 mmol) was added to the solution as internal standard, and the solution was filtered by celite and analyzed by gas chromatography.

Methoxycarbonylation of ethene: The solid starting materials palladium precursor $Pd(OAc)_2$ (6.7 mg, 0.03 mmol), iridium precursor $Ir(acac)(CO)_2$ (1.04 mg, 0.003 mmol), (ligand 1,2-bis(di-*tert*-butylphosphinomethyl)benzene (d^tbpx) (23.7 mg, 0.06 mmol) and additive $Zn(OTf)_2$ (18.2 mg, 0.05 mmol) were weighed and added to a 10 mL Schlenk flask in glovebox. Distilled methanol (5.0 mL) was added to the Schlenk flask by normal syringe under argon. After all materials were dissolved, the methanol solution was transferred to a 25 mL Parr series 4790 autoclave under argon flow. At room temperature, ethene gas was firstly fed to 10 bar, then carbon dioxide was charged to 25 bar, hydrogen was fed to a total pressure of 40 bar at last. The reaction was heated to 120 °C and stirred (800 rpm) for 20 hours. Afterwards, the autoclave was cooled to by ice water then the gas was carefully released. Isooctane (83 µL, 0.5 mmol) was added to the solution as internal standard, and the solution was filtered by celite and analyzed by gas chromatography.

Computational methods: DFT calculations were performed with Gaussian 16.[28] Geometries were optimized and characterized by frequency calculations at the M06L[29]/BS level in gas phase (BS, LANL2DZ[30] for iridium and TZVP[31] for other elements). Single point energy calculations in polar solvent of methanol were then done by using the SMD model[32] at the same level (M06L-SCRF/BS//M06L/BS). To fit the experimental temperature, all energies for discussion and

comparison were corrected to Gibbs free energies at 393.15 K (120 °C) and 1 atm. All detailed information and results are summarized in the Supporting Information.

2. Synthesis of ligands

The BPX and L1 ligand were synthesized according to literature.[34] The solvents used are all dried and degassed.

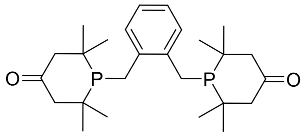

1,2-bis[(2,2,6,6-tetramethylphosphinan-4-onyl)methyl]benzene (BPX)

o-Xylylene dichloride (3.5 g, 20 mmol) and triethyl phosphite (13.9 mL, 80 mmol) were added in a Schlenk flask with a magnetic stirring bar. Quantitative yield of o-xylylendiphosphonsaeuretetraethylester was obtained after refluxing at 120 °C for 16 hours, the extra triethyl phosphite was removed by vacuum. In another Schlenk flask with a magnetic stirring bar, $LiAlH_4$ (341 mg, 9 mmol) and THF (20 mL) was added and cooled to -78 °C, then Me_3SiCl (1.2 mL, 9 mmol) was added dropwise. o-Xylylendiphosphonsaeuretetraethylester (756 mg, 2 mmol) was dissolved in THF (10 mL), and this solution was added to the $LiAlH_4$-Me_3SiCl THF suspension slowly. Let the mixture warm up to room temperature and stir for overnight. The mixture was quenched dropwise by degas water (0.5 mL) at ice water bath, anhydrous Na_2SO_4 was added to dry the mixture. Then it was filter by Al_2O_3, 1,2-bis(phosphinomethyl)benzene was obtained after evaporating all THF. 2,6-dimethyl-2,5-heptadien-4-one (0.65 mL, 4 mmol) was mixed with 1,2-bis(phosphinomethyl)benzene and refluxed at 120 °C for overnight. After the mixture back to room temperature, it was washed by cool methanol. A white solid was obtained by recrystallization in methanol. 1,2-bis[(2,2,6,6-tetramethylphosphinan-4-onyl)methyl]benzene (BPX) was obtained in 29 % yield (131 mg) after removing methanol.

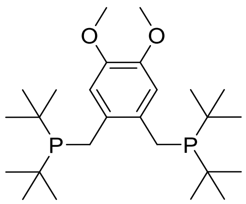

((4,5-(dimethoxy)-1,2-phenylene)bis(methylene))bis(di-*tert*-butylphosphine) (L1)

1,2-dimethoxybenzene (1.3 mL, 10 mmol) and paraformaldehyde (0.6 g, 20 mmol) were added into a Schlenk flask with a magnetic stirring bar. Acetic acid (6 mL) and HBr in acetic acid (33 %) were added into the Schlenk flask and stirred for overnight at room temperature, white precipitate

was formed. The mixture was filtered and washed by petroleum ether, then dried under vacuum. 1,2-bis(bromomethyl)-4,5-dimethoxybenzene (1.46 g, 46 %) was obtained after a fast silica gel chromatography (acetone : pentane = 1 : 5). Then 10 equivalents of LiCl (2.1 g) were mixed with 1,2-bis(bromomethyl)-4,5-dimethoxybenzene and dissolved in DMF (8 mL) and stirred for 24 hours at room temperature. Quantitative yield of 4,5-Bis-chlormethyl-veratrol (1.08 g) was obtained after extraction with ethyl acetate and water. Di-*tert*-butylphosphine (0.82 mL, 4.4 mmol) was deprotonated by *n*-BuLi (2.5 M in hexane, 1.8 mL) in THF (20 mL) at -78 °C. Let the solution warm up to room temperature and stir for 2 hours. 4,5-Bis-chlormethyl-veratrol (468.04 mg, 2.0 mmol) was dissolved in THF (10 mL), then it was added dropwise to the $t\mathrm{Bu_2PLi}$ solution at -78 °C. Let it stir at -78 °C for 30 min then warm up to room temperature for overnight. THF was evaporated under vacuum, and the residue was extracted and filtered by heptane. Then heptane was removed under vacuum and the mixture was recrystallized in methanol. To get the pure ligand, the sample was recrystallized for 3 times leading a 8 % yield of product (108 mg).

3. Non-active ligands for palladium-iridium catalyzed alkoxycarbonylation of 1-octene using CO_2.

Table S1 | Non-active ligands for palladium-iridium catalyzed alkoxycarbonylation of 1-octene using CO_2.

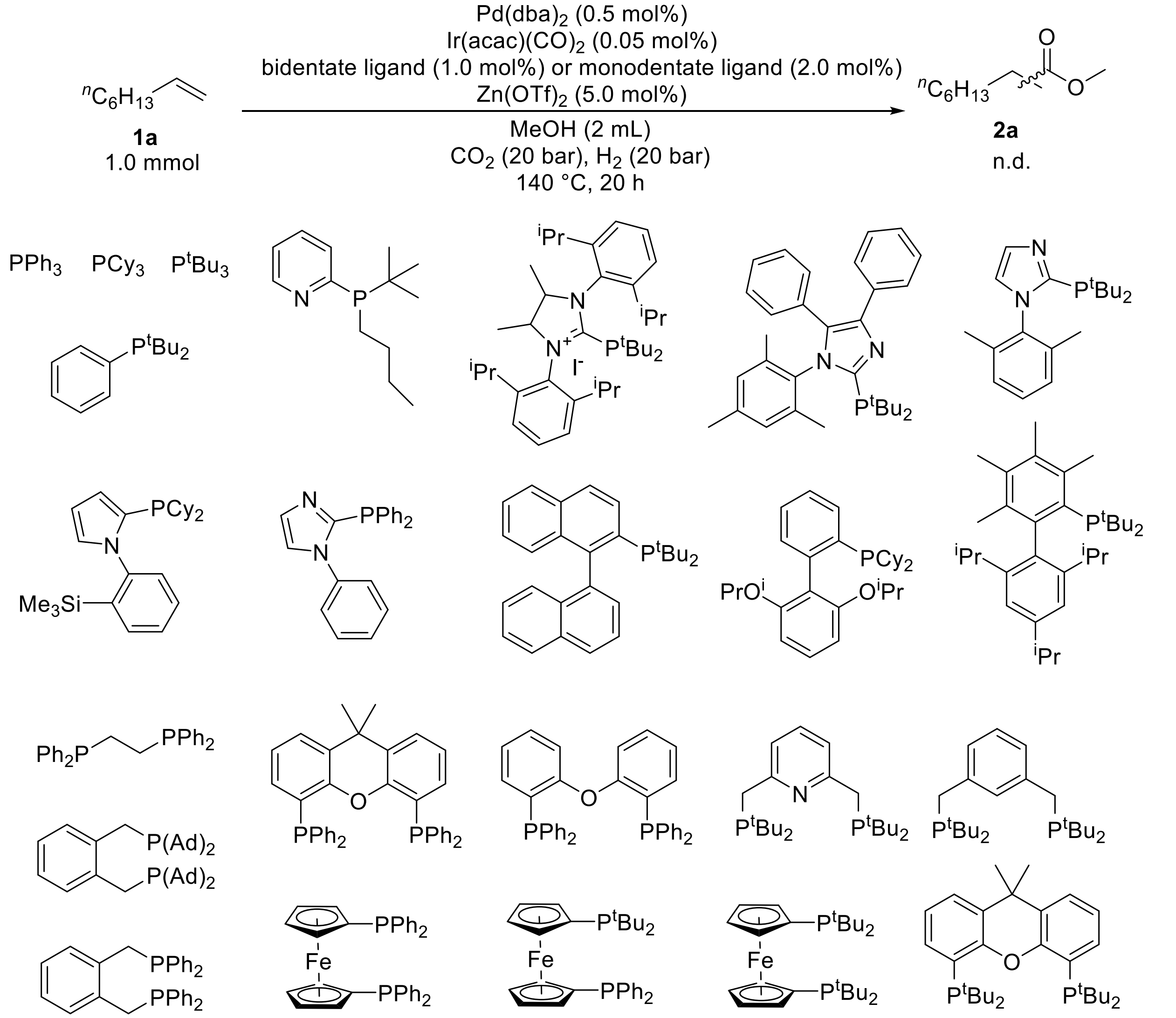

4. Supplementary results of reaction condition optimization

Table S2 | Effect of additives

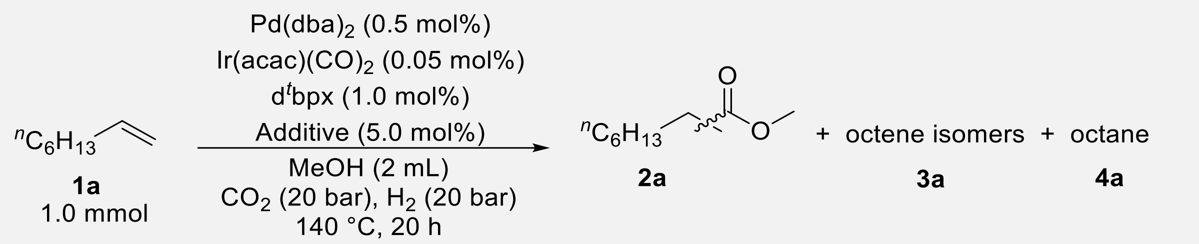

Entry	Additives	Yield of 2a	*n:i*	Yield of 3a and 4a
1	$Sc(OTf)_3$	-	-	99 % (major **4a**)
2	$La(OTf)_3$	-	-	99 % (major **4a**)
3	$Sn(OTf)_2$	-	-	99 % (major **4a**)
4	$Zn(NO_3)_2$	-	-	98 % (major **3a**)
5	$ZnSO_4$	-	-	98 % (major **3a**)
6	$Zn(acac)_2$	-	-	98 % (major **3a**)

Reaction conditions: **1a** (1.0 mmol), $Pd(dba)_2$ (0.5 mol%), $Ir(acac)(CO)_2$ (0.05 mol%), d^{t}bpx (1.0 mol%), additives (5.0 mol%), MeOH (2 mL), CO_2 (20 bar), H_2 (20 bar), 140 °C, 20 hours reaction time. The ratio of linear to all branched products = *n:i*. Yields and *n:i* are determined by GC analysis using isooctane (56 mg, 0.5 mmol) as internal standard.

Table S3 | Effect of [Pd]:[L]:[Zn(OTf)$_2$]

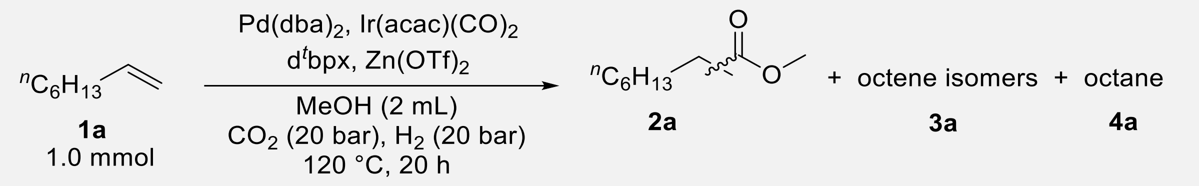

Entry	[Ir]:[Pd]:[d^{t}bpx]:[Zn(OTf)$_2$] (mol%)	Yield of 2a	*n:i*	Yield of 3a and 4a
1	0.01 : 0.5 : 1.0 : 5.0	< 1 %	-	98 % (major **3a**)
2	0.025 : 0.5 : 1.0 : 5.0	6 %	91:9	92 % (major **3a**)
3	0.05 : 0.5 : 1.0 : 5.0	25 %	91:9	74 % (major **3a**)
4	0.075 : 0.5 : 1.0 : 5.0	14 %	91:9	84 % (major **4a**)
5	0.1 : 0.5 : 1.0 : 5.0	5 %	92:8	93 % (major **4a**)
6	0.05 : 0.5 : 1.0 : 2.0	12 %	93:7	87 % (major **3a**)
7	0.05 : 0.5 : 1.0 : 10.0	30 %	91:9	70 % (major **3a**)
8	0.05 : 0.5 : 1.0 : 20.0	30 %	91:9	70 % (major **3a**)
9	0.05 : 0.5 : 1.0 : 50.0	23 %	91:9	75 % (major **4a**)
10	0.05 : 0.5 : 0.5 : 5.0	8 %	93:7	91 % (major **3a**)
11	0.05 : 0.5 : 2.0 : 5.0	25 %	92:8	75 % (major **3a**)
12	0.2 : 2.0 : 4.0 : 20.0	46 %	92:8	55 % (major **4a**)
13	0.2 : 2.0 : 4.0 : 10.0	66 %	92:8	44 % (major **3a**)
14	0.2 : 2.0 : 4.0 : 1.0	61 %	93:7	38 % (major **3a**)
15	0.2 : 2.0 : 5.0 : 2.0	69 %	92:8	30 % (major **3a**)

16	0.2 : 2.0 : 8.0 : 5.0	74 %	92:8	26 % (major **3a**)
17	0.3 : 3.0 : 6.0 : 5.0	68 %	92:8	31 % (major **4a**)

Reaction conditions: **1a** (1.0 mmol), Pd(dba)$_2$, Ir(acac)(CO)$_2$, d*t*bpx, Zn(OTf)$_2$, MeOH (2 mL), CO_2 (20 bar), H_2 (20 bar), 120 °C, 20 hours reaction time. The ratio of linear to all branched products = *n:i*. Yields and *n:i* are determined by GC analysis using isooctane (56 mg, 0.5 mmol) as internal standard.

Table S4 | Effect of Pressure and Temperature

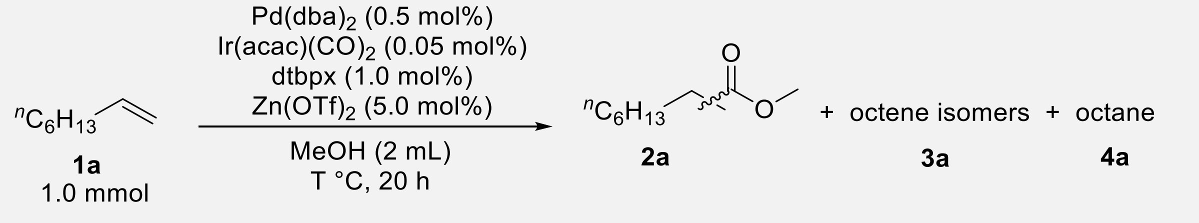

Entry	T °C, CO_2:H_2 (bar)	Yield of 2a	*n:i*	Yield of 3a and 4a
1	120 °C, 20:20	25 %	92:8	75 % (major **3a**)
2	100 °C, 20:20	9 %	92:8	75 % (major **3a**)
3	140 °C, 30:10	25 %	91:9	75 % (major **3a**)
4	140 °C, 10:30	23 %	91:9	76 % (major **3a**)
5	140 °C, 10:10	13 %	93:7	86 % (major **3a**)
6[a]	120 °C, 35:5	66 %	93:7	33 % (major **3a**)
7[a]	120 °C, 5:35	3 %	95:5	95 % (major **3a**)
8[a]	100 °C, 20:20	17 %	96:4	82 % (major **3a**)

Reaction conditions: 1a (1.0 mmol), $Pd(dba)_2$ (0.5 mol%), $Ir(acac)(CO)_2$ (0.05 mol%), d^tbpx (1.0 mol%), $Zn(OTf)_2$ (5.0 mol%), MeOH (2 mL), CO_2, H_2, T °C, 20 hours reaction time. The ratio of linear to all branched products = *n:i*. Yields and *n:i* are determined by GC analysis using isooctane (56 mg, 0.5 mmol) as internal standard. a) $Pd(dba)_2$ (2.0 mol%), $Ir(acac)(CO)_2$ (0.2 mol%), d^tbpx (4.0 mol%).

Table S5 | Effect of co-solvents

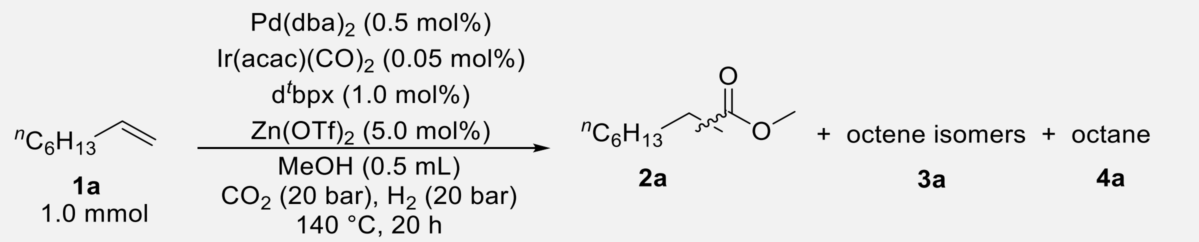

Entry	co-solvent (1.5 mL)	Yield of 2a	*n:i*	Yield of 3a and 4a
1	Toluene	3 %	94:6	95 % (major **3a**)
2	Heptane	-	-	99 % (major **3a**)
3	THF	1 %	-	99 % (major **3a**)
4	Dioxane	< 1 %	-	99 % (major **3a**)
5	DMF	-	-	99 % (major **3a**)
6	DCM	-	-	99 % (major **4a**)
7	Anisole	-	-	99 % (major **3a**)
8	MeCN	< 1 %	-	99 % (major **3a**)

Reaction conditions: **1a** (1.0 mmol), $Pd(dba)_2$ (0.5 mol%), $Ir(acac)(CO)_2$ (0.05 mol%), d^tbpx (1.0 mol%), $Zn(OTf)_2$ (5.0 mol%), MeOH (0.5 mL), co-solvent (1.5 mL), CO_2 (20 bar), H_2 (20 bar), 140 °C, 20 hours reaction time. The ratio of linear to all branched products = *n:i*. Yields and *n:i* are determined by GC analysis using isooctane (56 mg, 0.5 mmol) as internal standard.

Table S6 | Further investigation of reaction conditions

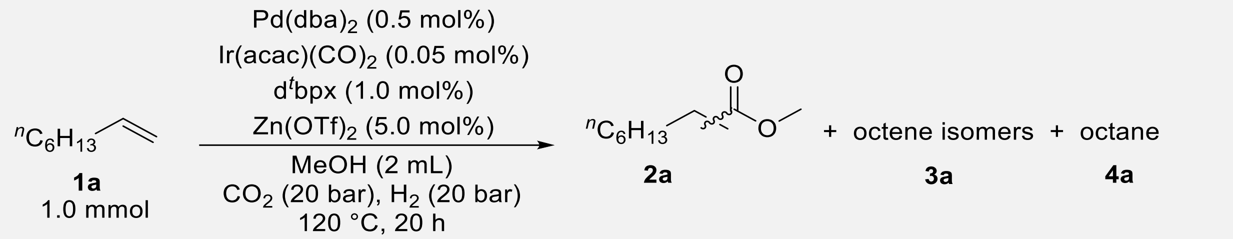

Entry	Variation	Yield of 2a	*n:i*	Yield of 3a and 4a
1	-	25 %	91:9	74 % (major **3a**)
2	$[Ir(cod)Cl]_2$	3 %	92:8	96 % (major **4a**)
3	$IrCl_3$	-	-	98 % (major **4a**)
4	$Ir_4(CO)_{12}$	26 %	92:8	73 % (major **3a**)
5	$Pd(cod)Cl_2$	6 %	95:5	93 % (major **4a**)
6	$PdBr_2$	-	-	97 % (major **4a**)
7	$Pd(OAc)_2$	25 %	93:7	74 % (major **3a**)
8	45 h	33 %	93:7	66 % (major **3a**)
9	70 h	47 %	93:7	52 % (major **3a**)
10	(a)	66 %	93:7	33 % (major **3a**)
11	(a), 45 h	74 %	93:7	25 % (major **3a**)
12	(b), 45 h	77 %	93:7	20 % (**4a**)
13	(c), 45 h	83 %	92:8	16 % (**4a**)
14	(d), 45 h	80 %	93:7	19 % (**4a**)
15	(e), 45 h	84 %	92:8	15 % (**4a**)
16	(a), CO (1 bar) and N_2 (40 bar) i.o. CO_2 and H_2	42 %	93:7	57 % (**3a**)
17	(b), **1a** (250.0 mmol), 45 h	70 %	93:7	27 % (**4a**)

Reaction conditions: **1a** (1.0 mmol), $Pd(dba)_2$ (0.5 mol%), $Ir(acac)(CO)_2$ (0.05 mol%), d^{t}bpx (1.0 mol%), $Zn(OTf)_2$ (5.0 mol%), MeOH (2 mL), CO_2 (20 bar), H_2 (20 bar), 120 °C, 20 hours reaction time. The ratio of linear to all branched products = *n:i*. Yields and *n:i* are determined by GC analysis using isooctane (56 mg, 0.5 mmol) as internal standard.
(a): $Pd(dba)_2$ (2.0 mol%), $Ir(acac)(CO)_2$ (0.2 mol%), d^{t}bpx (4.0 mol%).
(b): $Pd(dba)_2$ (3.0 mol%), $Ir(acac)(CO)_2$ (0.3 mol%), d^{t}bpx (6.0 mol%).
(c): $Pd(dba)_2$ (3.0 mol%), $Ir(acac)(CO)_2$ (0.3 mol%), **L1** (6.0 mol%).
(d): $Pd(OAc)_2$ (3.0 mol%), $Ir(acac)(CO)_2$ (0.3 mol%), d^{t}bpx (6.0 mol%).
(e): $Pd(OAc)_2$ (3.0 mol%), $Ir(acac)(CO)_2$ (0.3 mol%), **L1** (6.0 mol%).

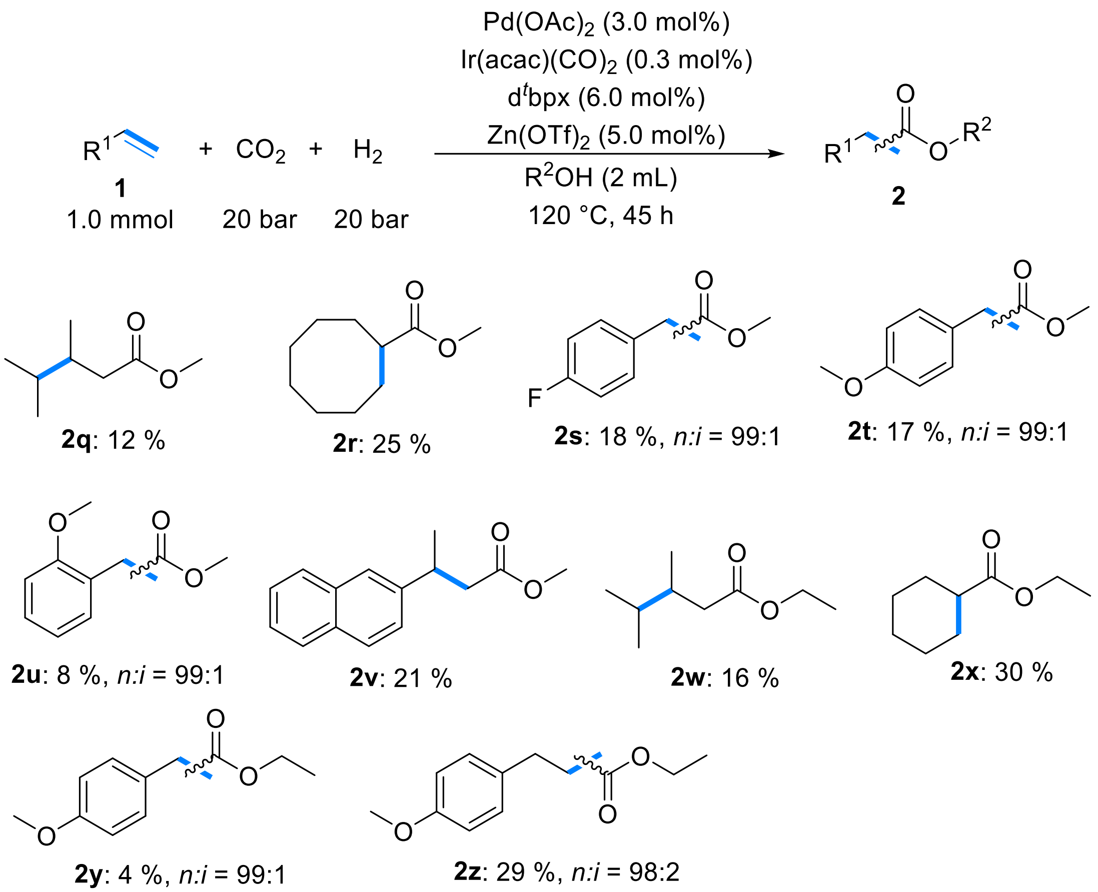

Table S7 | Other alkenes for palladium-iridium catalyzed alkoxycarbonylation with CO_2 and H_2. Standard reaction conditions: alkene (1.0 mmol), $Pd(OAc)_2$ (3.0 mol%), $Ir(acac)(CO)_2$ (0.3 mol%), d^{t}bpx (6.0 mol%), $Zn(OTf)_2$ (5.0 mol%), alcohol (2 mL), CO_2 (20 bar), H_2 (20 bar), 120 °C, 45 hours reaction time. The isolated yields were obtained by silica gel chromatography, and the regioselectivity of products are determined by ^{1}H NMR spectroscopy or GC analysis.

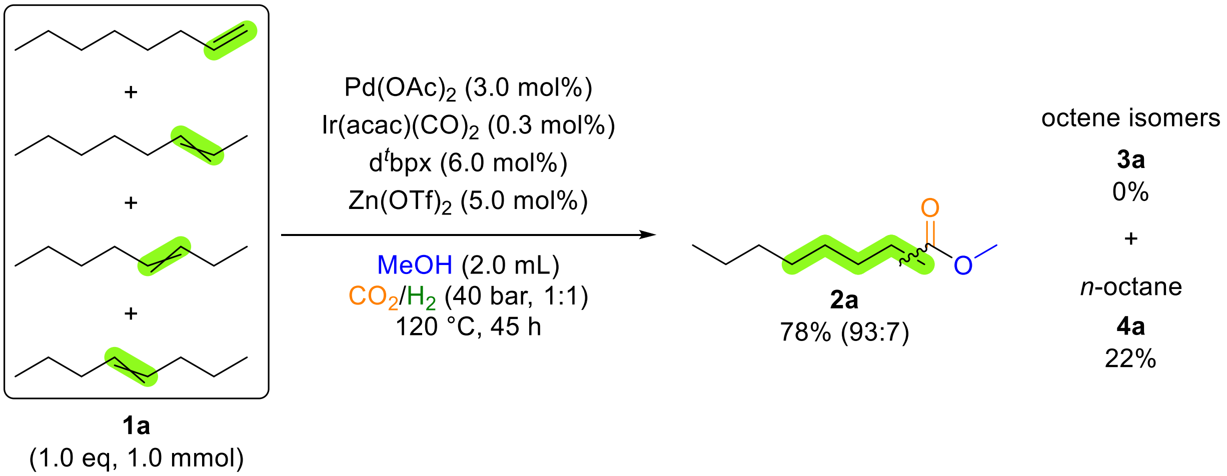

Table S8 | Industrial mixture of octenes for palladium-iridium catalyzed alkoxycarbonylation with CO_2 and H_2. Standard reaction conditions: 1a (1.0 mmol), $Pd(OAc)_2$ (3.0 mol%), $Ir(acac)(CO)_2$ (0.3 mol%), d^{t}bpx (6.0 mol%), $Zn(OTf)_2$ (5.0 mol%), MeOH (2 mL),

CO_2 (20 bar), H_2 (20 bar), 120 °C, 45 hours reaction time. The composition of 1a (industrial octene mixtures) are as follows:

1-octene = 6%

2-octene (E:Z) = 47% (72:28)

3-octene (E:Z) = 34% (81:19)

4-octene (E:Z) = 13% (100:0)

The composition of industrial octene mixtures, yields, and ratio of linear to all branched products = *n:i* all are determined by GC analysis using isooctane (56 mg, 0.5 mmol) as internal standard.

5. Gas components analysis experiments

Gas GC analysis is performed by: Agilent Technologies 7890A GC system (HP Plot Q / FID – hydrocarbons, Carboxen / TCD - permanent gases, He carrier gas). The CO quantification limit is 10 ppm, the H_2 and CO_2 quantification limit are 0.1 %.

```
Data File C:\CHEM32\2\DATA\2023\2309\230922\SIG1011737.D
Sample Name: CO2&H2-1.131

    =====================================================================
    Acq. Operator   : wh
    Acq. Instrument : GC 3 Lab 1.126                  Location : Vial 1
    Injection Date  : 9/22/2023 11:26:15 AM                Inj :   1
                                                    Inj Volume : Manually
    Acq. Method     : C:\CHEM32\2\METHODS\LUTZ1.M
    Last changed    : 9/27/2021 3:32:42 PM by dw
    Analysis Method : C:\CHEM32\2\METHODS\CAL_2019.M
    Last changed    : 9/25/2023 12:21:05 PM by wh
                      (modified after loading)
```

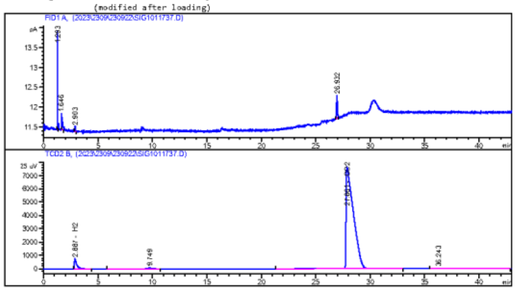


```
    =====================================================================
                        External Standard Report
    =====================================================================

    Sorted By             :      Retention Time
    Calib. Data Modified  :      9/25/2023 12:21:08 PM
    Multiplier            :      1.0000
    Dilution              :      1.0000
    Use Multiplier & Dilution Factor with ISTDs

    Signal 1: FID1 A,
    Signal 2: TCD2 B,
```

RetTime [min]	Sig	Type	Area	Amt/Area	Amount [vol%]	Grp	Name
2.887	2	BB	1.01767e4	4.44130e-3	45.19797		H2
8.050	2		-	-	-		Ar
12.290	2		-	-	-		CO
21.000	2		-	-	-		CH4
27.861	2	BB	3.56940e5	1.55700e-4	55.57559		CO2

Totals : 100.77356

GC 3 Lab 1.126 9/25/2023 12:23:01 PM wh   Page 1 of 2

```
Data File C:\CHEM32\2\DATA\2023\2309\230922\SIG1011737.D
Sample Name: CO2&H2-1.131

    1 Warnings or Errors :

    Warning : Calibrated compound(s) not found

    =====================================================================
                        *** End of Report ***
```

GC 3 Lab 1.126 9/25/2023 12:23:01 PM wh   Page 2 of 2

Fig. S1 | Applied carbon dioxide and hydrogen gas. There is no detectable CO (< 10 ppm).

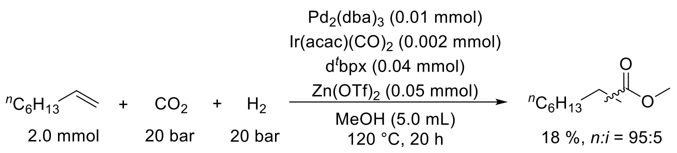

Data File C:\CHEM32\2\DATA\2023\2309\230927\SIG1011763.D
Sample Name: whh1075-4

===
Acq. Operator : WH
Acq. Instrument : GC 3 Lab 1.126 Location : Vial 1
Injection Date : 9/27/2023 6:27:31 PM Inj : 1
Inj Volume : Manually
Acq. Method : C:\CHEM32\2\METHODS\LUTZ1.M
Last changed : 9/27/2021 3:32:42 PM by dw
Analysis Method : C:\CHEM32\2\METHODS\CAL_2019.M
Last changed : 9/28/2023 9:46:09 AM by wh
(modified after loading)
Additional Info : Peak(s) manually integrated

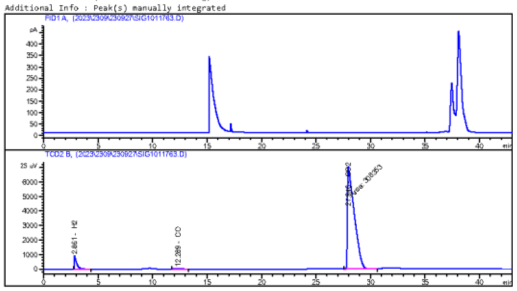

External Standard Report

Sorted By : Retention Time
Calib. Data Modified : 9/28/2023 9:46:12 AM
Multiplier : 1.0000
Dilution : 1.0000
Use Multiplier & Dilution Factor with ISTDs

Signal 1: FID1 A,
Signal 2: TCD2 B,

RetTime [min]	Sig	Type	Area	Amt/Area	Amount [vol%]	Grp	Name
2.861	2	BB	1.27654e4	4.39150e-3	56.05940		H2
8.050	2		-	-	-		Ar
12.289	2	BB	775.94427	2.12647e-4	1.65002e-1		CO
21.000	2		-	-	-		CH4
27.946	2	MM	3.08353e5	1.54756e-4	47.71947		CO2

GC 3 Lab 1.126 9/28/2023 9:46:17 AM wh Page 1 of 2

Data File C:\CHEM32\2\DATA\2023\2309\230927\SIG1011763.D
Sample Name: whh1075-4

Totals : 103.94387

1 Warnings or Errors :

Warning : Calibrated compound(s) not found

*** End of Report ***

GC 3 Lab 1.126 9/28/2023 9:46:17 AM wh Page 2 of 2

CO concentration = 0.165002 / 103.94387 = 0.159 %.

Fig. S2 | Gas components analysis after a 20 h methoxycarbonylation of 1-octene reaction in 25 mL autoclave. Reaction conditions: 1-octene (2.0 mmol), $Pd_2(dba)_3$ (0.01 mmol), $Ir(acac)(CO)_2$ (0.002 mmol), d^{t}bpx (0.04 mmol), $Zn(OTf)_2$ (0.05 mmol), MeOH (5.0 mL), CO_2 (20 bar), H_2 (20 bar), 120 °C, 20 h.

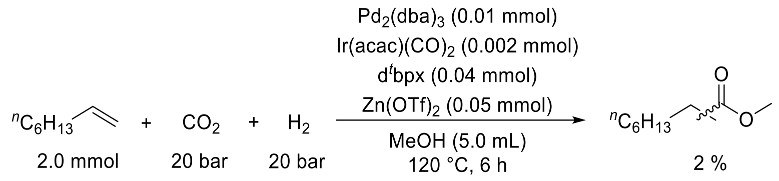

Data File C:\CHEM32\2\DATA\2023\2309\230925\SIG1011751.D
Sample Name: whh1075-2

===
Acq. Operator : wh
Acq. Instrument : GC 3 Lab 1.126 Location : Vial 1
Injection Date : 9/25/2023 5:35:43 PM Inj : 1
Inj Volume : Manually
Acq. Method : C:\CHEM32\2\METHODS\LUTZ1.M
Last changed : 9/27/2021 3:32:42 PM by dw
Analysis Method : C:\CHEM32\2\METHODS\CAL_2019.M
Last changed : 9/25/2023 6:26:44 PM by wh
(modified after loading)
Additional Info : Peak(s) manually integrated

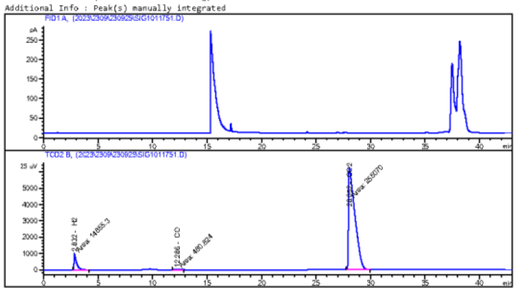

External Standard Report

Sorted By : Retention Time
Calib. Data Modified : 9/25/2023 6:26:48 PM
Multiplier : 1.0000
Dilution : 1.0000
Use Multiplier & Dilution Factor with ISTDs

Signal 1: FID1 A,
Signal 2: TCD2 B,

RetTime [min]	Sig	Type	Area	Amt/Area	Amount [vol%]	Grp	Name
2.832	2	MM	1.48553e4	4.35292e-3	64.66384		H2
8.050	2		-	-	-		Ar
12.286	2	MM	480.82401	2.08743e-4	1.00369e-1		CO
21.000	2		-	-	-		CH4
28.052	2	MM	2.55070e5	1.53746e-4	39.21585		CO2

GC 3 Lab 1.126 9/25/2023 6:26:50 PM wh Page 1 of 2

Data File C:\CHEM32\2\DATA\2023\2309\230925\SIG1011751.D
Sample Name: whh1075-2

Totals : 103.98005

1 Warnings or Errors :

Warning : Calibrated compound(s) not found

*** End of Report ***

GC 3 Lab 1.126 9/25/2023 6:26:50 PM wh Page 2 of 2

CO concentration = 0.100368 / 103.98005 = 0.097 %.

Fig. S3 | Gas components analysis after a 6 h methoxycarbonylation of 1-octene reaction in 25 mL autoclave. Reaction conditions: 1-octene (2.0 mmol), $Pd_2(dba)_3$ (0.01 mmol), $Ir(acac)(CO)_2$ (0.002 mmol), d^{t}bpx (0.04 mmol), $Zn(OTf)_2$ (0.05 mmol), MeOH (5.0 mL), CO_2 (20 bar), H_2 (20 bar), 120 °C, 6 h.

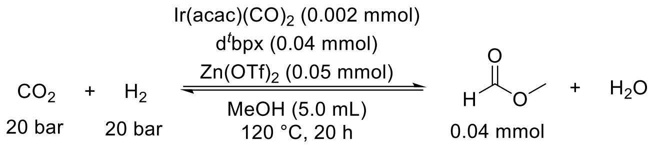

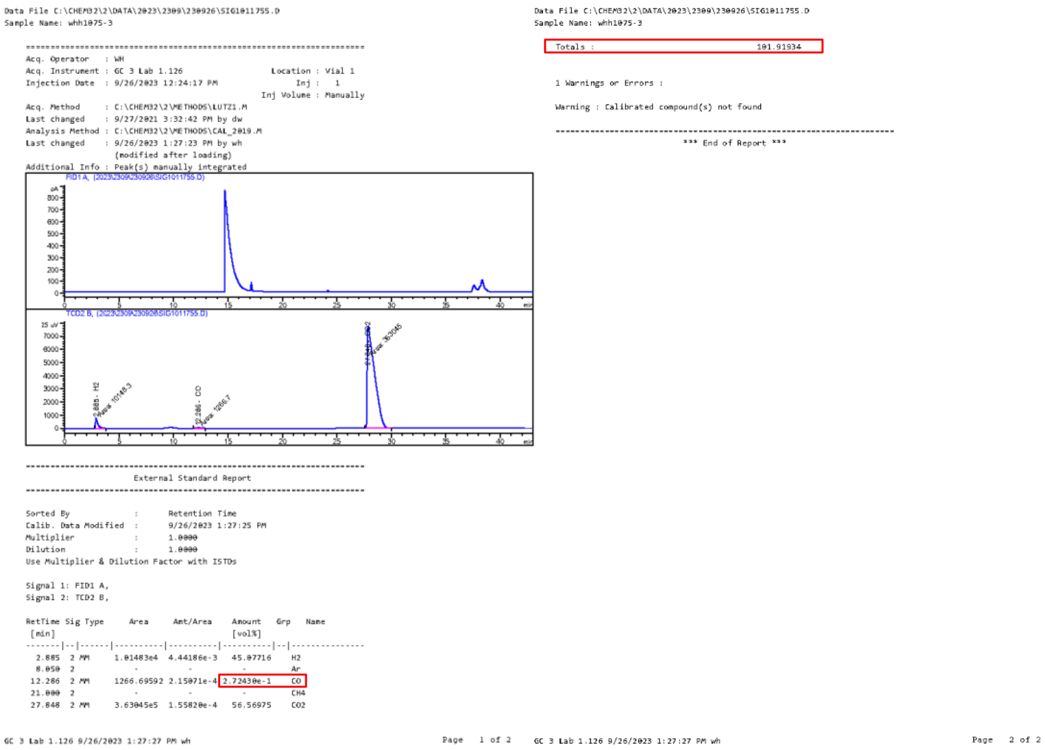

Data File C:\CHEM32\2\DATA\2023\2309\230926\SIG1011755.D
Sample Name: whh1075-3

===
Acq. Operator : WH
Acq. Instrument : GC 3 Lab 1.126 Location : Vial 1
Injection Date : 9/26/2023 12:24:17 PM Inj : 1
Inj Volume : Manually
Acq. Method : C:\CHEM32\2\METHODS\LUTZ1.M
Last changed : 9/27/2021 3:32:42 PM by dw
Analysis Method : C:\CHEM32\2\METHODS\CAL_2019.M
Last changed : 9/26/2023 1:27:23 PM by wh
(modified after loading)
Additional Info : Peak(s) manually integrated

External Standard Report

Sorted By : Retention Time
Calib. Data Modified : 9/26/2023 1:27:25 PM
Multiplier : 1.0000
Dilution : 1.0000
Use Multiplier & Dilution Factor with ISTDs

Signal 1: FID1 A,
Signal 2: TCD2 B,

RetTime [min]	Sig	Type	Area	Amt/Area	Amount [vol%]	Grp	Name
2.885	2	MM	1.01483e4	4.44186e-3	45.07716		H2
8.050	2		-	-	-		Ar
12.286	2	MM	1266.69592	2.15071e-4	2.72430e-1		CO
21.000	2		-	-	-		CH4
27.848	2	MM	3.63045e5	1.55820e-4	56.56975		CO2

GC 3 Lab 1.126 9/26/2023 1:27:27 PM wh Page 1 of 2

Data File C:\CHEM32\2\DATA\2023\2309\230926\SIG1011755.D
Sample Name: whh1075-3

Totals : 101.91934

1 Warnings or Errors :

Warning : Calibrated compound(s) not found

*** End of Report ***

GC 3 Lab 1.126 9/26/2023 1:27:27 PM wh Page 2 of 2

CO concentration = 0.272430 / 101.91934 = 0.267 %.

Fig. S4 | Gas components analysis after a reduction of CO_2 reaction in 25 mL autoclave. Reaction conditions: Ir(acac)$(CO)_2$ (0.002 mmol), d^{t}bpx (0.04 mmol), $Zn(OTf)_2$ (0.05 mmol), MeOH (5.0 mL), CO_2 (20 bar), H_2 (20 bar), 120 °C, 20 h. The yield of methyl formate is determined by ^{1}H NMR using DMSO as internal standard.

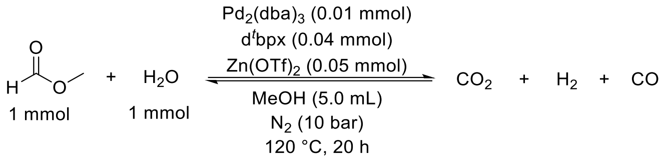

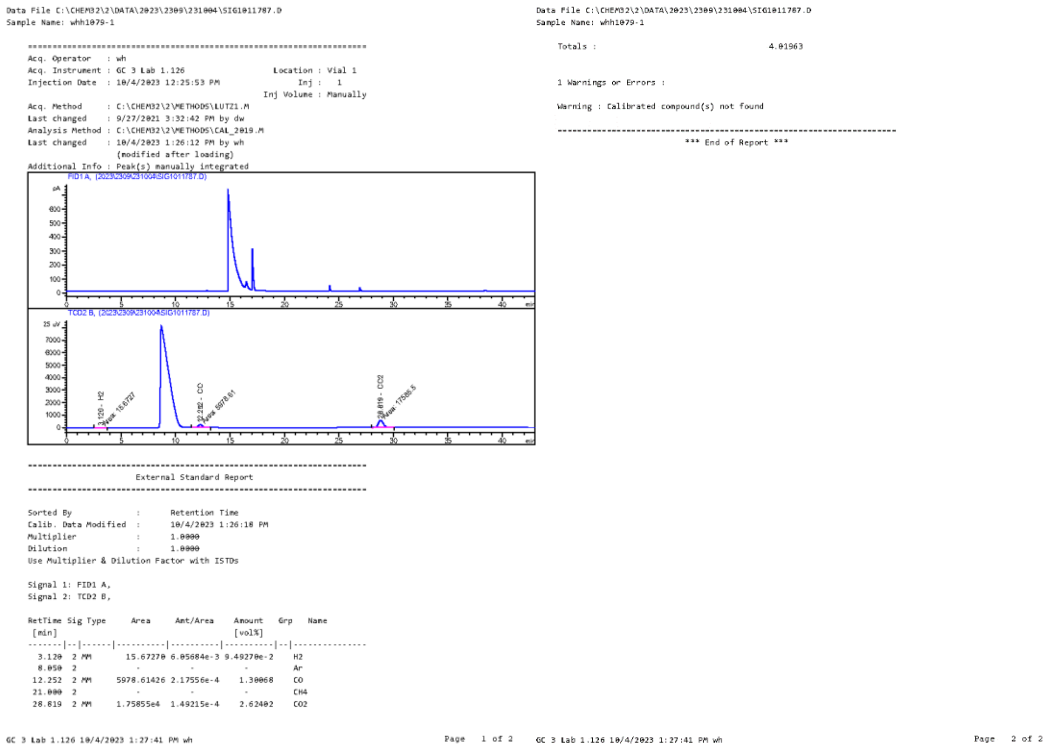

Data File C:\CHEM32\2\DATA\2023\2309\231004\SIG1011787.D
Sample Name: whh1079-1

===
Acq. Operator : wh
Acq. Instrument : GC 3 Lab 1.126 Location : Vial 1
Injection Date : 10/4/2023 12:25:53 PM Inj : 1
Inj Volume : Manually
Acq. Method : C:\CHEM32\2\METHODS\LUTZ1.M
Last changed : 9/27/2021 3:32:42 PM by dw
Analysis Method : C:\CHEM32\2\METHODS\CAL_2019.M
Last changed : 10/4/2023 1:26:12 PM by wh
(modified after loading)
Additional Info : Peak(s) manually integrated

External Standard Report

Sorted By : Retention Time
Calib. Data Modified : 10/4/2023 1:26:18 PM
Multiplier : 1.0000
Dilution : 1.0000
Use Multiplier & Dilution Factor with ISTDs

Signal 1: FID1 A,
Signal 2: TCD2 B,

RetTime [min]	Sig	Type	Area	Amt/Area	Amount [vol%]	Grp	Name
3.120	2	MM	15.67270	6.05684e-3	9.49270e-2		H2
8.050	2		-	-	-		Ar
12.252	2	MM	5978.61426	2.17556e-4	1.30068		CO
21.000	2		-	-	-		CH4
28.819	2	MM	1.75855e4	1.49215e-4	2.62402		CO2

GC 3 Lab 1.126 10/4/2023 1:27:41 PM wh Page 1 of 2

Data File C:\CHEM32\2\DATA\2023\2309\231004\SIG1011787.D
Sample Name: whh1079-1

Totals : 4.01963

1 Warnings or Errors :

Warning : Calibrated compound(s) not found

*** End of Report ***

GC 3 Lab 1.126 10/4/2023 1:27:41 PM wh Page 2 of 2

CO concentration = 1.301 %
H_2 concentration = 0.095% (not precise, calibration limit is 0.1 %)
CO_2 concentration = 2.624 %

Fig. S5 | Gas components analysis after a methylformate decomposition reaction in 25 mL autoclave. Reaction conditions: $Pd_2(dba)_3$ (0.01 mmol), d^{t}bpx (0.04 mmol), $Zn(OTf)_2$ (0.05 mmol), MeOH (5.0 mL), methylformate (1.0 mmol), H_2O (1.0 mmol), N_2 (10 bar), 120 °C, 20 h.

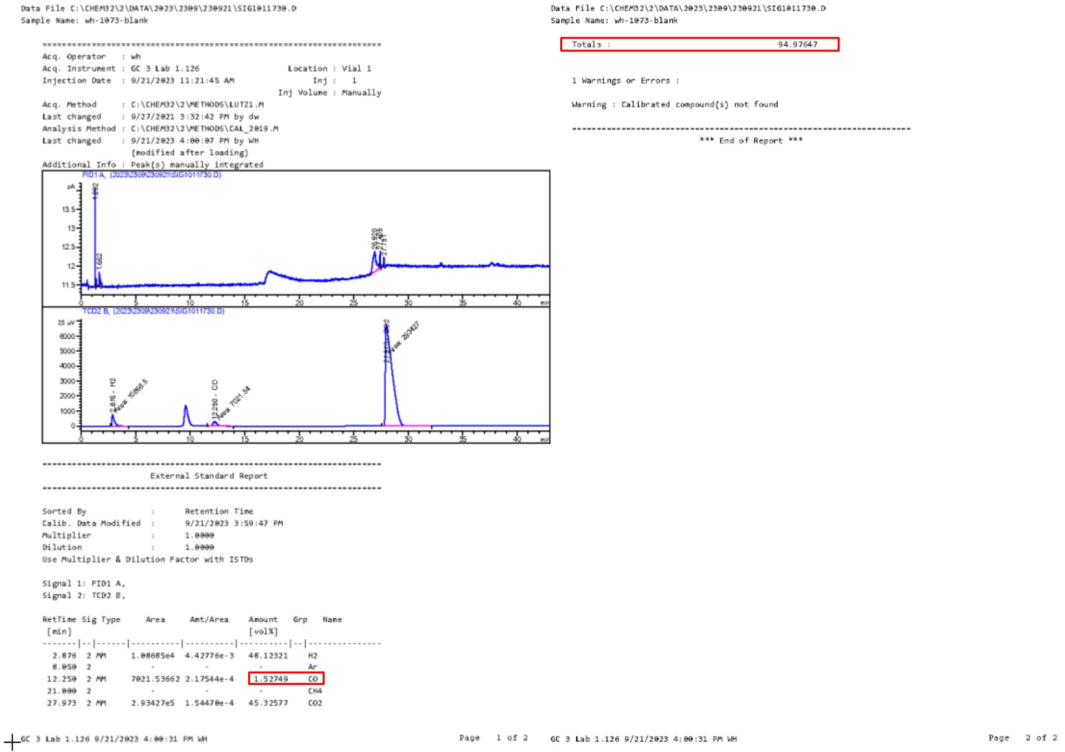

Data File C:\CHEM32\2\DATA\2023\2309\230921\SIG1011730.D
Sample Name: wh-1073-blank

===

Acq. Operator : wh
Acq. Instrument : GC 3 Lab 1.126 Location : Vial 1
Injection Date : 9/21/2023 11:21:45 AM Inj : 1
Inj Volume : Manually
Acq. Method : C:\CHEM32\2\METHODS\LUTZ1.M
Last changed : 9/27/2021 3:32:42 PM by dw
Analysis Method : C:\CHEM32\2\METHODS\CAL_2019.M
Last changed : 9/21/2023 4:00:07 PM by WH
(modified after loading)
Additional Info : Peak(s) manually integrated

External Standard Report

Sorted By : Retention Time
Calib. Data Modified : 9/21/2023 3:59:47 PM
Multiplier : 1.0000
Dilution : 1.0000
Use Multiplier & Dilution Factor with ISTDs

Signal 1: FID1 A,
Signal 2: TCD2 B,

RetTime [min]	Sig	Type	Area	Amt/Area	Amount [vol%]	Grp	Name
2.876	2	MM	1.08685e4	4.42776e-3	48.12321		H2
8.050	2		-	-	-		Ar
12.250	2	MM	7021.53662	2.17544e-4	1.52749		CO
21.000	2		-	-	-		CH4
27.973	2	MM	2.93427e5	1.54470e-4	45.32577		CO2

GC 3 Lab 1.126 9/21/2023 4:00:31 PM WH Page 1 of 2

Data File C:\CHEM32\2\DATA\2023\2309\230921\SIG1011730.D
Sample Name: wh-1073-blank

Totals : 94.97647

1 Warnings or Errors :

Warning : Calibrated compound(s) not found

*** End of Report ***

GC 3 Lab 1.126 9/21/2023 4:00:31 PM WH Page 2 of 2

CO concentration = 1.52749 / 94.97647 = 1.608 %.

Fig. S6 | Applied carbon dioxide and hydrogen gas with low concentration of CO.

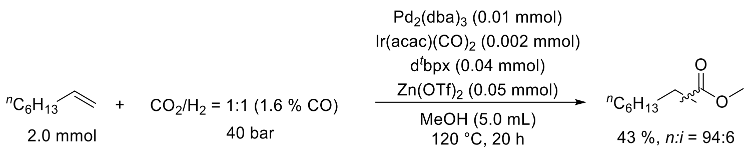

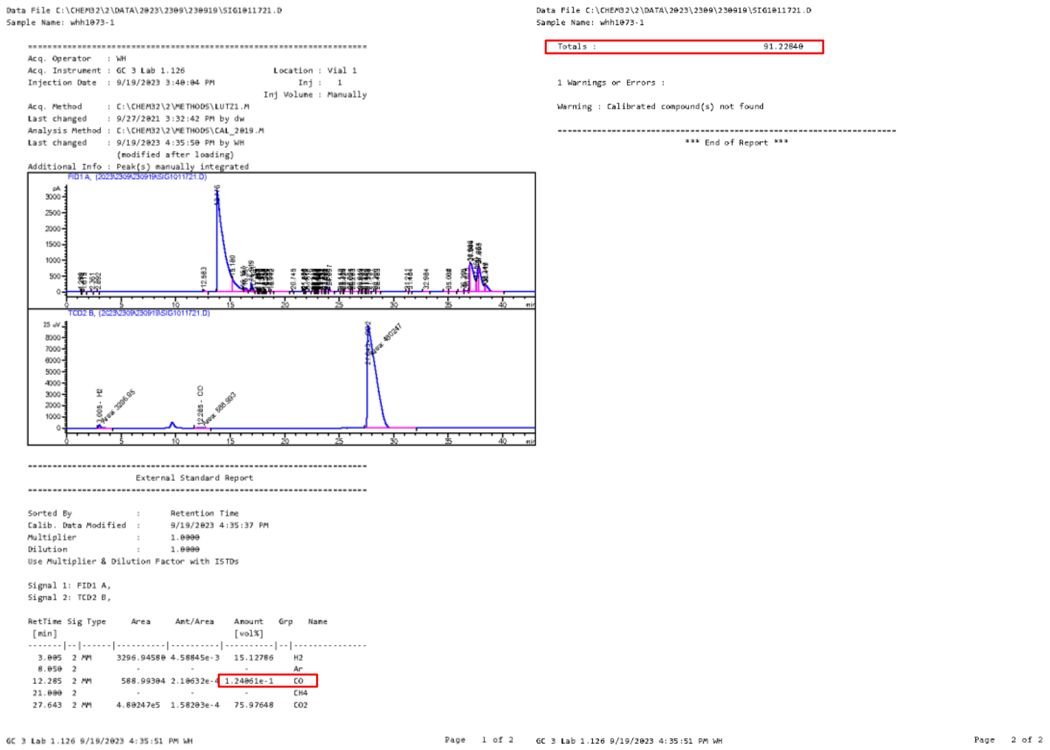
Data File C:\CHEM32\2\DATA\2023\2309\230919\SIG1011721.D
Sample Name: whh1073-1

Acq. Operator : WH
Acq. Instrument : GC 3 Lab 1.126 Location : Vial 1
Injection Date : 9/19/2023 3:40:04 PM Inj : 1
Inj Volume : Manually
Acq. Method : C:\CHEM32\2\METHODS\LUTZ1.M
Last changed : 9/27/2021 3:32:42 PM by dw
Analysis Method : C:\CHEM32\2\METHODS\CAL_2019.M
Last changed : 9/19/2023 4:35:50 PM by WH
(modified after loading)
Additional Info : Peak(s) manually integrated

External Standard Report

Sorted By : Retention Time
Calib. Data Modified : 9/19/2023 4:35:37 PM
Multiplier : 1.0000
Dilution : 1.0000
Use Multiplier & Dilution Factor with ISTDs

Signal 1: FID1 A,
Signal 2: TCD2 B,

RetTime [min]	Sig	Type	Area	Amt/Area	Amount [vol%]	Grp	Name
3.005	2	MM	3296.94580	4.58845e-3	15.12786		H2
8.050	2		-	-	-		Ar
12.285	2	MM	588.99304	2.10632e-4	1.24061e-1		CO
21.000	2		-	-	-		CH4
27.643	2	MM	4.80247e5	1.58203e-4	75.97648		CO2

GC 3 Lab 1.126 9/19/2023 4:35:51 PM WH Page 1 of 2

Data File C:\CHEM32\2\DATA\2023\2309\230919\SIG1011721.D
Sample Name: whh1073-1

Totals : 91.22840

1 Warnings or Errors :

Warning : Calibrated compound(s) not found

*** End of Report ***

GC 3 Lab 1.126 9/19/2023 4:35:51 PM WH Page 2 of 2

CO concentration = 0.12406 / 91.2264 = 0.136 %.

Fig. S7 | Gas components analysis after reaction using 25 mL autoclave. Reaction conditions: 1-octene (2.0 mmol), $Pd_2(dba)_3$ (0.01 mmol), $Ir(acac)(CO)_2$ (0.002 mmol), d^{t}bpx (0.04 mmol), $Zn(OTf)_2$ (0.05 mmol), MeOH (5.0 mL), CO_2/H_2 = 1:1 (40 bar with 1.6 % CO), 120 °C, 20 h.

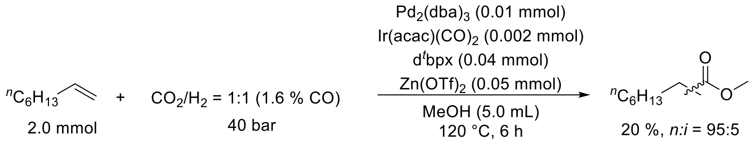

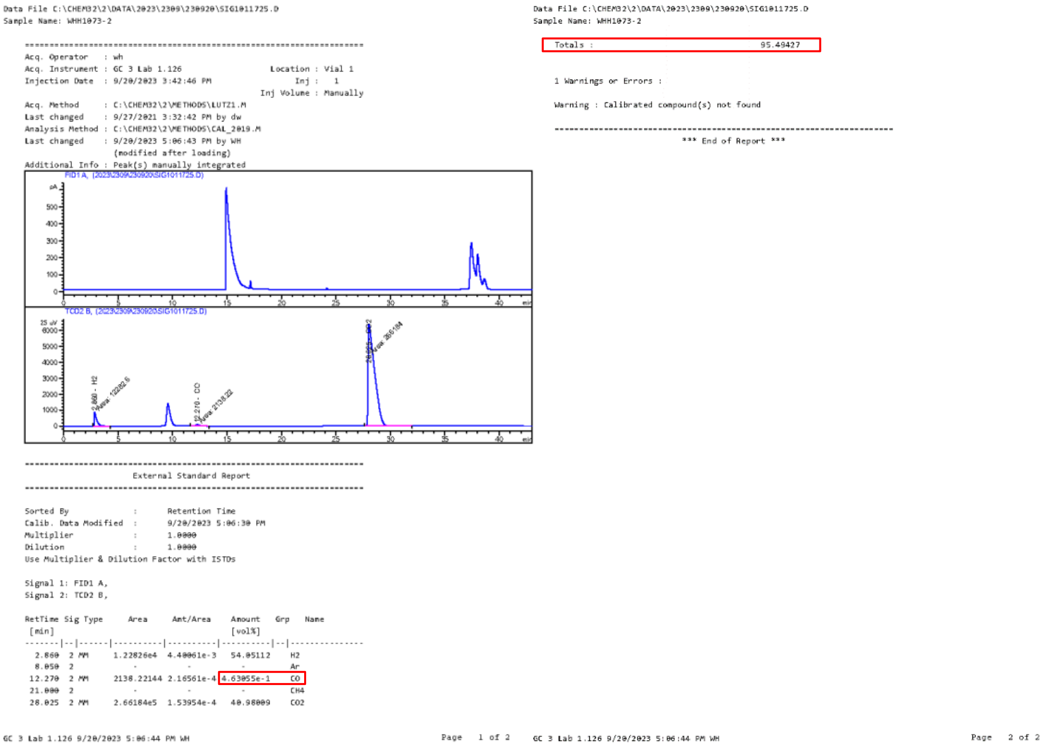

```
Data File C:\CHEM32\2\DATA\2023\2309\230920\SIG1011725.D
Sample Name: WHH1073-2

=====================================================================
Acq. Operator   : wh
Acq. Instrument : GC 3 Lab 1.126                 Location : Vial 1
Injection Date  : 9/20/2023 3:42:46 PM                Inj :    1
                                               Inj Volume : Manually
Acq. Method     : C:\CHEM32\2\METHODS\LUTZ1.M
Last changed    : 9/27/2021 3:32:42 PM by dw
Analysis Method : C:\CHEM32\2\METHODS\CAL_2019.M
Last changed    : 9/20/2023 5:06:43 PM by WH
                  (modified after loading)
Additional Info : Peak(s) manually integrated
```



```
=====================================================================
                      External Standard Report
=====================================================================

Sorted By             :      Retention Time
Calib. Data Modified  :      9/20/2023 5:06:30 PM
Multiplier            :      1.0000
Dilution              :      1.0000
Use Multiplier & Dilution Factor with ISTDs

Signal 1: FID1 A,
Signal 2: TCD2 B,
```

RetTime [min]	Sig	Type	Area	Amt/Area	Amount [vol%]	Grp	Name
2.860	2	MM	1.22826e4	4.40061e-3	54.05112		H2
8.050	2		-	-	-		Ar
12.270	2	MM	2138.22144	2.16561e-4	4.63055e-1		CO
21.000	2		-	-	-		CH4
28.025	2	MM	2.66184e5	1.53954e-4	40.98009		CO2

GC 3 Lab 1.126 9/20/2023 5:06:44 PM WH Page 1 of 2

```
Data File C:\CHEM32\2\DATA\2023\2309\230920\SIG1011725.D
Sample Name: WHH1073-2

  Totals :                                 95.49427

  1 Warnings or Errors :

  Warning : Calibrated compound(s) not found

=====================================================================
                        *** End of Report ***
```

GC 3 Lab 1.126 9/20/2023 5:06:44 PM WH Page 2 of 2

CO concentration = 0.463055 / 95.49427 = 0.485 %.

Fig. S8 | Gas components analysis after reaction using 25 mL autoclave. Reaction conditions: 1-octene (2.0 mmol), $Pd_2(dba)_3$ (0.01 mmol), $Ir(acac)(CO)_2$ (0.002 mmol), d^{t}bpx (0.04 mmol), $Zn(OTf)_2$ (0.05 mmol), MeOH (5.0 mL), CO_2/H_2 = 1:1 (40 bar with 1.6 % CO), 120 °C, 6 h.

6. Preliminary reaction pathway investigation

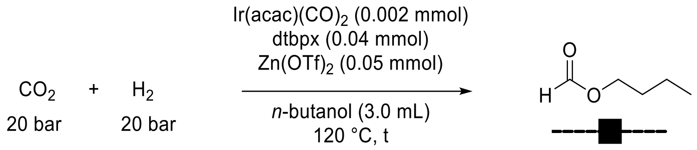

Entry	t (h)	***n*-butyl formate (mmol)**
**1**	10	0.068
**2**	20	0.077
**3**	30	0.088
**4**	40	0.102
**5**	50	0.115
**6**	60	0.125

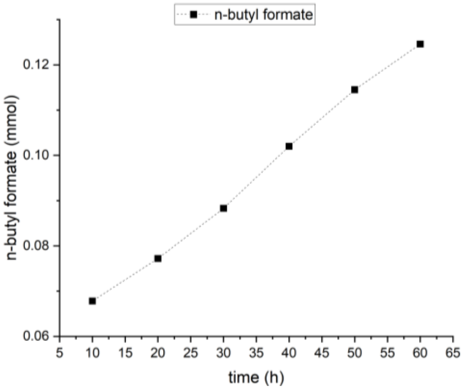

Fig. S9 | Mechanism study: CO_2 reduction in *n*-butanol. Reaction conditions: Ir(acac)$(CO)_2$ (0.002 mmol), d^tbpx (0.05 mmol), $Zn(OTf)_2$ (0.04 mmol), *n*-butanol (3 mL), CO_2 (20 bar), H_2 (20 bar), 120 °C. The yield is determined by GC with THF internal standard.

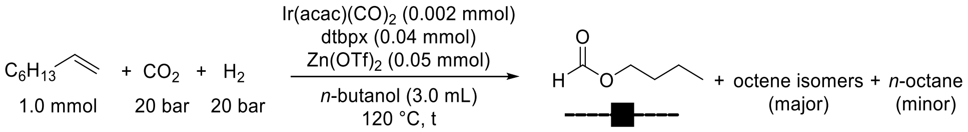

Entry	t (h)	***n*-butyl formate (mmol)**
**1**	10	0.066
**2**	20	0.078
**3**	30	0.088
**4**	40	0.101
**5**	50	0.116
**6**	60	0.125

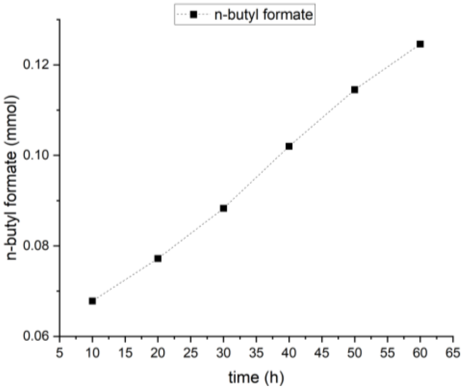

Fig. S10 | Mechanism study: Alkoxycarbonylation of 1-octene in *n*-butanol using only Ir. Reaction conditions: 1-octene (1.0 mmol), Ir(acac)$(CO)_2$ (0.002 mmol), d^tbpx (0.05 mmol), $Zn(OTf)_2$ (0.04 mmol), *n*-butanol (3 mL), CO_2 (20 bar), H_2 (20 bar), 120 °C. The yield is determined by GC with THF internal standard.

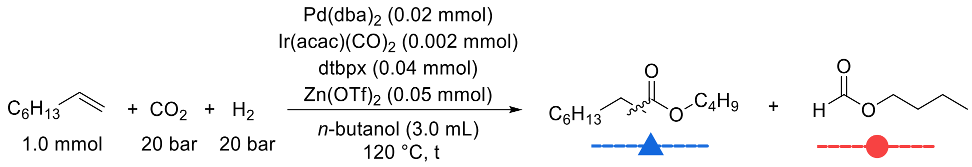

Entry	t (h)	Butyl nonanoates (mmol)	n-butyl formate (mmol)
**1**	10	0.038	0.062
**2**	20	0.101	0.063
**3**	30	0.158	0.058
**4**	40	0.194	0.053
**5**	50	0.252	0.060
**6**	60	0.296	0.055

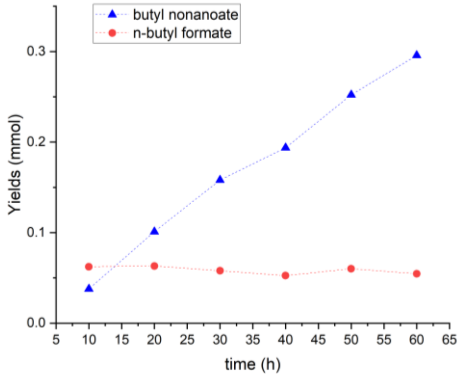

Fig. S11 | Mechanism study: Alkoxycarbonylation of 1-octene in *n*-butanol using Pd-Ir. Reaction conditions: 1-octene (1.0 mmol), Pd(dba)$_2$ (0.02 mmol), Ir(acac)(CO)$_2$ (0.002 mmol), d^{t}bpx (0.04 mmol), Zn(OTf)$_2$ (0.05 mmol), *n*-butanol (3 mL), CO_2 (20 bar), H_2 (20 bar), 120 °C. The yield is determined by GC with THF internal standard.

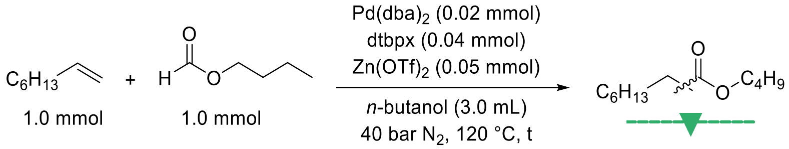

Entry	t (h)	Butyl nonanoates (mmol)
**1**	10	0.066
**2**	20	0.116
**3**	30	0.145
**4**	40	0.188
**5**	50	0.211
**6**	60	0.222

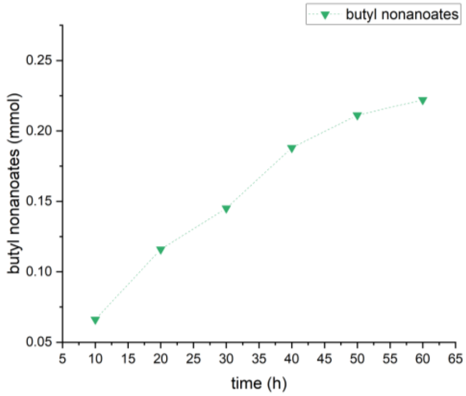

Fig. S12 | Mechanism study: Alkoxycarbonylation of 1-octene with *n*-butyl formate. Reaction conditions: 1-octene (1.0 mmol), *n*-butyl formate (1.0 mmol), Pd(dba)$_2$ (0.02 mmol), d^{t}bpx (0.04 mmol), Zn(OTf)$_2$ (0.05 mmol), *n*-butanol (3 mL), N_2 (40 bar), 120 °C. The yield is determined by GC with THF internal standard.

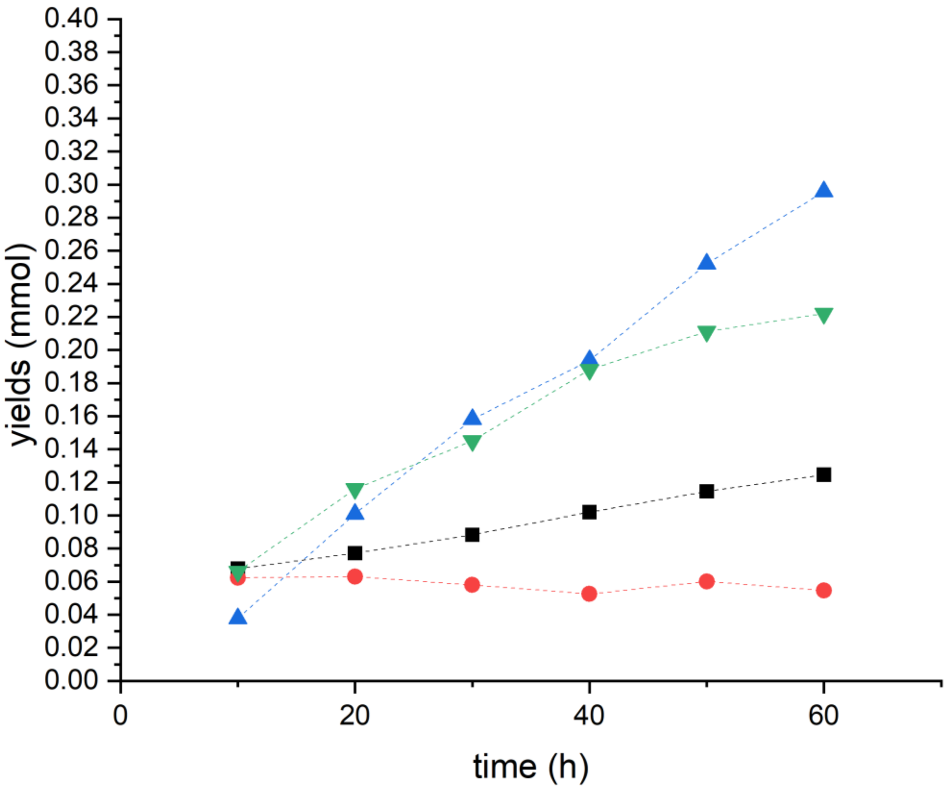

Fig. S13 | Mechanism study: Black square: CO_2 reduction in *n*-butanol (Fig. S9), Alkoxycarbonylation of 1-octene in *n*-butanol using only Ir (Fig. S10); Blue up triangle and red circle: Alkoxycarbonylation of 1-octene in *n*-butanol using Pd-Ir (Fig. S11); Green down triangle: Alkoxycarbonylation of 1-octene with *n*-butyl formate (Fig. S12).

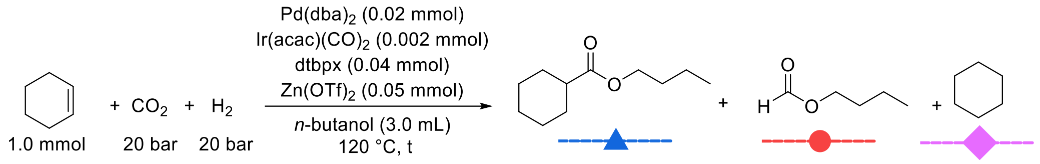

Entry	t (h)	Butyl cyclohexanecarboxylate (mmol)	n-butyl formate (mmol)	Cyclohexane (mmol)
**1**	10	0.003	0.058	0.104
**2**	20	0.008	0.081	0.102
**3**	30	0.017	0.081	0.111
**4**	40	0.024	0.086	0.130
**5**	50	0.029	0.085	0.145
**6**	60	0.036	0.086	0.152

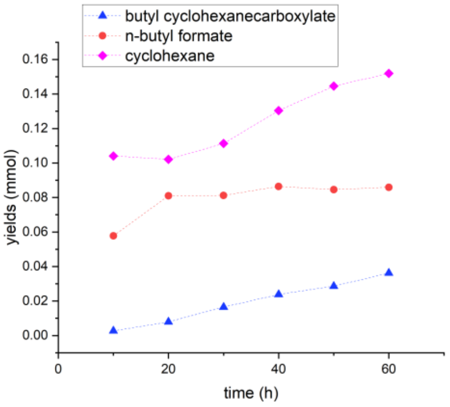

Fig. S14 | Mechanism study: Alkoxycarbonylation of cyclohexene in *n*-butanol. Reaction conditions: cyclohexene (1.0 mmol), Pd(dba)$_2$ (0.02 mmol), Ir(acac)(CO)$_2$ (0.002 mmol), d^{t}bpx (0.04 mmol), Zn(OTf)$_2$ (0.05 mmol), *n*-butanol (3 mL), CO_2 (20 bar), H_2 (20 bar), 120 °C. The yield is determined by GC with THF internal standard.

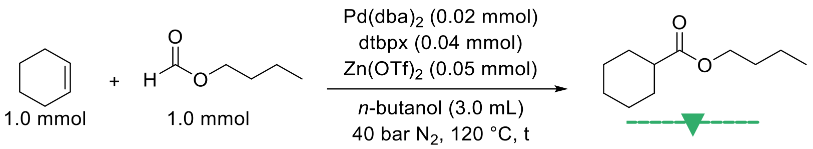

Entry	t (h)	Butyl cyclohexanecarboxylate (mmol)
**1**	10	0.006
**2**	20	0.012
**3**	30	0.015
**4**	40	0.019
**5**	50	0.025
**6**	60	0.038

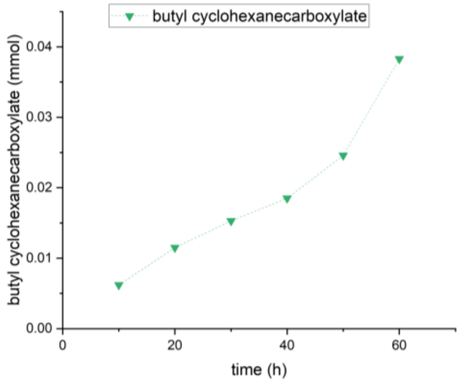

Fig. S15 | Mechanism study: Alkoxycarbonylation of cyclohexene with *n*-butyl formate. Reaction conditions: cyclohexene (1.0 mmol), *n*-butyl formate (1.0 mmol), Pd(dba)$_2$ (0.02 mmol), d^{t}bpx (0.04 mmol), Zn(OTf)$_2$ (0.05 mmol), *n*-butanol (3 mL), N_2 (40 bar), 120 °C. The yield is determined by GC with THF internal standard.

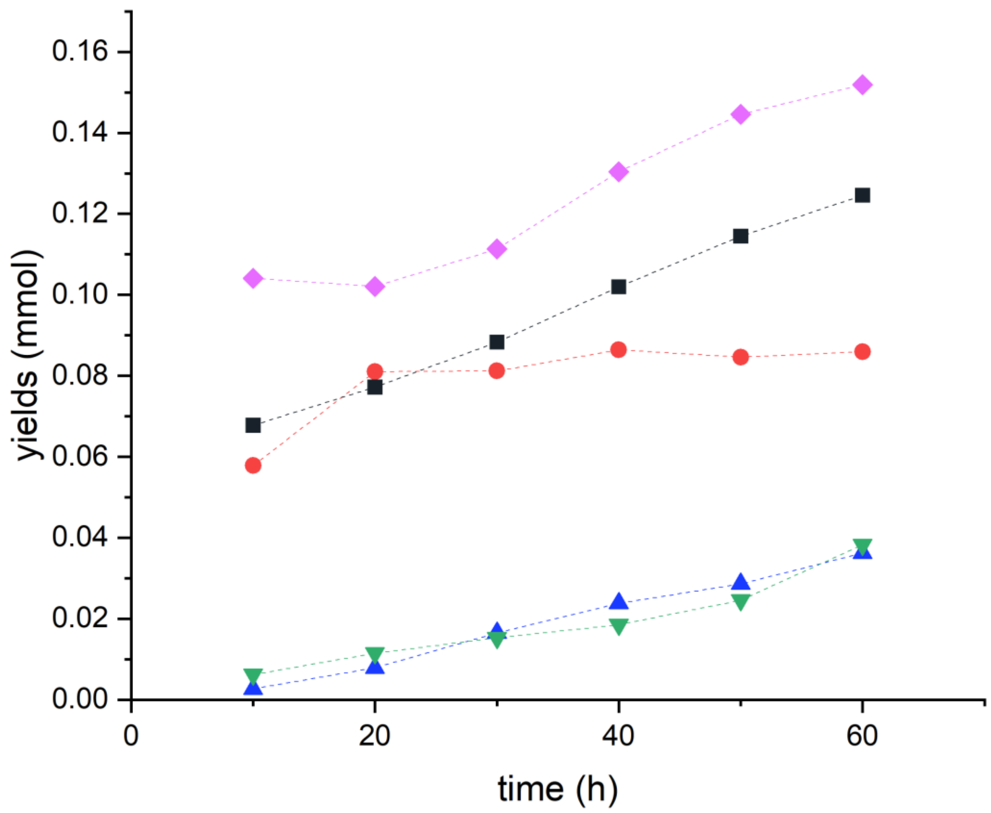

Fig. S16 | Mechanism study: Black square: CO_2 reduction in *n*-butanol (Fig. S9); Blue up triangle and red circle: Alkoxycarbonylation of cyclohexene in *n*-butanol (Fig. S14); Green down triangle: Alkoxycarbonylation of cyclohexene with *n*-butyl formate (Fig. S15).

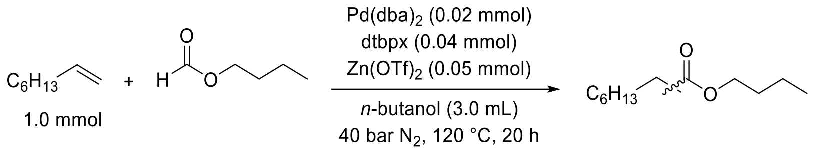

Entry	initial n-butyl formate (mmol)	*n*-butyl formate (mmol)
**1**	0.2	0.075
**2**	0.5	0.095
**3**	1.0	0.116
**4**	1.5	0.136
**5**	2.0	0.171
**6**	4.0	0.138
**7**	6.0	0.103
**8**[a]	26	<0.01

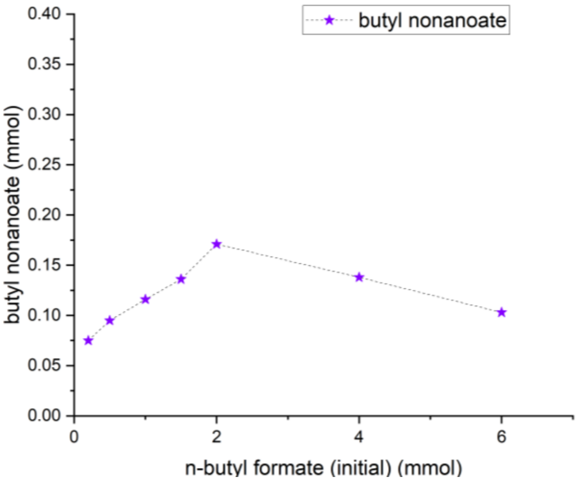

Fig. S17 | Mechanism study: Alkoxycarbonylation of 1-octene with different initial amount of *n*-butyl formate. Reaction conditions: 1-octene (1.0 mmol), $Pd(dba)_2$ (0.02 mmol), d^{t}bpx (0.04 mmol), $Zn(OTf)_2$ (0.05 mmol), *n*-butanol (3 mL), N_2 (40 bar), 120 °C. The yield is determined by GC with THF internal standard. a) *n*-butyl formate solution (3 mL).

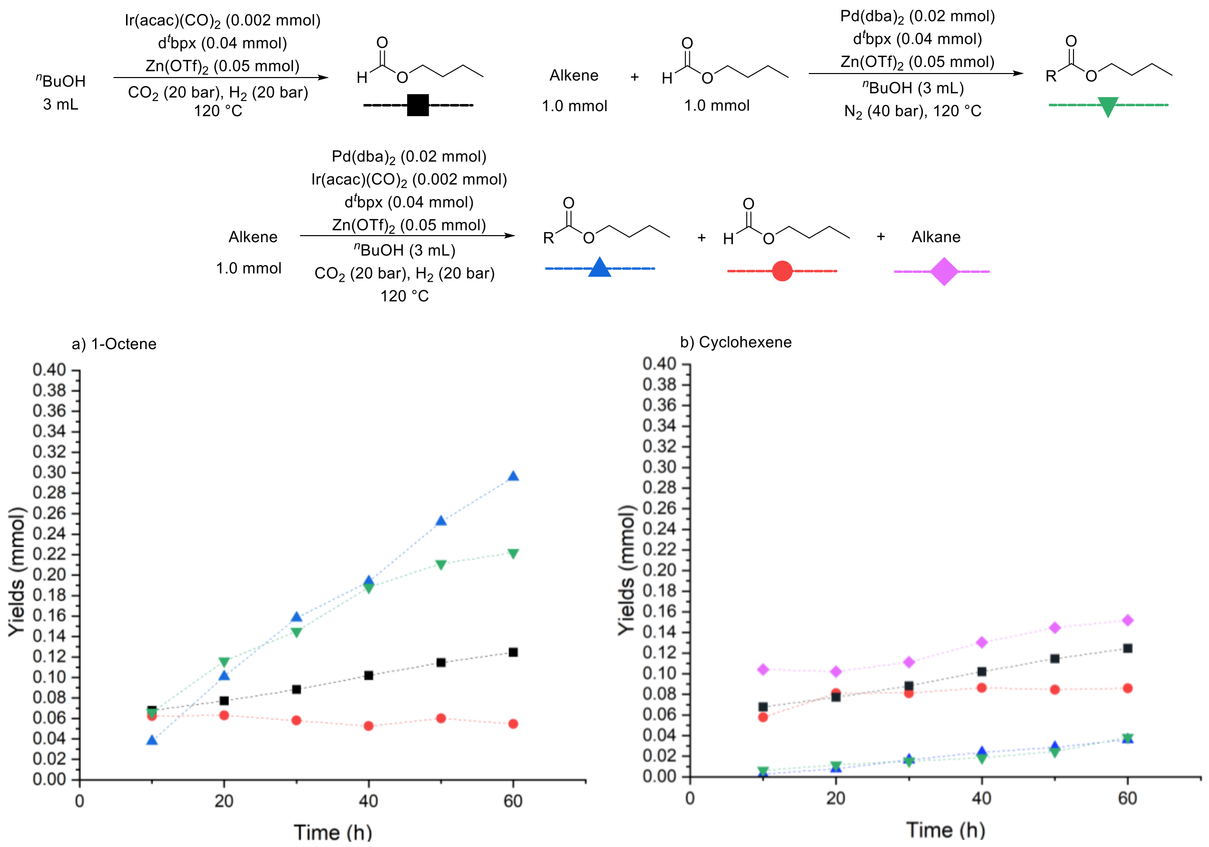

Fig. S18 | Mechanism study: comparing kinetic study profiles of 1-octene and cyclohexene.

7. Theoretical reaction pathway investigation

Mechanism discussion

Firstly, the coordination ability of Ir and Pd metal centers towards Lucite (d^{t}bpx) ligand was compared (Figure S1). For the substitution of $HIr(CO)_4$ with Lucite ligand, there are two structural isomers (**EA**, equatorial-axial substitution, and **EE**, equatorial-equatorial substitution) in close energy (-3.30 and -4.05 kcal/mol, respectively). On the contrary, the substitution of $[HPd(CO)_3]^+$ with Lucite ligand is exothermic by 39.82 kcal/mol, much higher than that for $HIr(CO)_4$. The ligand exchange process of $HIr(CO)_2$(d^{t}bpx) and $[HPd(CO)_3]^+$ is highly exergonic by 36.52 kcal/mol. Therefore, Lucite ligand prefers coordination to the Pt center instead of the Ir center, and the corresponding Ir complex becomes only possible with excess of ligand.

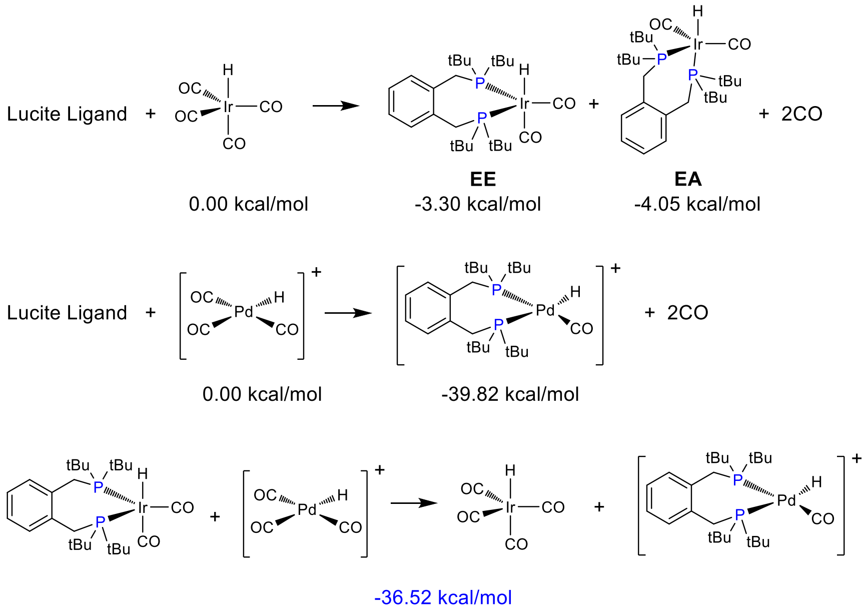

Fig. S19 | Substitution Gibbs free energy (Δ*G*, kcal/mol) of Ir and Pd complexes with Lucite ligand.

Due to the weak coordination of Lucite ligand with the Ir center, we firstly used $HIr(CO)_4$ complex as a model complex to study the reverse water gas shift (RWGS) process as well as the effect of $Zn(OTf)_2$ as a Lewis acid additive (Fig. S20). Since HCOOH can be generated as an intermediate, we considered the pathway for producing HCOOH.

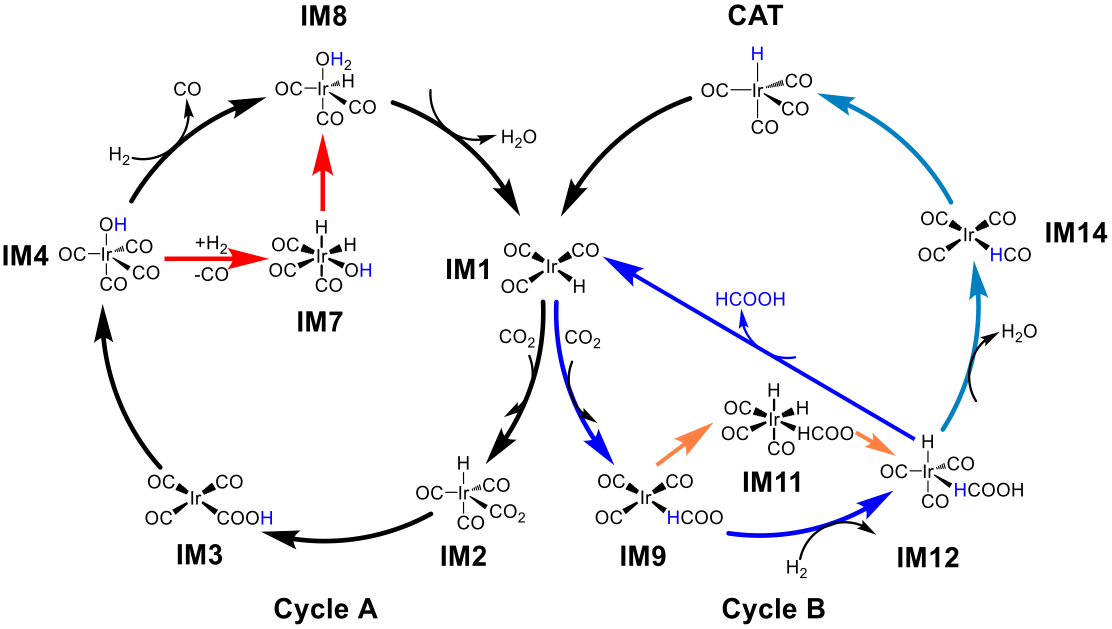

Fig. S20 | Proposed RWGS catalytic cycle catalyzed by $HIr(CO)_4$.
(**CAT** represents the corresponding catalyst, **IM** represents the intermediate during the reaction.)

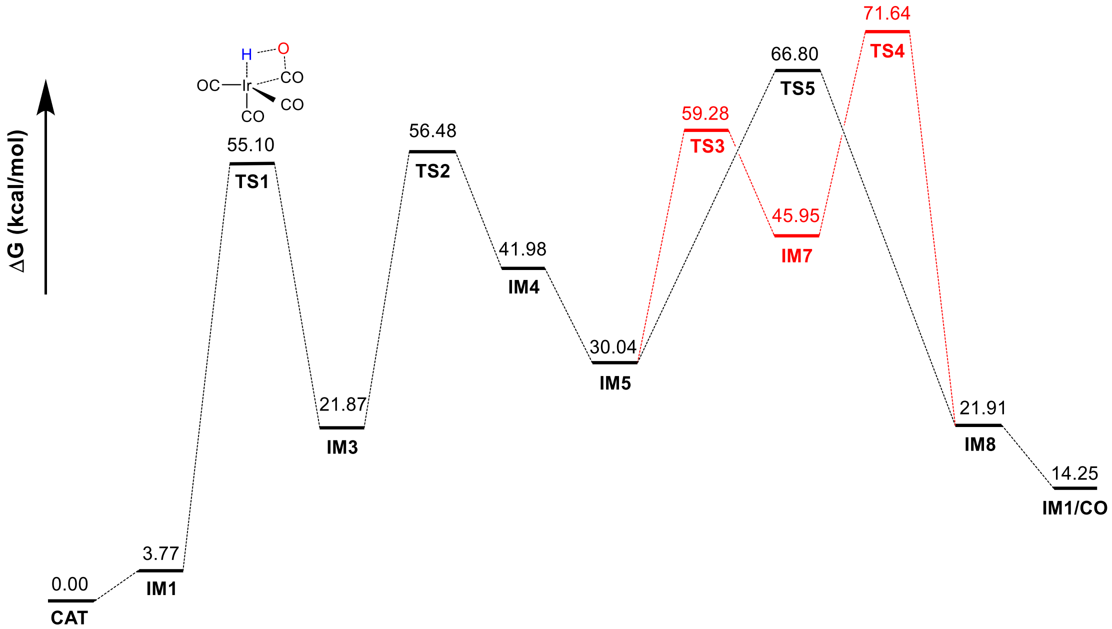

Fig. S21 | Gibbs free energy profile of the catalytic Cycle A shown in Scheme S1 catalyzed by $HIr(CO)_4$.
(**CAT** represents the corresponding catalyst, **IM** represents the intermediate during the reaction, and **TS** represents the transition state.)

The catalytic cycle starts with the CO de-coordination of the pre-catalysts (**CAT**, $HIr(CO)_4$), called as the initiation step in general leading to the **IM1** complex ($HIr(CO)_3$), and this is endergonic by 3.77 kcal/mol. Next, gas phase CO_2 molecule interacts with the H(-Ir) to form either (Ir-)COOH or (Ir-)HCOO group, resulting in **IM3** or **IM9** complex, respectively, via high apparent barrier of 55.10 kcal/mol for **TS1** or 42.06 kcal/mol for **TS6**. Furthermore, the (Ir-)COOH group formation in endergonic by 18.10 kcal/mol, while that of the (Ir-)HCOO group is endergonic by 10.99 kcal/mol. We define the catalytic cycle associated with (Ir-)COOH group and (Ir-)HCOO group as **Cycle A** and **Cycle B**, respectively. The obtained Gibbs free energy profile of **Cycle A** and **Cycle B** is shown in Fig. S2 and Fig. S3, respectively. It should be mentioned that the CO_2 coordination with the Ir center (**IM2**) through O atom ($d_{\text{Ir-O}}$ = 2.50 Å) is endergonic with apparent energy of 19.25, and much high than the physically interacted $HIr(CO)_3$ and CO_2 structure. The latter one is spatially more favorable to form transition state structures. Therefore, the energy of **IM2** is not shown on the Gibbs free energy profile. The coordination of H_2 and H_2O is also not included in the Gibbs free energy profile for the same reasons.

In **Cycle A**, the (Ir-)COOH group undergoes decomposition into CO(-Ir-)OH species (**IM4**), this step need to overcome a barrier of 34.62 kcal/mol (**TS2**), and is endergonic by 20.11 kcal/mol. **IM4** further deduces one CO molecule to form **IM5** complex, this process is endergonic by 11.93 kcal/mol. The **IM5** complex subsequently undergoes H_2 oxidative addition reaction to form **IM7** complex, and this step is endergonic by 15.90 kcal/mol with a barrier of 29.23 kcal/mol (**TS3**). **IM7** undergoes dehydration process and goes back to **IM1** complex closing the catalytic cycle, this step is highly endergonic by 31.70 kcal/mol and has high barrier of 25.69 kcal/mol (**TS3**). The corresponding apparent barrier is as high as 71.64 kal/mol. Although H_2 oxidative addition and

dehydration can perform directly as one hydrolysis step, i.e. through **IM5 → TS5 → IM8/IM1** process, the apparent Gibbs free energy barrier (66.80 kal/mol) is also considerably high.

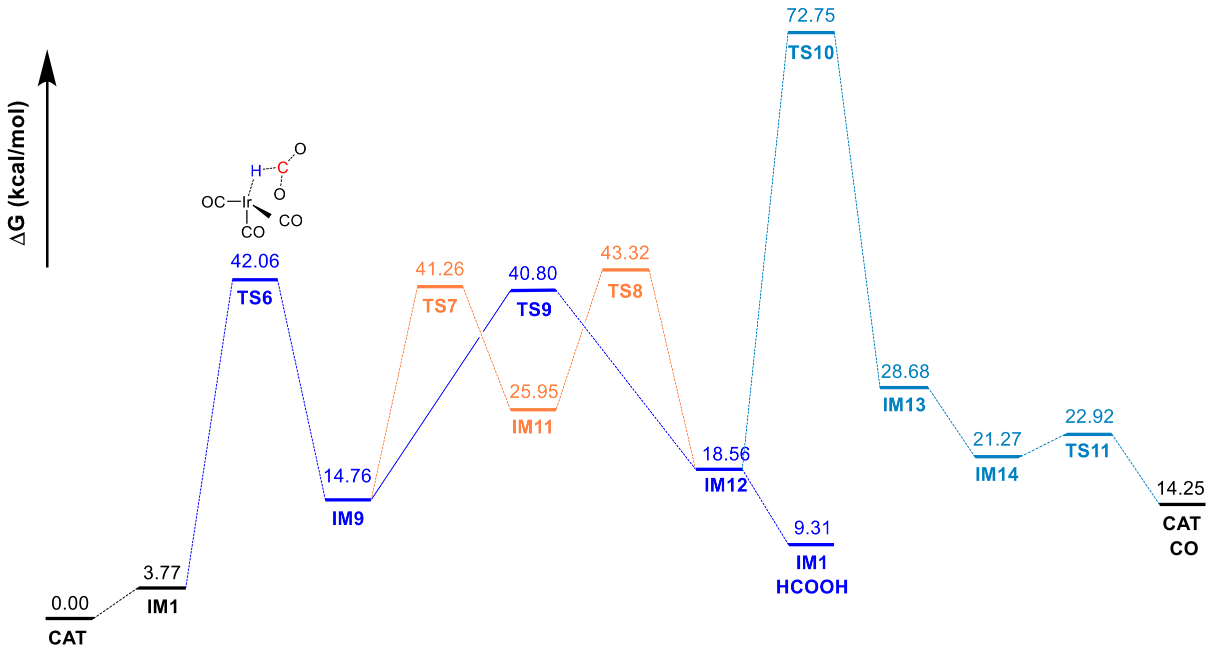

Fig. S22 | Gibbs free energy profile of the catalytic Cycle B shown in Scheme S1 catalyzed by HIr(CO)$_4$.

For the (Ir-)HCOO group pathway (**Cycle B**), **IM9** complex can also perform H_2 oxidative addition reaction and forms **IM11** complex, and this step is endergonic by 11.20 kcal/mol with a barrier of 26.50 kcal/mol (**TS7**). Then one of the Ir-H hydrides transfer to the (Ir-)HCOO to form coordinated HCOOH species (**IM12**), and this process is exergonic by 7.40 kcal/mol with a barrier of 17.37 kcal/mol (**TS8**). Similarly, the H_2 oxidative addition and hydrogen transfer process can perform directly as one hydrolysis step through **IM9 → TS9 → IM12** pathway, and the barrier of this step is 26.04 kcal/mol. Furthermore, the apparent barrier of hydrolysis step is slightly lower than the combination step of oxidative addition and hydrogen transfer (40.80 vs. 43.32 kcal/mol). The new formed (Ir-)HCOOH then can transform into (Ir-)HCO and deduce one H_2O molecule by interacting with another Ir-H hydride (**IM14**) through **TS10**, and the barrier of this step is 54.19 kcal/mol. The apparent barrier is as high as 72.75 kcal/mol.

Based on the above results, one can see that all dehydration steps have considerably high apparent barriers (over 66.80 kcal/mol). However, CO_2 transforming into HCOOH through CO_2→ HCOO(**TS6**) → HCOOH (**TS9** by hydrolysis) pathway is more dynamically favorable, and the apparent Gibbs free energy barrier is 42.06 kcal/mol.
The desorption of HCOOH is exergonic by 9.25 kcal/mol. Importantly, the whole reaction for CO_2 and H_2 transfer into HCOOH is thermodynamically more favorable than the process of RWGS (9.31 vs. 14.25 kcal/mol). Therefore, one can conclude that in our Ir-catalyzed CO_2 transformation system, the HCOOH is easier to produce than CO and H_2O, and the reaction will stop at HCOOH formation step.

The effect of $Zn(OTf)_2$ additive

Although the formation of HCOOH is favorable than the RWGS process for $HIr(CO)_4$ catalyst, the apparent barrier (42.06 kcal/mol) is also too high to overcome under current reaction temperature (393 K). Experiment results have found that the addition of $Zn(OTf)_2$ additive can effectively promote HCOOH production. Therefore, we studied the effect of $Zn(OTf)_2$ on the above mentioned most favorable pathway.

Before conducting the mechanism study, we first compared the ion exchange possibility between $Zn(OTf)_2$ and methanol (HOMe). Because methanol serves as both solvent and important source of metal-H species in the reaction, which can be obtained through the ion exchange process. As shown in Fig. S4, the substitution of one OTf anion by one OMe anion is endergonic by 13.51 kcal/mol, and the substitution of two OTf anions by two OMe anions is endergonic by 28.78 kcal/mol. According to the equilibrium constants, the relative molar ratio of $Zn(OTf)_2$, Zn(OTf)(OMe) and $Zn(OMe)_2$ species is 99.88: 0.12 : 0. Therefore, $Zn(OTf)_2$ should be the most dominant species, and only trace amounts of Zn(OTf)(OMe) species should be present. For comparison, nevertheless, all these three species effect on HCOOH and RWGS processes catalyzed by $HIr(CO)_4$ were studied as following.

$$\underset{0.00\ \text{kcal/mol}}{Zn(OTf)_2} \underset{\text{-HOTf}}{\overset{\text{+HOMe}}{\rightleftharpoons}} \underset{13.51\ \text{kcal/mol}}{Zn(OTf)(OMe)} \underset{\text{-HOTf}}{\overset{\text{+HOMe}}{\rightleftharpoons}} \underset{28.78\ \text{kcal/mol}}{Zn(OMe)_2}$$

Fig. S23 | Equilibrium equation of ion exchange between $Zn(OTf)_2$ and HOMe.

The Gibbs free energy profile of catalytic cycle assisted by $Zn(OTf)_2$ related species is shown in Fig. S5. It shows clearly that with the help of $Zn(OTf)_2$, the apparent Gibbs free energy barrier of **TS6** which leading to the (Ir-)HCOO group formation is reduced from 42.06 kcal/mol to 30.17 kcal/mol. Assisted by Zn(OTf)(OMe) and $Zn(OMe)_2$, the apparent Gibbs free energy barrier of **TS6** is also reduced to 32.61 and 37.54 kcal/mol, respectively. Likewise, the apparent Gibbs free energy barrier of **TS9** related to the hydrolysis step of HCOOH formation is reduced to 31.12, 33.20, 39.11 kcal/mol for $Zn(OTf)_2$, Zn(OTf)(OMe) and $Zn(OMe)_2$ species, respectively, compared to that (40.80 kcal/mol) without additive. Clearly, the zinc Lewis acid greatly helped the HCOOH formation.

The effect of additive ($Zn(OTf)_2$ and Zn(OTf)(OMe)) on the HCOOH dehydration part were also studied. As shown in Fig. S5, the corresponding apparent Gibbs free energy barrier of **TS10** increases from 72.75 kcal/mol to 74.29/73.33 kcal/mol instead. The results demonstrate that, the Zinc Lewis acid additive can only facilitate the HCOOH formation by lower the Gibbs free energy barriers of initial hydrogenation and subsequent hydrolysis steps, but slightly hinder the dehydration step which leading the CO formation.

In summary, the addition of Zinc Lewis acid reduces the apparent barrier from 42.06 kal/mol (**TS6**) to 31.12 kcal/mol (**TS9**) by about 11 kcal/mol, which will greatly reduce the reaction temperature so that the transformation of CO_2 to HCOOH can be achieved at current temperature.

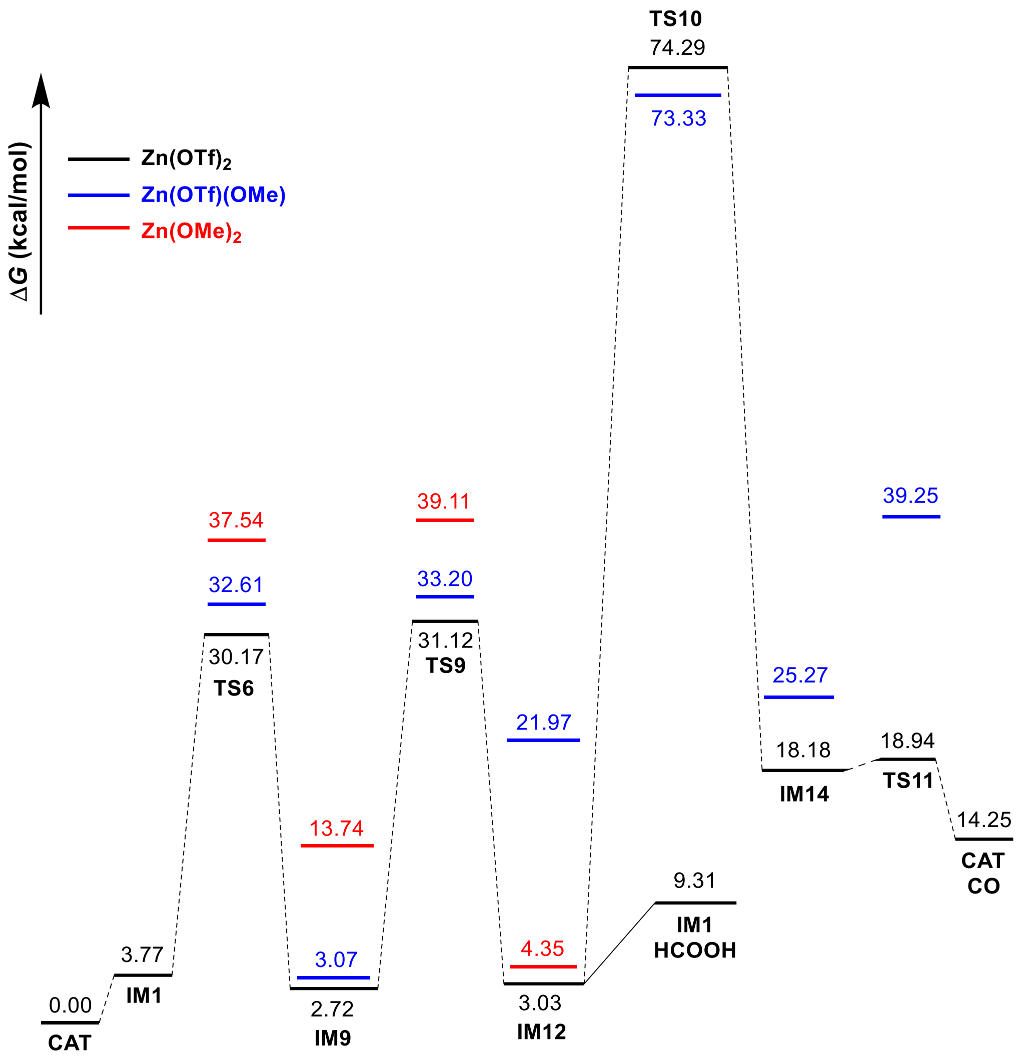

Fig. S24 | The Gibs free energy profile of catalytic cycle assisted by $Zn(OTf)_2$ related species.

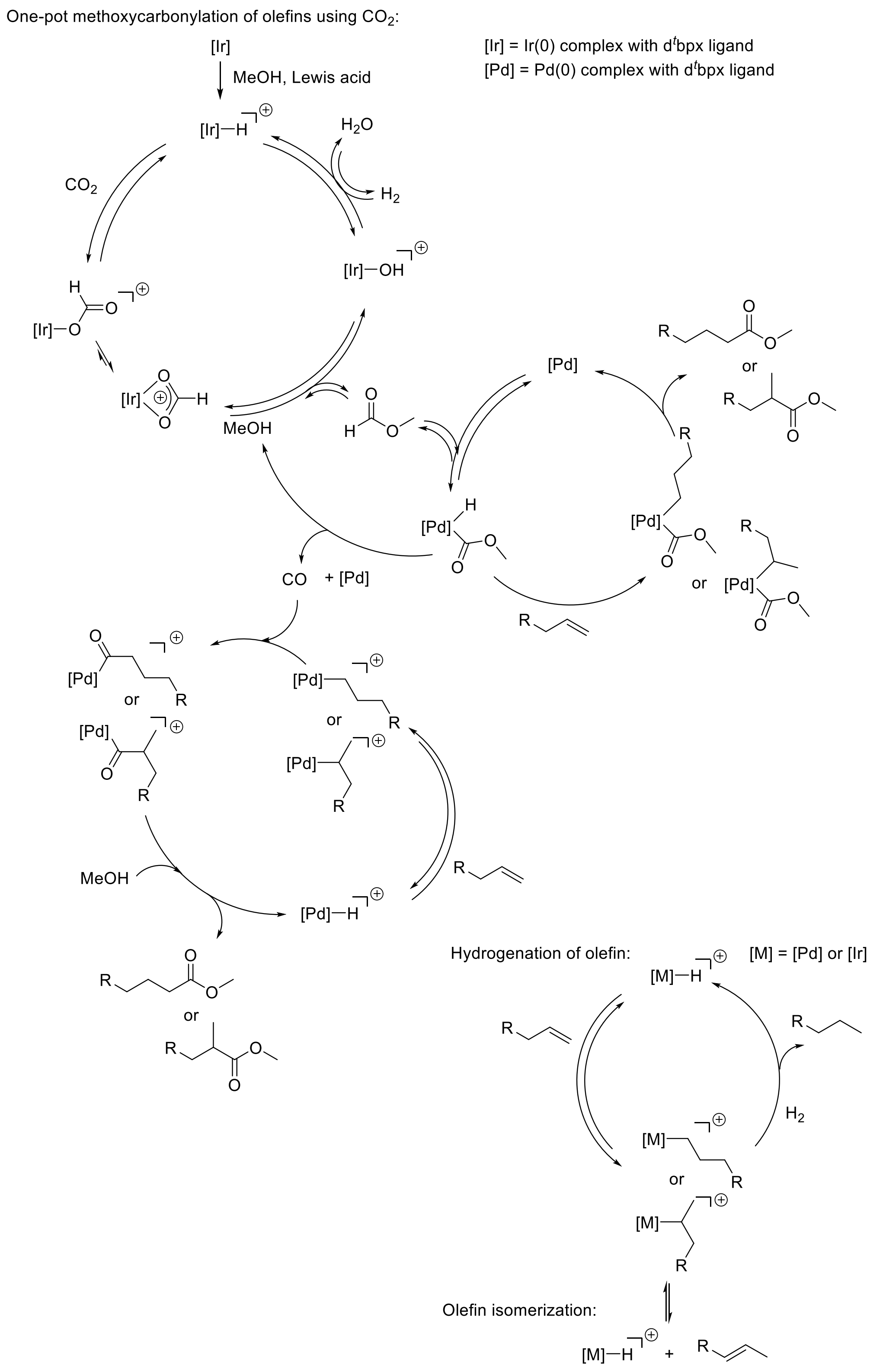

Fig. S25 | Proposed mechanism of Pd-Ir catalyzed methoxycarbonylation of olefin using CO_2 and H_2.

8. NMR data

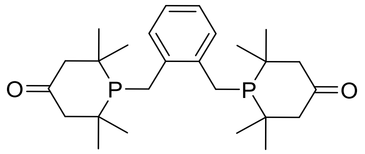

1,2-bis[(2,2,6,6-tetramethylphosphinan-4-onyl)methyl]benzene (BPX)
1H NMR (300 MHz, $CDCl_3$): δ 7.47–7.44 (m, 2H), 7.15–7.11 (m, 2H), 3.24 (t, J = 7.2 Hz, 4H), 2.56-2.51 (m, 4H), 2.40-2.30 (m, 4H), 1.20 (d, J = 5.4 Hz, 12H), 1.07 (d, J = 16.9 Hz, 12H); 13C NMR (75 MHz, $CDCl_3$): δ 209.9, 131.4, 131.2, 126.4, 55.5, 35.5, 35.2, 31.8, 31.5, 25.8; 31P NMR (121 MHz, $CDCl_3$): δ 5.6.

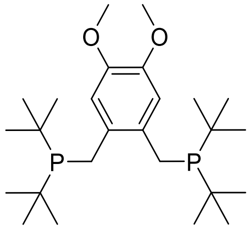

((4,5-(dimethoxy)-1,2-phenylene)bis(methylene))bis(di-tert-butylphosphine) (**L1**)
1H NMR (300 MHz, $CDCl_3$): δ 7.09 (d, J = 3.1 Hz, 2H), 3.84 (s, 6H), 2.95 (d, J = 2.5, 4H), 1.13 (d, J = 10.7 Hz, 36H); 13C NMR (75 MHz, $CDCl_3$): δ 146.4, 130.6, 114.2, 56.0, 31.2, 30.1, 26.1; 31P NMR (121 MHz, $CDCl_3$): δ 26.7.

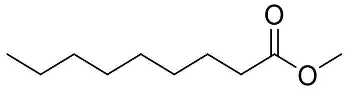

Methyl nonanoate (**2a**)
1H NMR (300 MHz, $CDCl_3$): δ 3.64 (s, 3H), 2.28 (t, J = 7.5 Hz, 2H), 1.59 (m, 2H), 1.26-1.08 (m, 10H), 0.85 (t, J = 6.6 Hz, 3H); 13C NMR (75 MHz, $CDCl_3$): δ 174.5, 51.6, 34.2, 32.0, 29.7, 29.6, 29.4, 29.4, 29.3, 25.1, 22.8, 14.2.

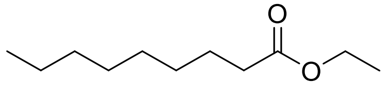

Ethyl nonanoate (**2b**)
1H NMR (300 MHz, $CDCl_3$): δ 4.11 (q, J = 7.1 Hz, 2H), 2.27 (t, J = 7.5 Hz, 2H), 1.68-1.54 (m, 2H), 1.30-1.19 (m, 13H), 0.90-0.83 (m, 3H); 13C NMR (75 MHz, $CDCl_3$): δ 174.0, 60.2, 34.5, 31.9, 29.3, 29.3, 29.2, 25.1, 22.7, 14.4, 14.1.

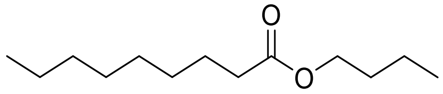

Butyl nonanoate (**2c**)
1H NMR (300 MHz, $CDCl_3$): δ 4.06 (t, J = 6.7 Hz, 2H), 2.28 (t, J = 7.5 Hz, 2H), 1.65-1.54 (m, 4H), 1.43-1.20 (m, 12H), 0.95-0.83 (m, 6H); 13C NMR (75 MHz, $CDCl_3$): δ 174.1, 64.2, 34.5, 31.9, 30.8, 29.3, 29.3, 29.2, 25.1, 22.7, 19.3, 14.2, 13.8.

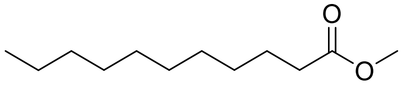

Methyl undecanoate (**2d**)
1H NMR (300 MHz, $CDCl_3$): δ 3.66 (s, 3H), 2.29 (t, *J* = 7.5 Hz, 2H), 1.61 (m, 2H), 1.31-1.21 (m, 14H), 0.87 (m, 3H); 13C NMR (75 MHz, $CDCl_3$): δ 174.4, 51.5, 34.2, 31.9, 29.3, 29.3, 29.2, 25.1, 22.7, 14.2.

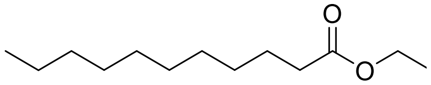

Ethyl undecanoate (**2e**)
1H NMR (300 MHz, $CDCl_3$): δ 4.10 (q, *J* = 7.1 Hz, 2H), 2.27 (t, *J* = 7.5 Hz, 2H), 1.65-1.53 (m, 2H), 1.32-1.20 (m, 17H), 0.89-0.82 (m, 3H); 13C NMR (75 MHz, $CDCl_3$): δ 174.0, 60.2, 34.5, 32.0, 29.7, 29.6, 29.4, 29.4, 29.3, 25.1, 22.8, 14.4, 14.2.

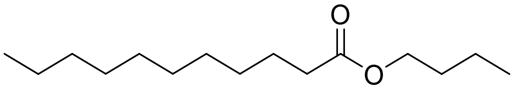

Butyl undecanoate (**2f**)
1H NMR (300 MHz, $CDCl_3$): δ 4.05 (t, *J* = 6.7 Hz, 2H), 2.27 (t, *J* = 7.5 Hz, 2H), 1.65-1.54 (m, 4H), 1.43-1.21 (m, 16H), 0.94-0.84 (m, 6H); 13C NMR (75 MHz, $CDCl_3$): δ 174.1, 64.2, 34.5, 32.0, 30.8, 29.7, 29.6, 29.4, 29.4, 29.3, 25.1, 22.8, 19.3, 14.2, 13.8.

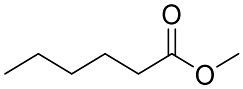

Methyl hexanoate (**2g**)
1H NMR (300 MHz, $CDCl_3$): δ 3.65 (s, 3H), 2.29 (t, *J* = 7.6 Hz, 2H), 1.61 (m, 2H), 1.36-1.23 (m, 4H), 0.88 (t, *J* = 6.6 Hz, 3H); 13C NMR (75 MHz, $CDCl_3$): δ 174.6, 51.5, 34.2, 31.4, 24.8, 22.4, 14.0.

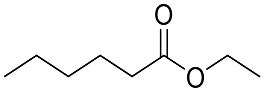

Ethyl hexanoate (**2h**)
1H NMR (300 MHz, $CDCl_3$): δ 4.11 (q, *J* = 7.1 Hz, 2H), 2.27 (t, *J* = 7.5 Hz, 2H), 1.67-1.54 (m, 2H), 1.34-1.22 (m, 7H), 0.91-0.82 (m, 3H); 13C NMR (75 MHz, $CDCl_3$): δ 174.0, 60.3, 34.5, 31.5, 24.8, 22.5, 14.4, 14.0.

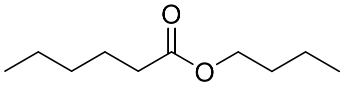

Butyl hexanoate (**2i**)

1H NMR (300 MHz, $CDCl_3$): δ 4.06 (t, J = 6.7 Hz, 2H), 2.27 (t, J = 7.5 Hz, 2H), 1.67-1.54 (m, 4H), 1.43-1.23 (m, 6H), 0.95-0.86 (m, 6H); 13C NMR (75 MHz, $CDCl_3$): δ 174.1, 64.2, 34.5, 31.4, 30.8, 24.1, 22.4, 19.3, 14.0, 13.8.

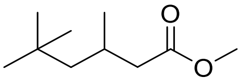

Methyl 3,5,5-trimethylhexanoate (**2j**)
1H NMR (300 MHz, $CDCl_3$): δ 3.66 (s, 3H), 2.34-2.27 (m, 1H), 2.17-1.97 (m, 2H), 1.25 (m, 10H), 0.97 (d, J = 6.5 Hz, 3H), 0.90 (s, 9H); 13C NMR (75 MHz, $CDCl_3$): δ 173.8, 51.4, 50.7, 44.0, 31.2, 30.0, 27.1, 22.8.

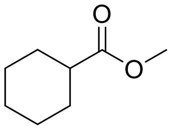

Methyl cyclohexanecarboxylate (**2k**)
1H NMR (300 MHz, $CDCl_3$): δ 3.66 (s, 3H), 2.33-2.24 (m, 1H), 1.91-1.22 (m, 10H); 13C NMR (75 MHz, $CDCl_3$): δ 176.7, 51.6, 43.3, 29.2, 25.9, 25.6.

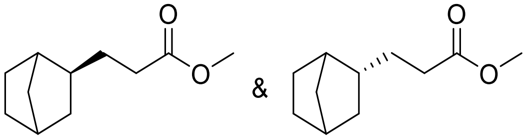

exo:endo = 65:35

Methyl 3-((2R)-bicyclo[2.2.1]heptan-2-yl)propanoate (**2l-*exo***) &
Methyl 3-((2S)-bicyclo[2.2.1]heptan-2-yl)propanoate (**2l-*endo***)
The NMR data match the references.[3]
1H NMR (300 MHz, $CDCl_3$): δ 3.62 (s, 3H), 2.23 (t, J = 7.6 Hz, 2H), 2.16-1.90 (m, 2H), 1.72-0.53 (m, 11H); 13C NMR (75 MHz, $CDCl_3$): *exo:* δ 174.4, 51.5, 41.8, 41.0, 37.9, 36.6, 35.3, 32.7, 31.9, 30.1, 28.8; *endo:* δ 174.4, 51.5, 39.9, 39.7, 39.6, 37.1, 36.9, 33.5, 30.1, 28.1, 22.3.

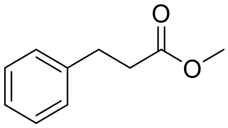

Methyl 3-phenylpropanoate (**2m**)
1H NMR (300 MHz, $CDCl_3$): δ 7.27-7.13 (m, 5H), 3.62 (s, 3H), 2.91 (t, J = 7.8 Hz, 2H), 2.59 (t, J = 8.0 Hz, 2H); 13C NMR (75 MHz, $CDCl_3$): δ 173.4, 140.6, 128.6, 128.4, 126.4, 51.7, 35.8, 31.0.

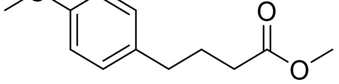

Methyl 4-(4-methoxyphenyl)butanoate (**2n**)

1H NMR (300 MHz, $CDCl_3$): δ 7.12-7.06 (m, 2H), 6.85-6-80 (m, 2H), 3.78 (s, 3H), 3.66 (s, 3H), 2.60 (t, J = 7.6 Hz, 2H), 2.32 (t, J = 7.5 Hz, 2H), 1.92 (m, 2H); 13C NMR (75 MHz, $CDCl_3$): δ 174.1, 158.0, 133.5, 129.5, 113.9, 55.3, 51.6, 34.3, 33.4, 26.8.

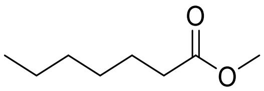

Methyl heptanoate (**2o**)
1H NMR (300 MHz, $CDCl_3$): δ 3.65 (s, 3H), 2.29 (t, J = 7.5 Hz, 2H), 1.65-1.55 (m, 2H), 1.35-1.22 (m, 6H), 0.91-0.83 (m, 3H); 13C NMR (75 MHz, $CDCl_3$): δ 174.4, 51.5, 34.2, 31.6, 28.9, 25.0, 22.6, 14.1.

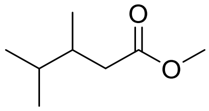

Methyl 3,4-dimethylpentanoate (**2q**)
1H NMR (300 MHz, $CDCl_3$): δ 3.65 (s, 3H), 2.34 (dd, J_1 = 5.2 Hz, J_2 = 14.6 Hz, 1H), 2.06 (dd, J_1 = 9.2 Hz, J_2 = 14.6 Hz, 1H), 1.93-1.80 (m, 1H), 0.88-0.82 (m, 9H); 13C NMR (75 MHz, $CDCl_3$): δ 174.3, 51.5, 39.1, 36.0, 32.2, 19.9, 18.4, 15.9.

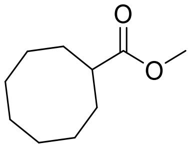

Methyl cyclooctanecarboxylate (**2r**)
1H NMR (300 MHz, $CDCl_3$): δ 3.65 (s, 3H), 2.55-2.42 (m, 1H), 1.88-1.53 (m, 14H); 13C NMR (75 MHz, $CDCl_3$): δ 178.0, 51.7, 43.6, 28.9, 26.9, 26.3, 25.4.

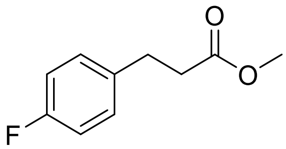

Methyl 3-(4-fluorophenyl)propanoate (**2s**)
1H NMR (300 MHz, $CDCl_3$): δ 7.17-7.13 (m, 2H), 7.00-6.92 (m, 2H), 3.66 (s, 3H), 2.92 (t, J = 7.7 Hz, 2H), 2.60 (t, J = 7.7 Hz, 2H); 13C NMR (75 MHz, $CDCl_3$): δ 173.3, 161.2, 136.2, 129.8, 115.3, 51.7, 35.8, 30.2.

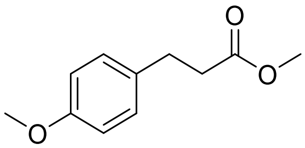

Methyl 3-(4-methoxyphenyl)propanoate (**2t**)
1H NMR (300 MHz, $CDCl_3$): δ 7.12 (d, J = 8.8, 2H), 6.83 (d, J = 8.7, 2H), 3.78 (s, 3H), 3.66 (s, 3H), 2.90 (t, J = 7.8 Hz, 2H), 2.60 (t, J = 7.7 Hz, 2H); 13C NMR (75 MHz, $CDCl_3$): δ 173.5, 158.2, 132.7, 129.3, 114.0, 55.3, 51.7, 36.1, 30.2.

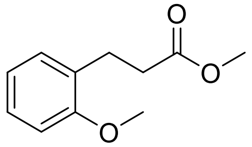

Methyl 3-(2-methoxyphenyl)propanoate (**2u**)
1H NMR (300 MHz, $CDCl_3$): δ 7.22-7.13 (m, 2H), 6.90-6.83 (m, 2H), 3.83 (s, 3H), 3.67 (s, 3H), 2.95 (t, *J* = 7.8 Hz, 2H), 2.62 (t, *J* = 7.8 Hz, 2H); 13C NMR (75 MHz, $CDCl_3$): δ 174.0, 157.6, 130.3, 128.9, 127.7, 120.5, 110.3, 55.3, 51.6, 34.1, 26.3.

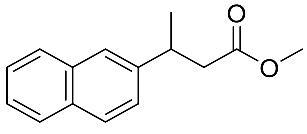

Methyl 3-(naphthalen-2-yl)butanoate (**2v**)
1H NMR (300 MHz, $CDCl_3$): δ 7.86-7.80 (m, 3H), 7.70 (d, *J* = 1.5 Hz, 1H), 7.51-7.39 (m, 3H), 3.65 (s, 3H), 3.50 (m, 1H), 2.82-2.64 (m, 2H), 1.43 (d, *J* = 7.0 Hz, 3H); 13C NMR (75 MHz, $CDCl_3$): δ 172.8, 143.2, 133.6, 132.4, 128.3, 127.7, 127.6, 126.1, 125.5, 125.5, 124.5, 51.6, 42.7, 36.6, 21.9.

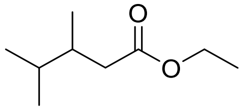

Ethyl 3,4-dimethylpentanoate (**2w**)
1H NMR (300 MHz, $CDCl_3$): δ 4.12 (q, *J* = 7.1 Hz, 2H), 2.36-2.25 (m, 1H), 2.10-2.01 (m, 1H), 1.93-1.82 (m, 1H), 1.64-1.52 (m, 1H), 1.27-1.21 (m, 4H), 0.88-0.83 (m, 8H); 13C NMR (75 MHz, $CDCl_3$): δ 173.9, 60.2, 39.4, 36.0, 32.2, 19.9, 18.3, 15.9, 14.4.

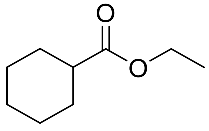

Ethyl cyclohexanecarboxylate (**2x**)
1H NMR (300 MHz, $CDCl_3$): δ 4.10 (q, *J* = 7.1 Hz, 2H), 2.27 (tt, J_1 = 7.2 Hz, J_2 = 11.2 Hz, 1H), 1.91-1.82 (m, 2H), 1.77-1.69 (m, 2H), 1.50-1.21 (m, 9H); 13C NMR (75 MHz, $CDCl_3$): δ 176.3, 61.1, 43.4, 29.1, 25.9, 25.6, 14.4.

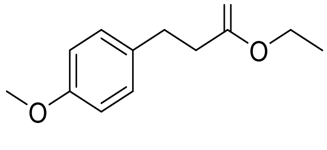

Ethyl 3-(4-methoxyphenyl)propanoate (**2y**)
1H NMR (300 MHz, $CDCl_3$): δ 7.14-7.09 (m, 2H), 6.85-6.80 (m, 2H), 4.12 (q, *J* = 7.1 Hz, 2H), 3.678 (s, 3H), 2.90 (t, *J* = 7.8 Hz, 2H), 2.59 (t, *J* = 7.9 Hz, 2H), 1.23 (t, *J* = 7.1 Hz, 3H); 13C NMR (75 MHz, $CDCl_3$): δ 173.1, 158.2, 132.8, 129.4, 114.0, 60.5, 55.4, 36.4, 30.3, 14.3.

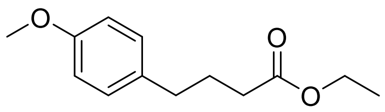

Ethyl 4-(4-methoxyphenyl)butanoate (**2z**)
1H NMR (300 MHz, $CDCl_3$): δ 7.10 (d, J = 8.7 Hz, 2H), 6.83 (d, J = 8.7 Hz, 2H), 4.13 (q, J = 7.1 Hz, 2H), 3.78 (s, 3H), 2.60 (t, J = 7.6 Hz, 2H), 2.31 (t, J = 7.5 Hz, 2H), 1.98-1.87 (m, 2H), 1.25 (t, J = 7.2 Hz, 3H); 13C NMR (75 MHz, $CDCl_3$): δ 173.6, 158.0, 133.6, 129.4, 113.8, 60.3, 55.3, 34.3, 33.7, 26.9, 14.3.

9. NMR spectra

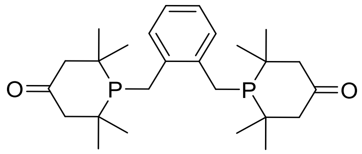

1,2-bis[(2,2,6,6-tetramethylphosphinan-4-onyl)methyl]benzene (BPX)

1H NMR (300 MHz, $CDCl_3$):

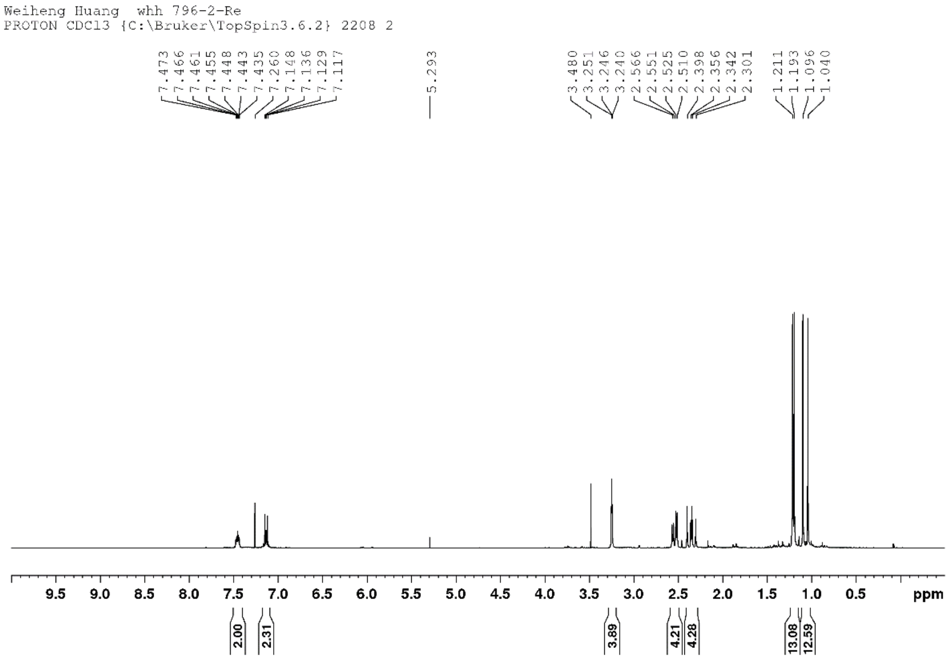

13C NMR (75 MHz, $CDCl_3$):

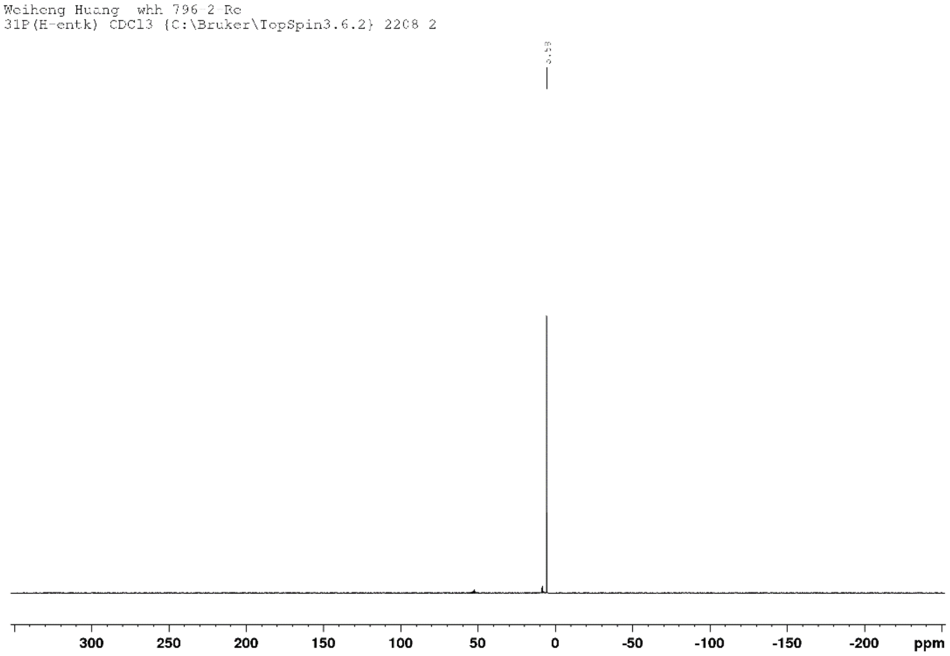

31P NMR (121 MHz, $CDCl_3$):

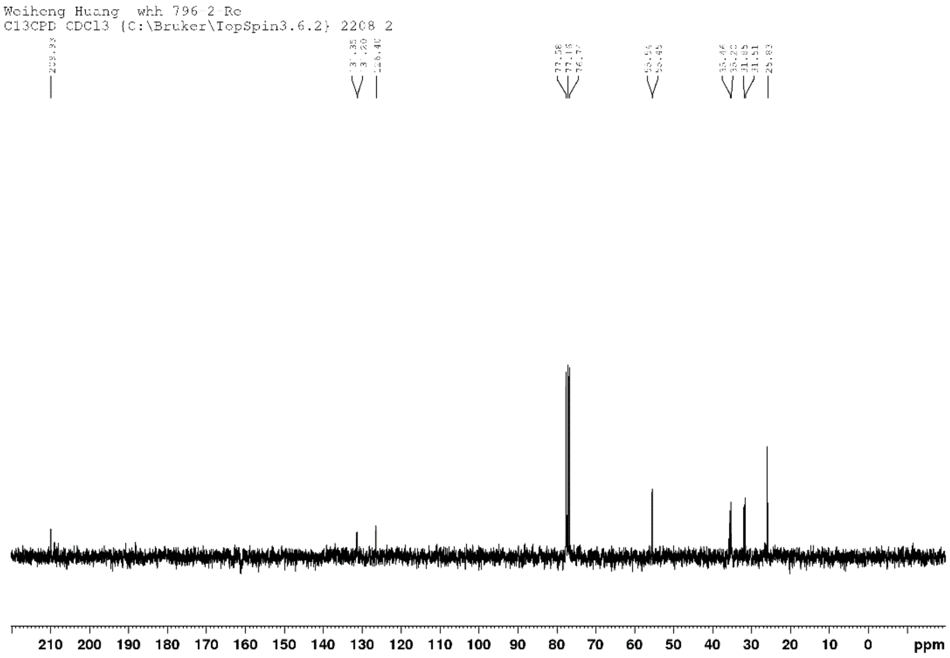

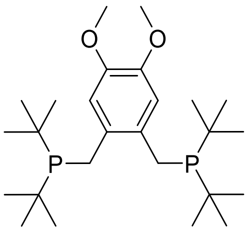

((4,5-(dimethoxy)-1,2-phenylene)bis(methylene))bis(di-tert-butylphosphine) (**L1**)

1H NMR (300 MHz, $CDCl_3$):

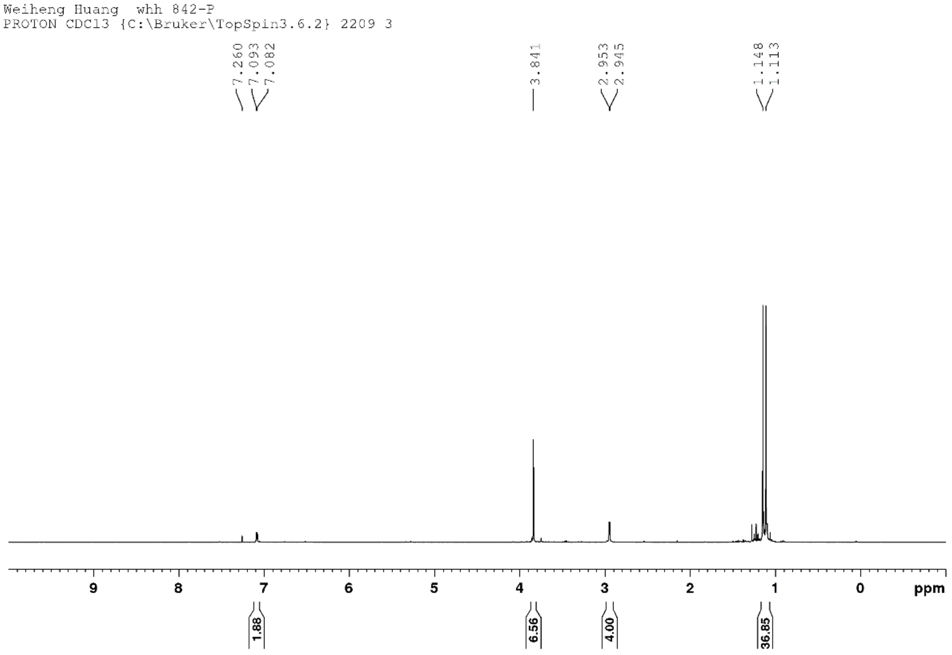

13C NMR (75 MHz, $CDCl_3$):

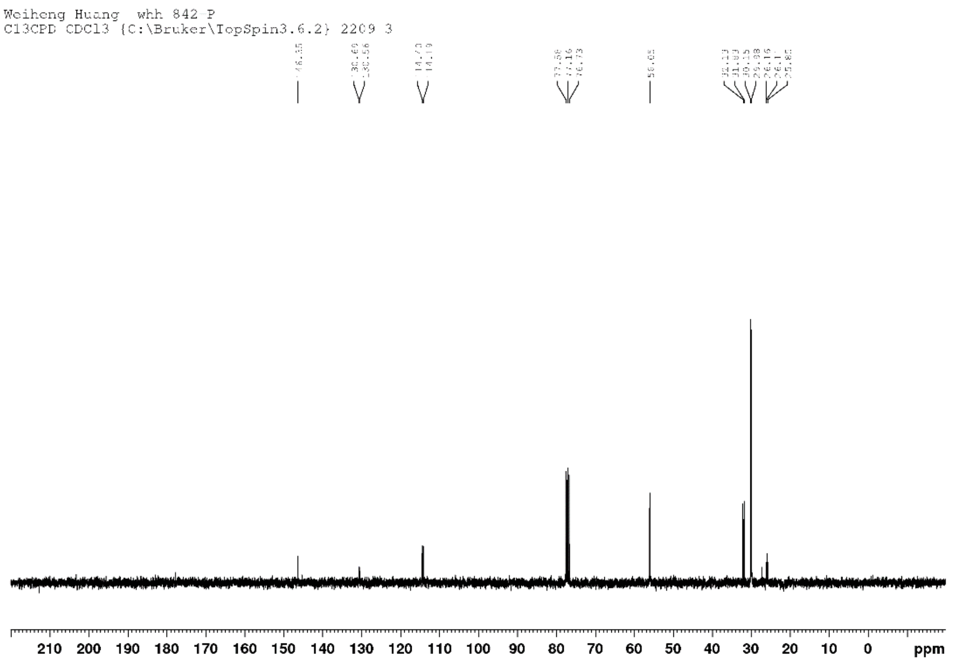

31P NMR (121 MHz, $CDCl_3$):

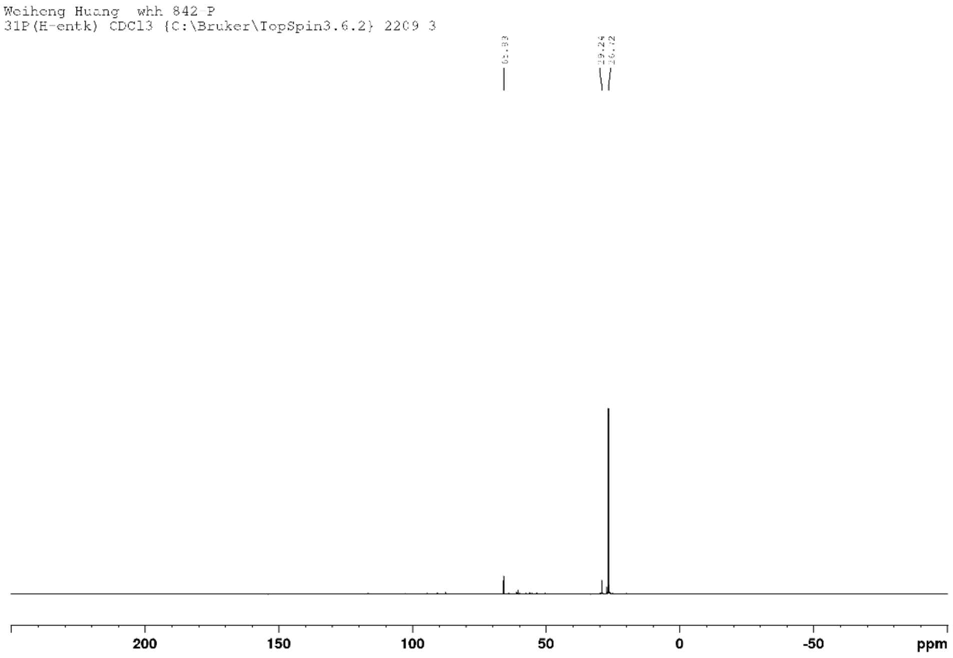

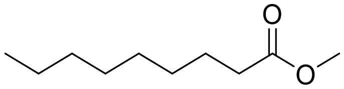

Methyl nonanoate (**2a**)
1H NMR (300 MHz, $CDCl_3$):

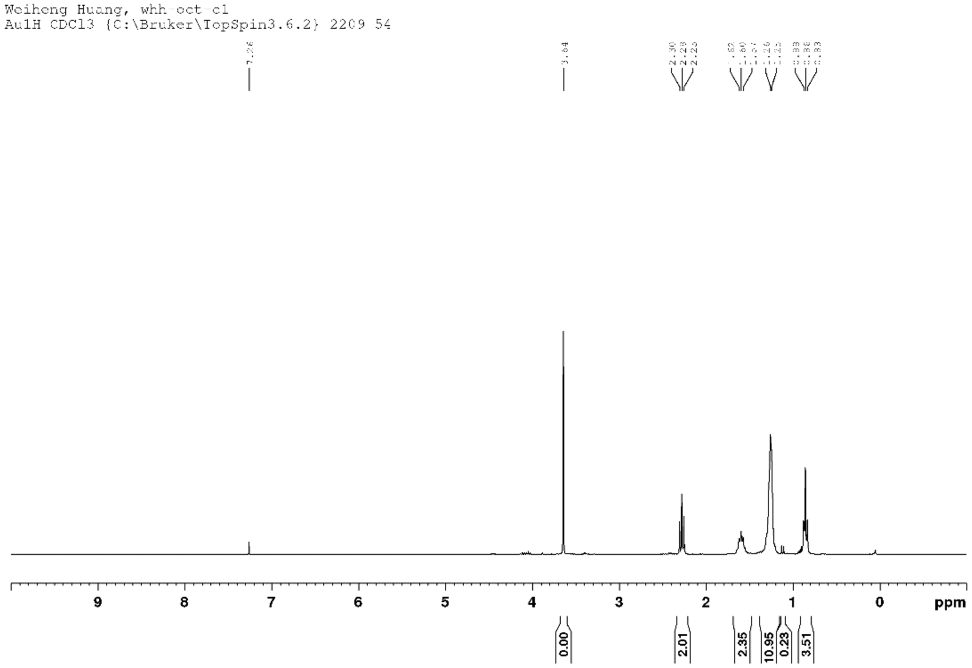

13C NMR (75 MHz, $CDCl_3$):

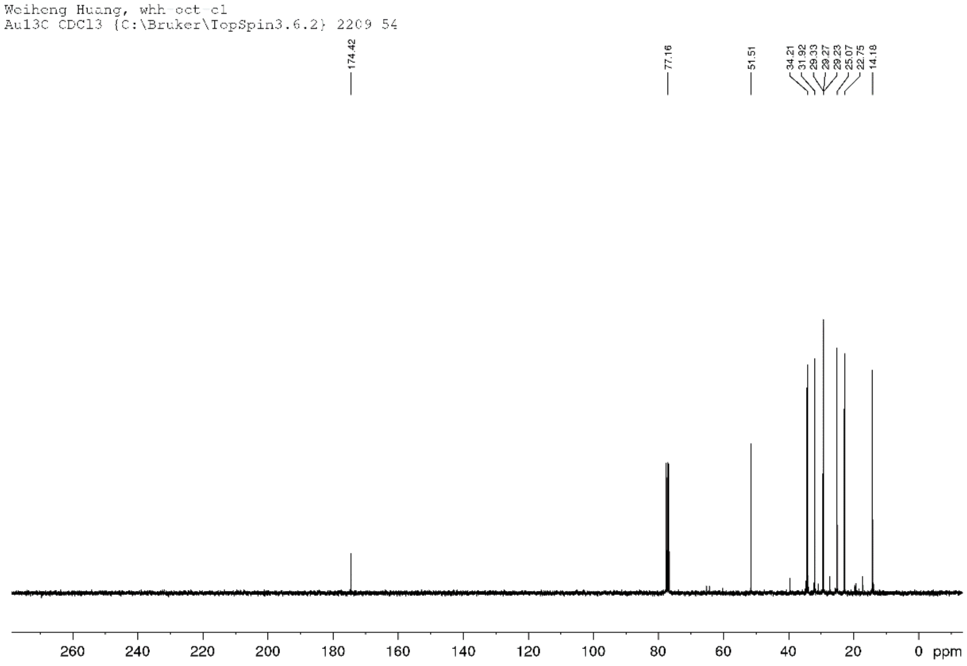

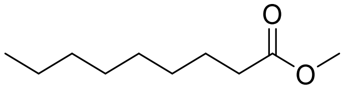

Methyl nonanoate (**2a**) and branched isomers (from 1,7-octadiene substrate), *n/i* = 90:10.
1H NMR (300 MHz, $CDCl_3$):

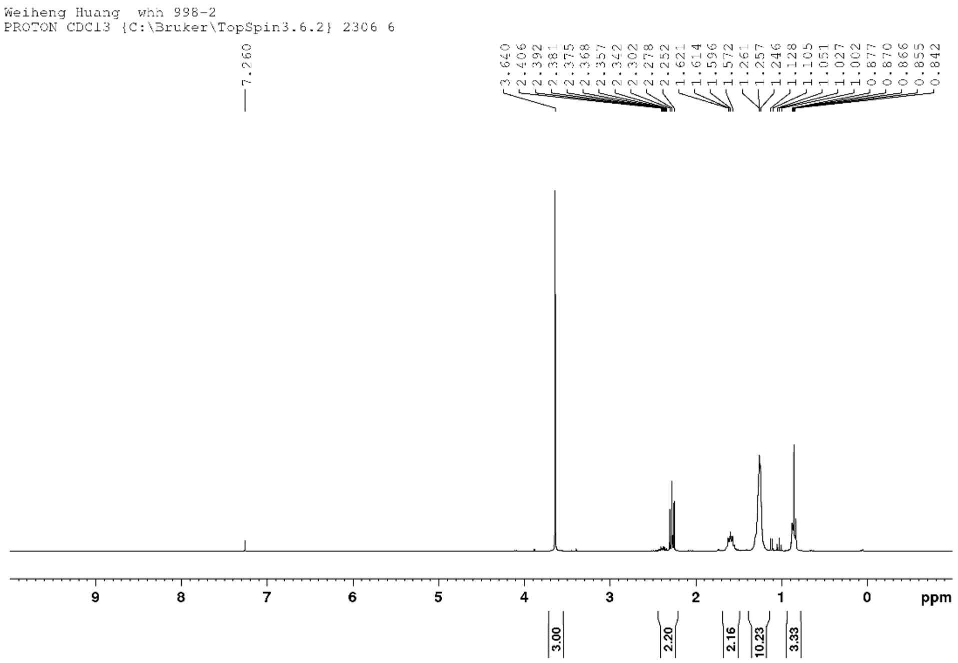

13C NMR (75 MHz, $CDCl_3$):

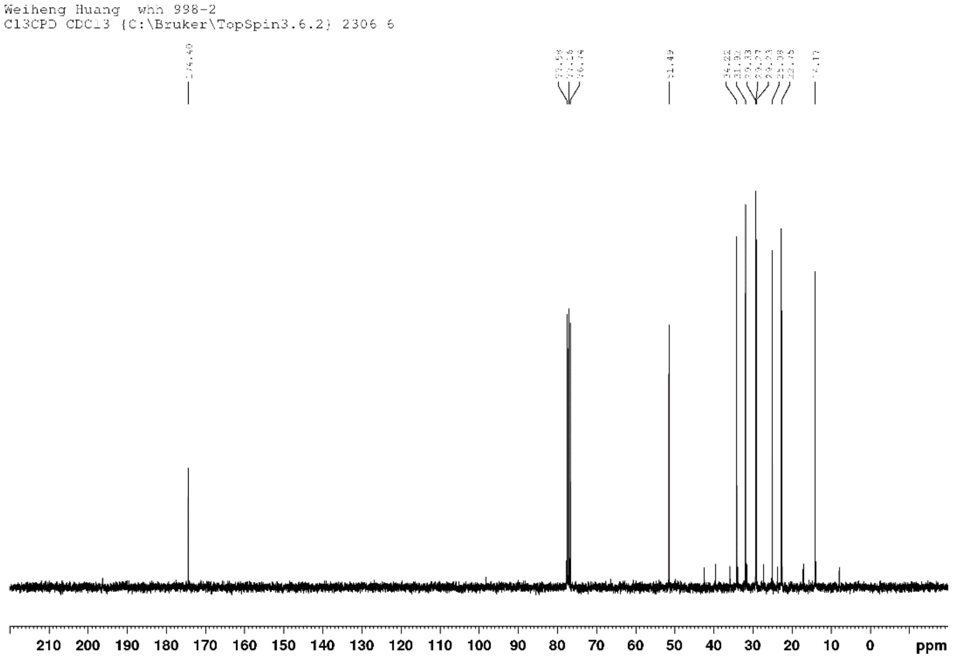

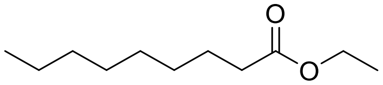

Ethyl nonanoate (**2b**)

1H NMR (300 MHz, $CDCl_3$):

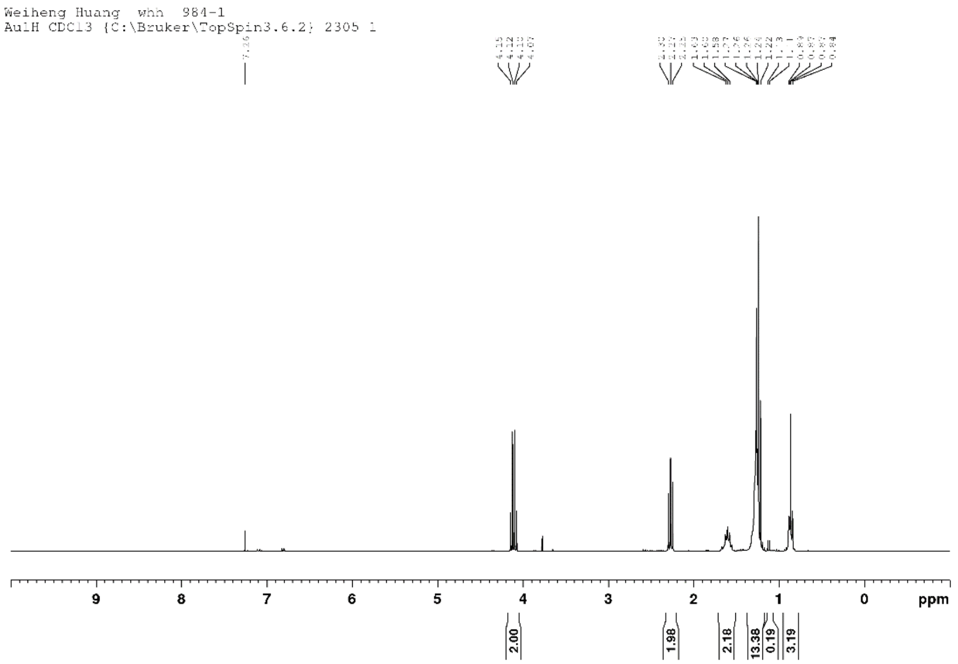

13C NMR (75 MHz, $CDCl_3$):

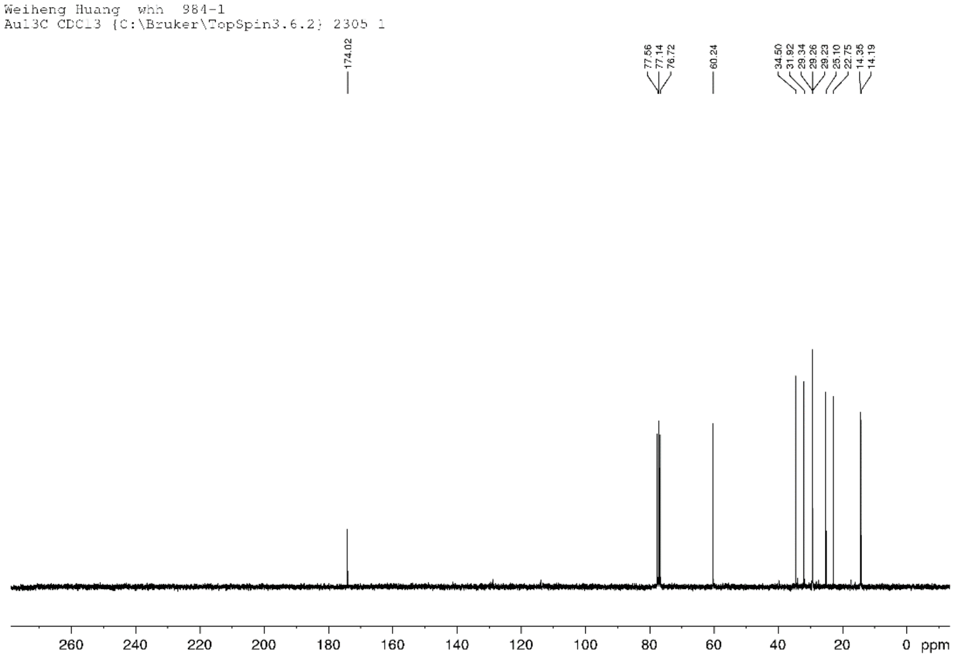

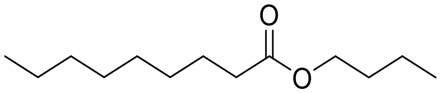

Butyl nonanoate (**2c**)

1H NMR (300 MHz, $CDCl_3$):

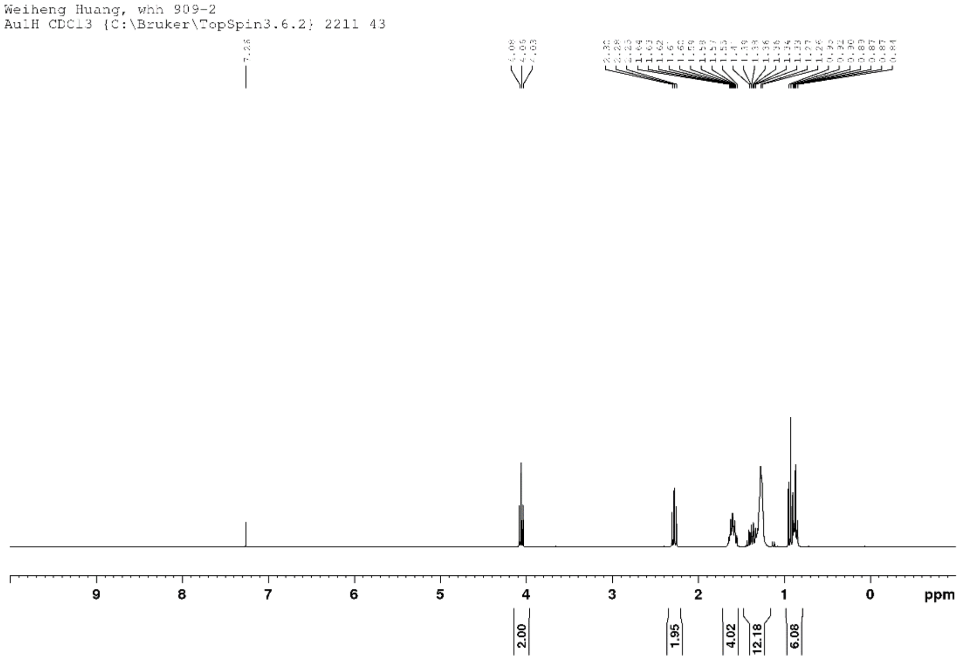

13C NMR (75 MHz, $CDCl_3$):

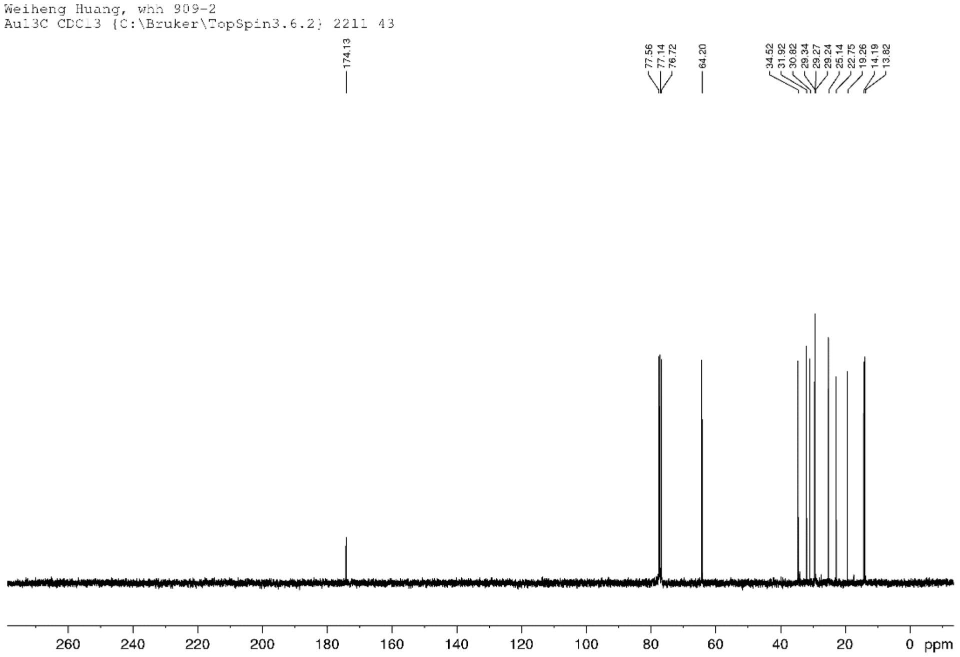

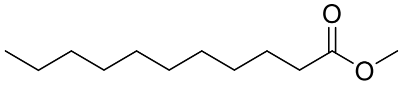

Methyl undecanoate (**2d**)
1H NMR (300 MHz, $CDCl_3$):

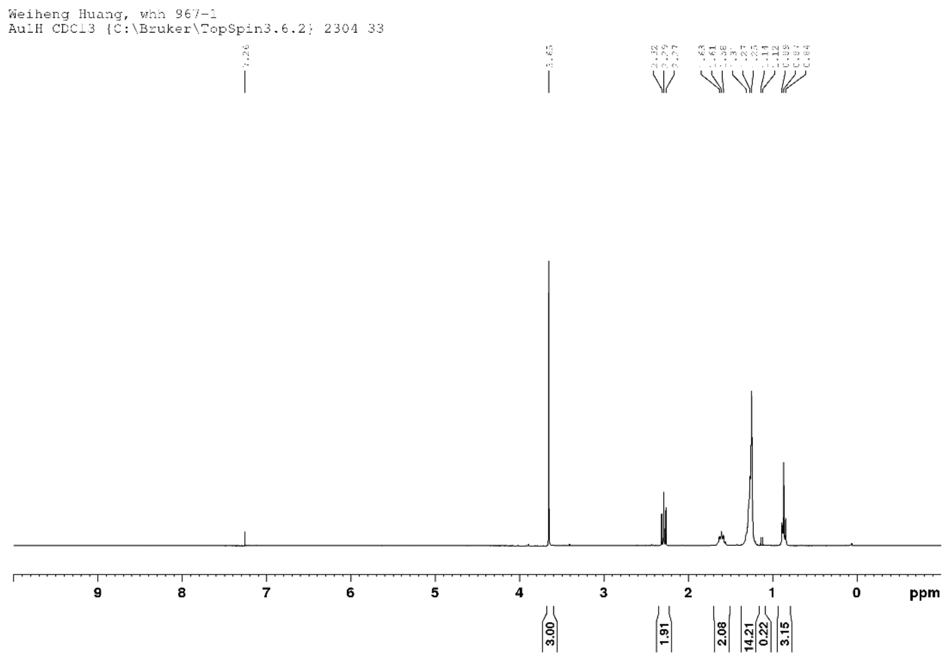

13C NMR (75 MHz, $CDCl_3$):

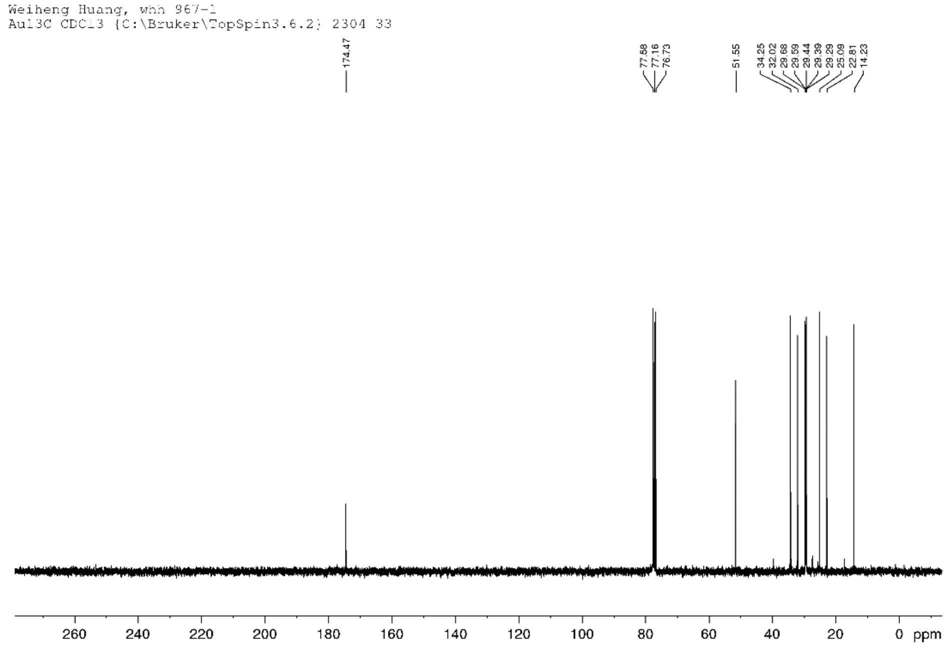

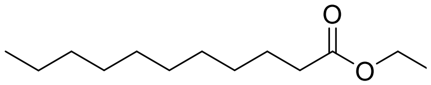

Ethyl undecanoate (**2e**)
1H NMR (300 MHz, $CDCl_3$):

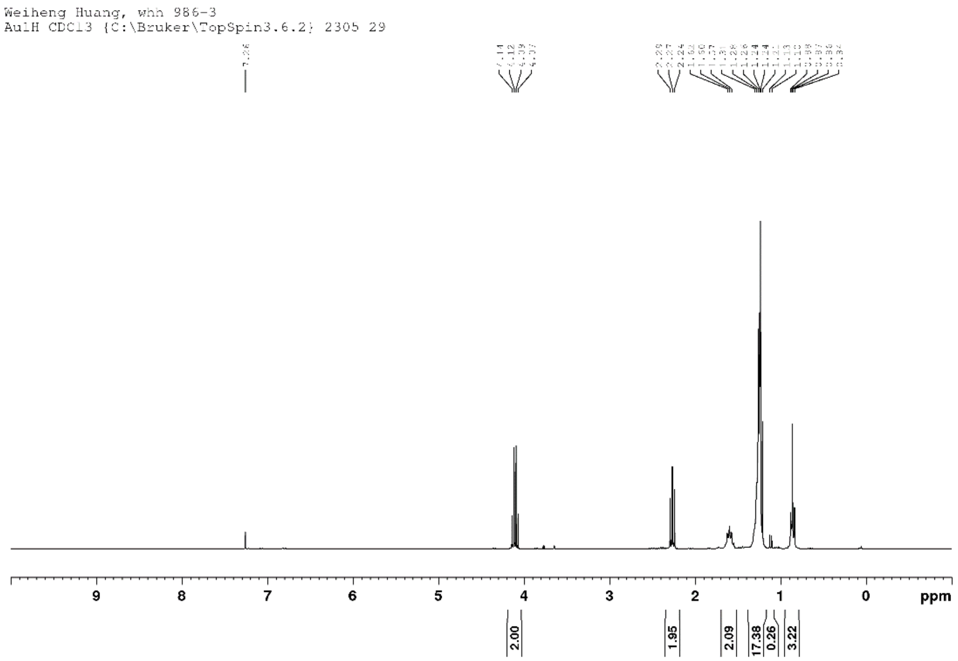

13C NMR (75 MHz, $CDCl_3$):

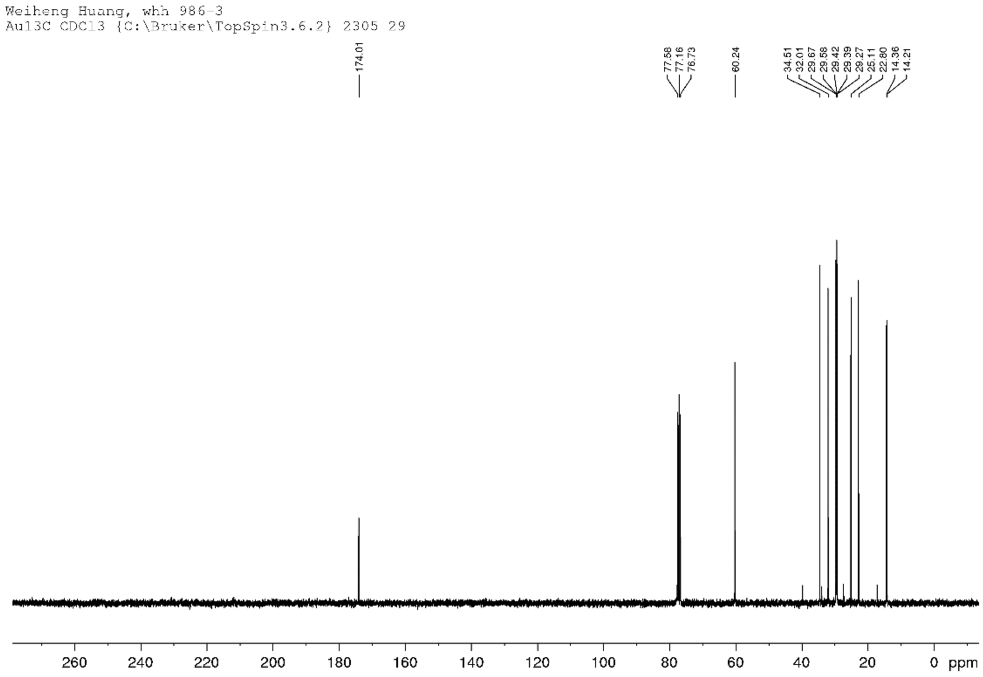

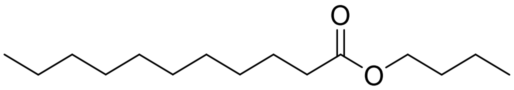

Butyl undecanoate (**2f**)

1H NMR (300 MHz, $CDCl_3$):

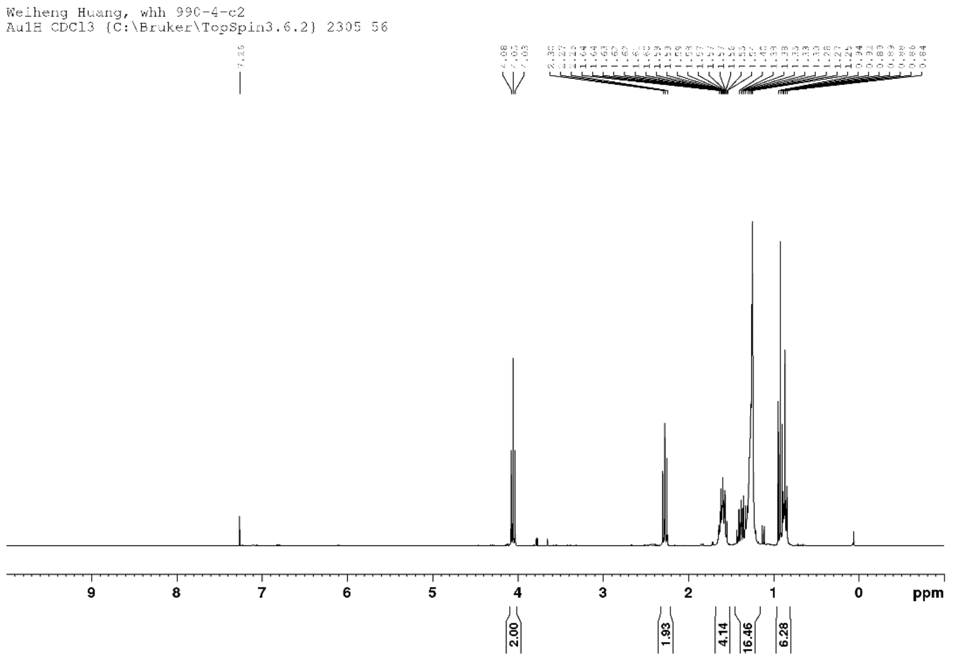

13C NMR (75 MHz, $CDCl_3$):

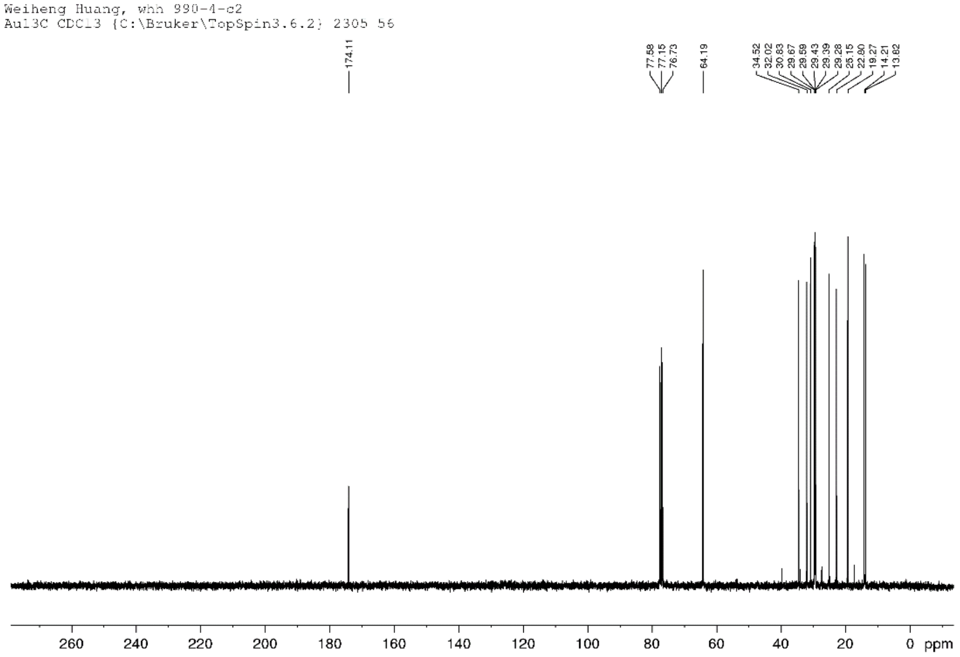

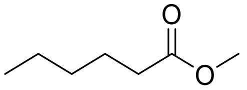

Methyl hexanoate (**2g**)

1H NMR (300 MHz, $CDCl_3$):

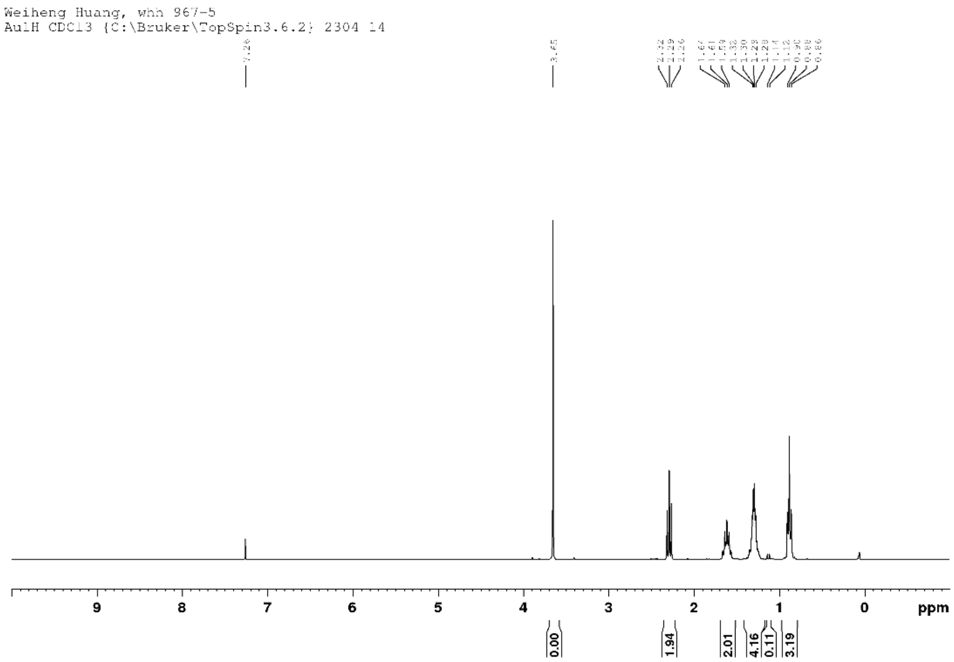

13C NMR (75 MHz, $CDCl_3$):

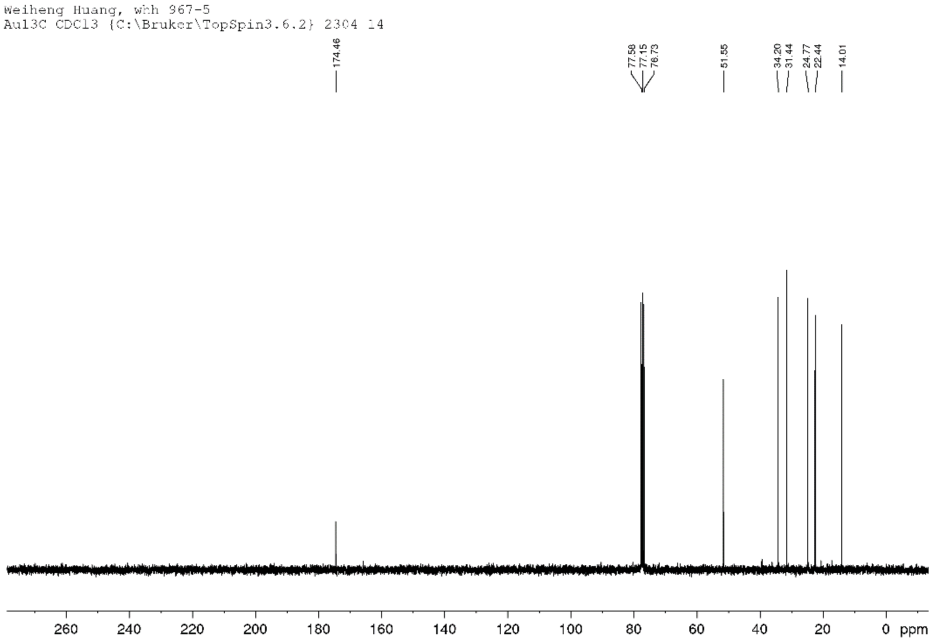

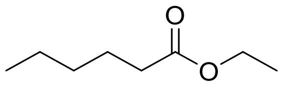

Ethyl hexanoate (**2h**)

1H NMR (300 MHz, $CDCl_3$):

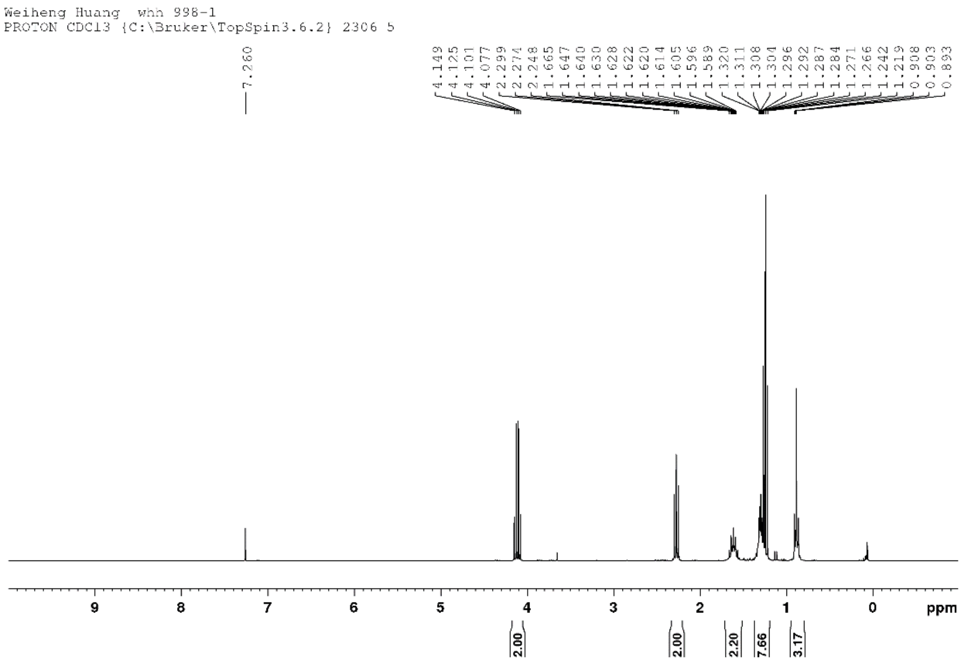

13C NMR (75 MHz, $CDCl_3$):

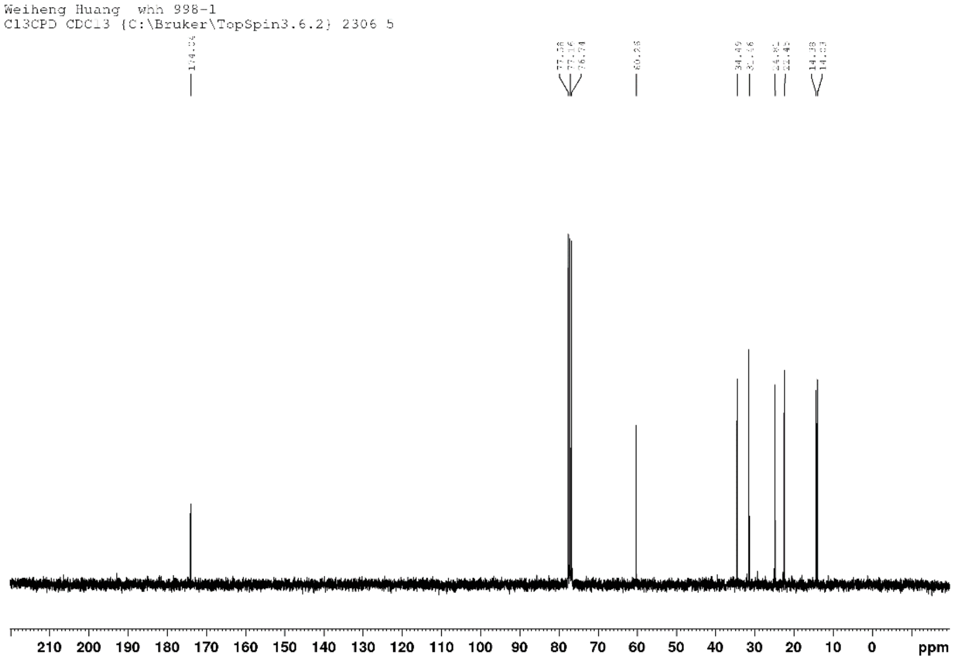

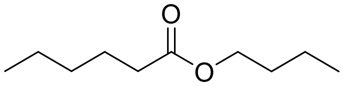

Butyl hexanoate (**2i**)

1H NMR (300 MHz, $CDCl_3$):

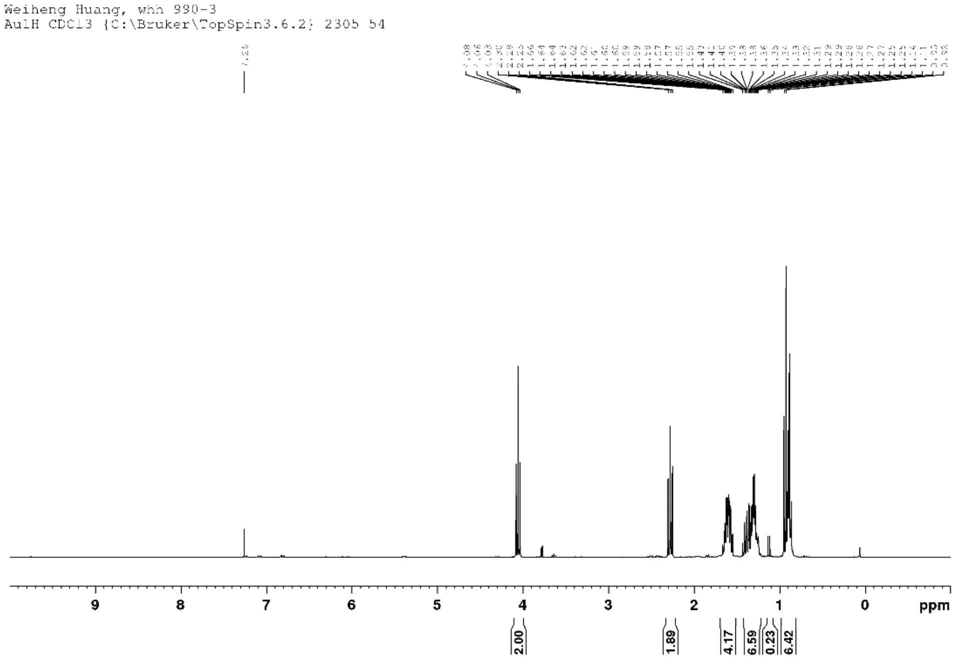

13C NMR (75 MHz, $CDCl_3$):

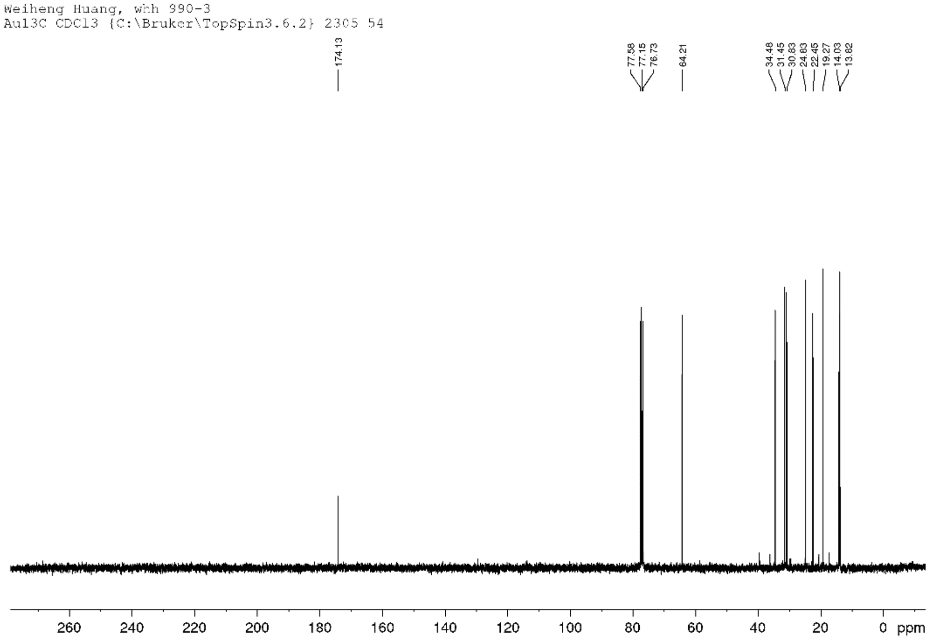

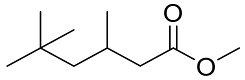

Methyl 3,5,5-trimethylhexanoate (**2j**)
1H NMR (300 MHz, $CDCl_3$):

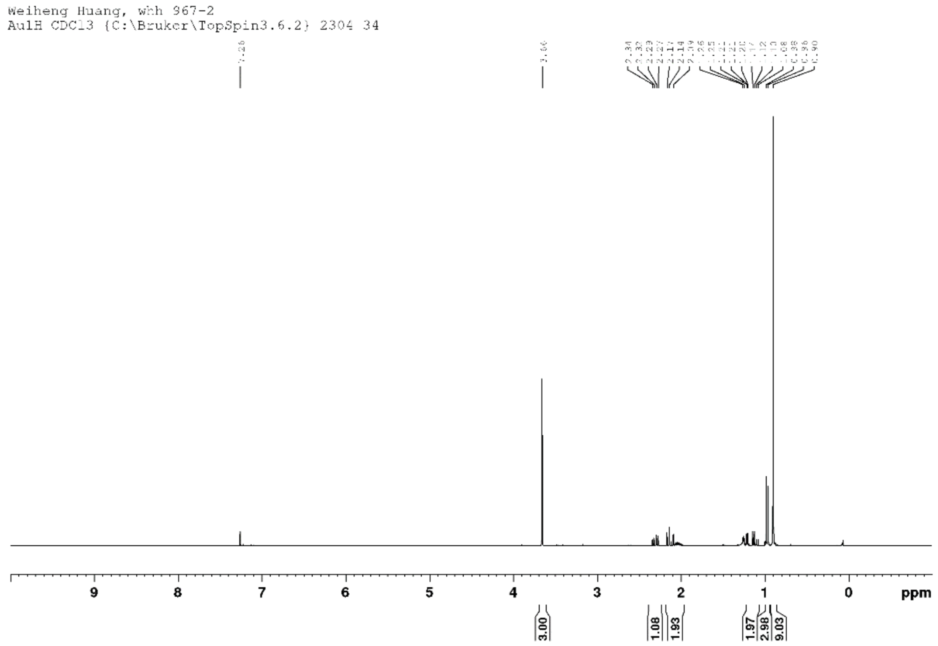

13C NMR (75 MHz, $CDCl_3$):

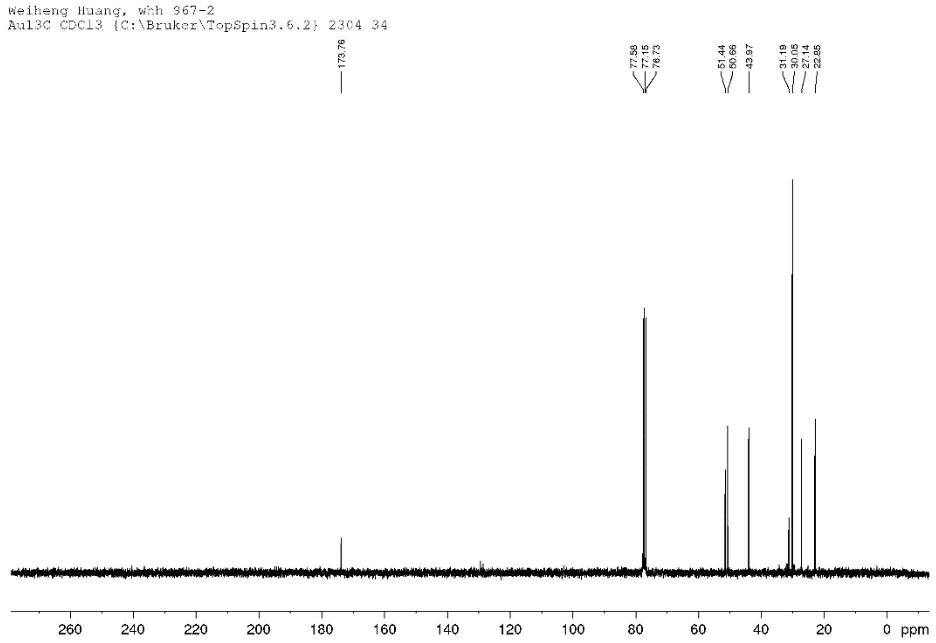

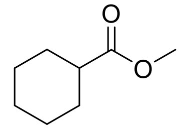

Methyl cyclohexanecarboxylate (**2k**)
1H NMR (300 MHz, $CDCl_3$):

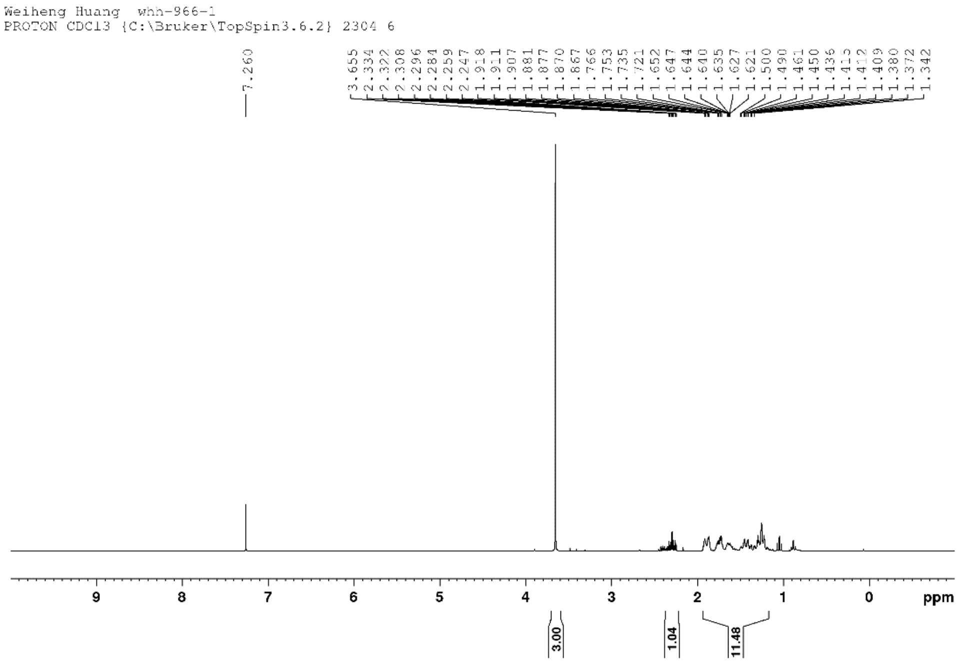

13C NMR (75 MHz, $CDCl_3$):

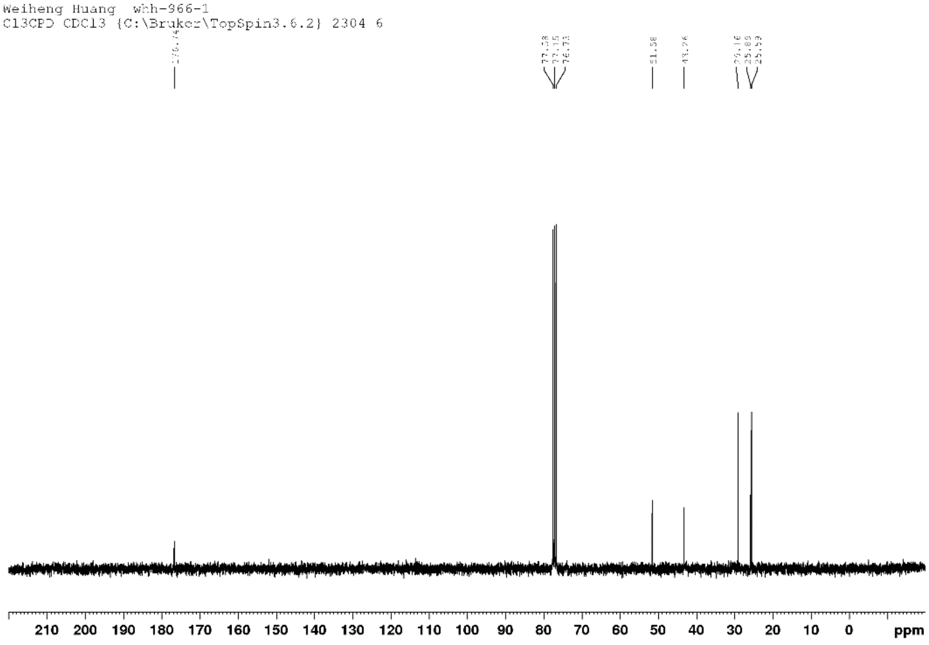

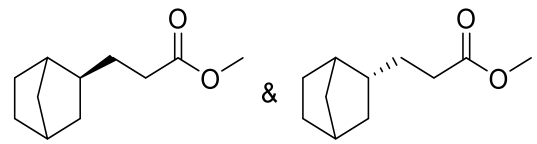

exo:endo = 62:38

Methyl 3-((2S)-bicyclo[2.2.1]heptan-2-yl)propanoate (**2l-*exo***) and
Methyl 3-((2R)-bicyclo[2.2.1]heptan-2-yl)propanoate (**2l-*endo***), *exo:endo* = 62:38.
1H NMR (300 MHz, $CDCl_3$):

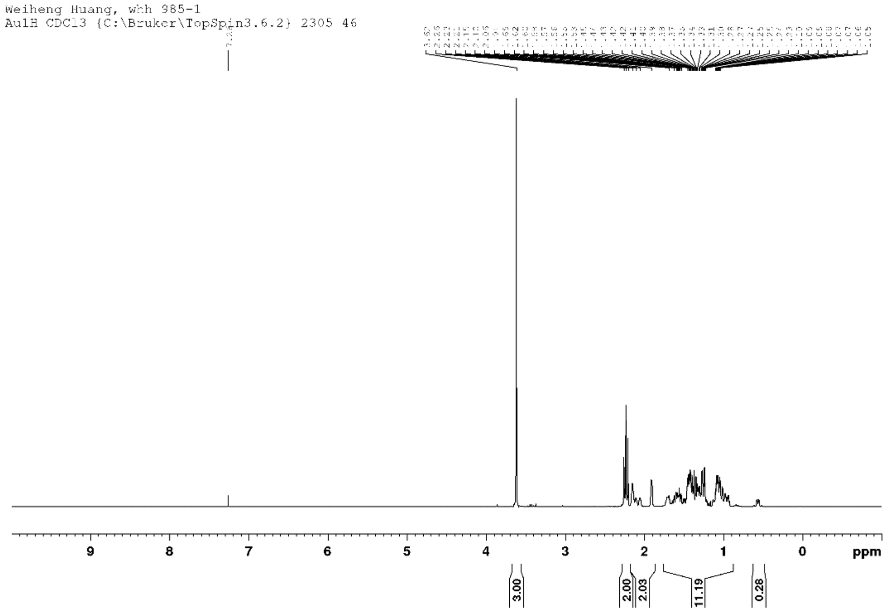

13C NMR (75 MHz, $CDCl_3$):

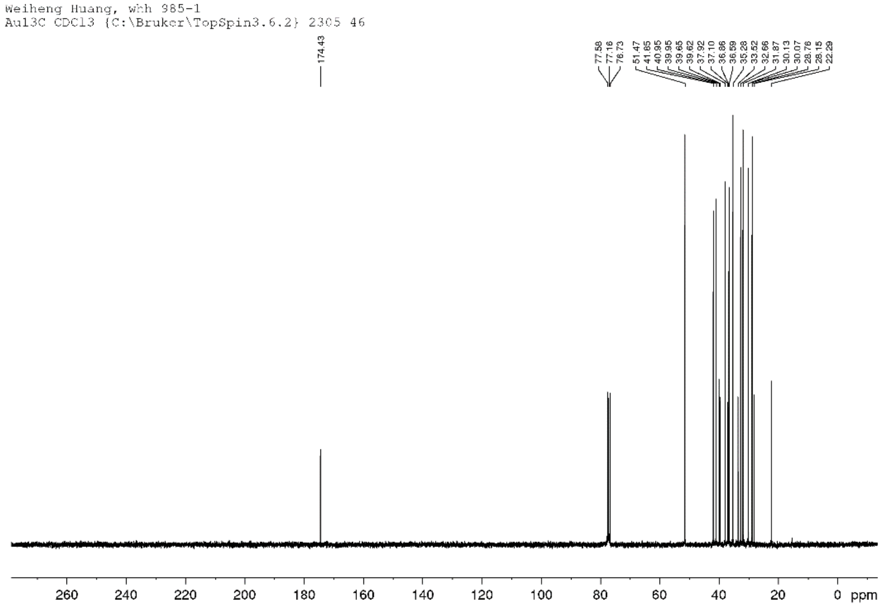

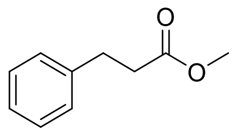

Methyl 3-phenylpropanoate (**2m**)

1H NMR (300 MHz, $CDCl_3$):

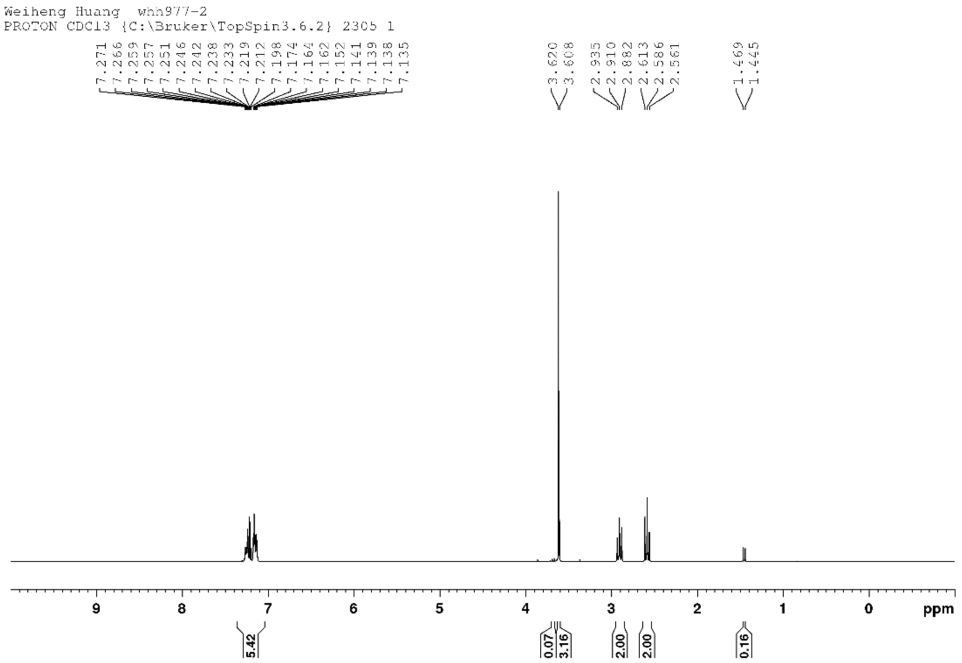

13C NMR (75 MHz, $CDCl_3$):

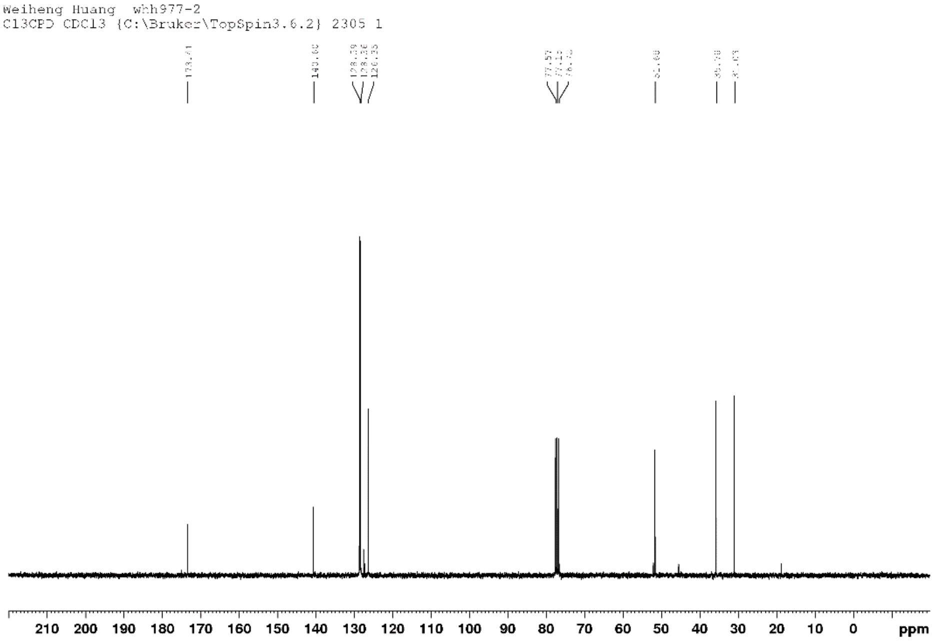

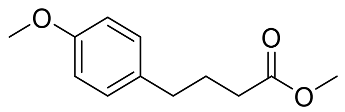

Methyl 4-(4-methoxyphenyl)butanoate (**2n**)

1H NMR (300 MHz, $CDCl_3$):

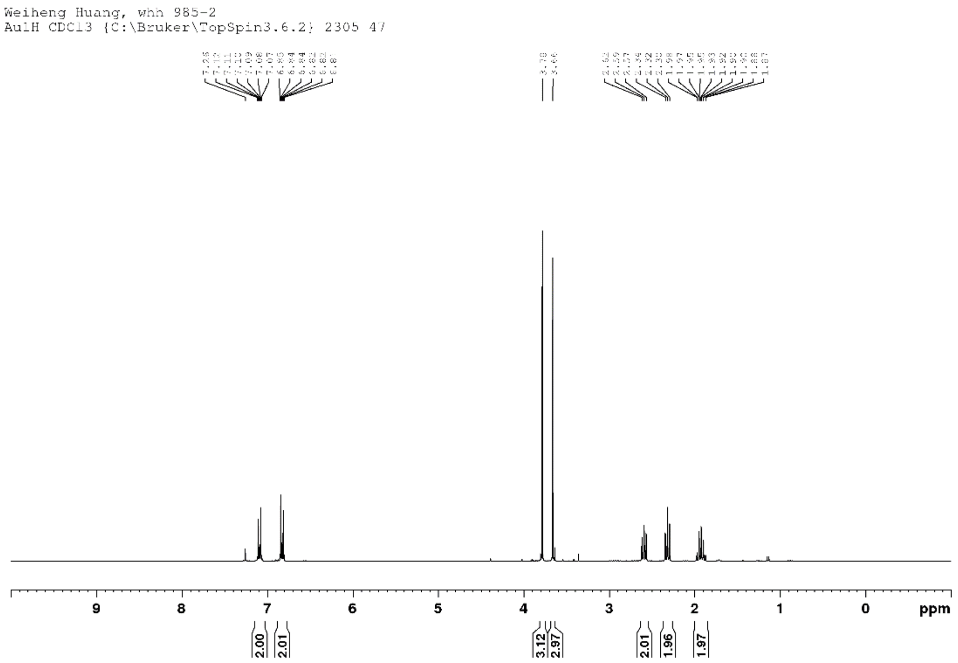

13C NMR (75 MHz, $CDCl_3$):

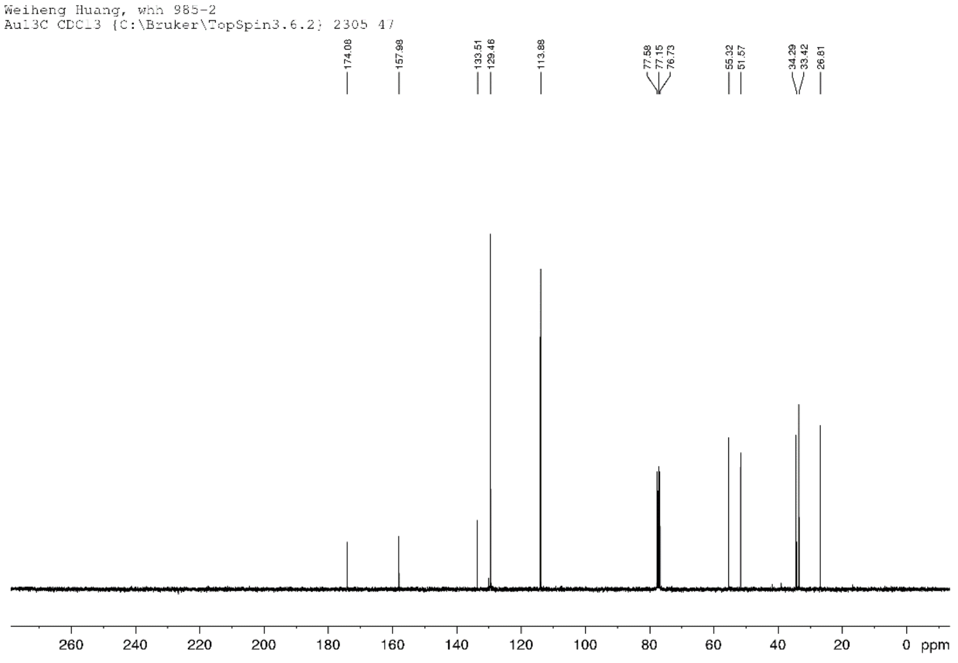

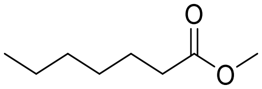

Methyl heptanoate (**2o**)
1H NMR (300 MHz, $CDCl_3$):

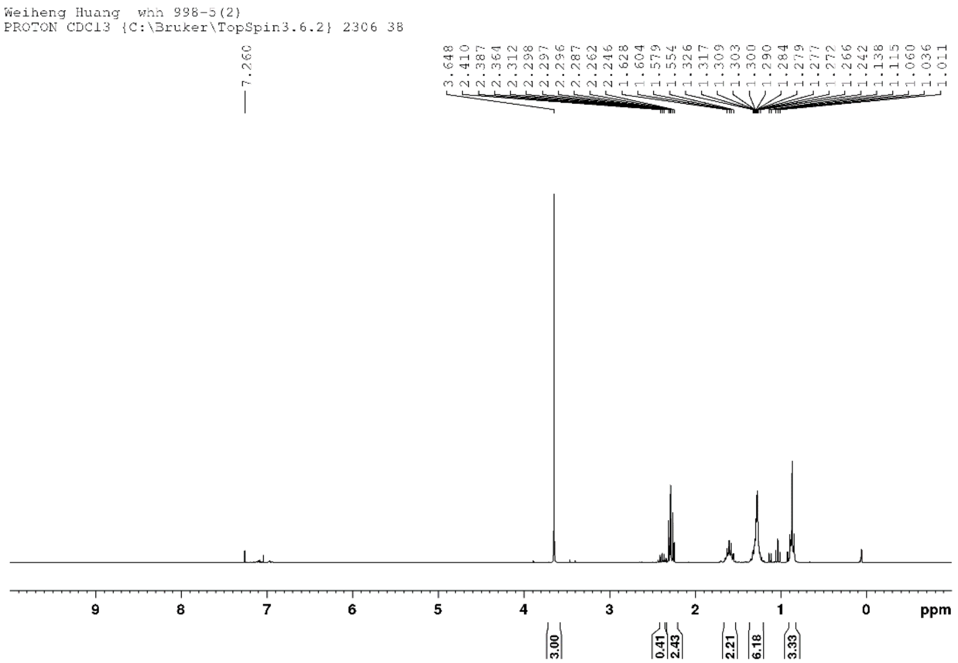

13C NMR (75 MHz, $CDCl_3$):

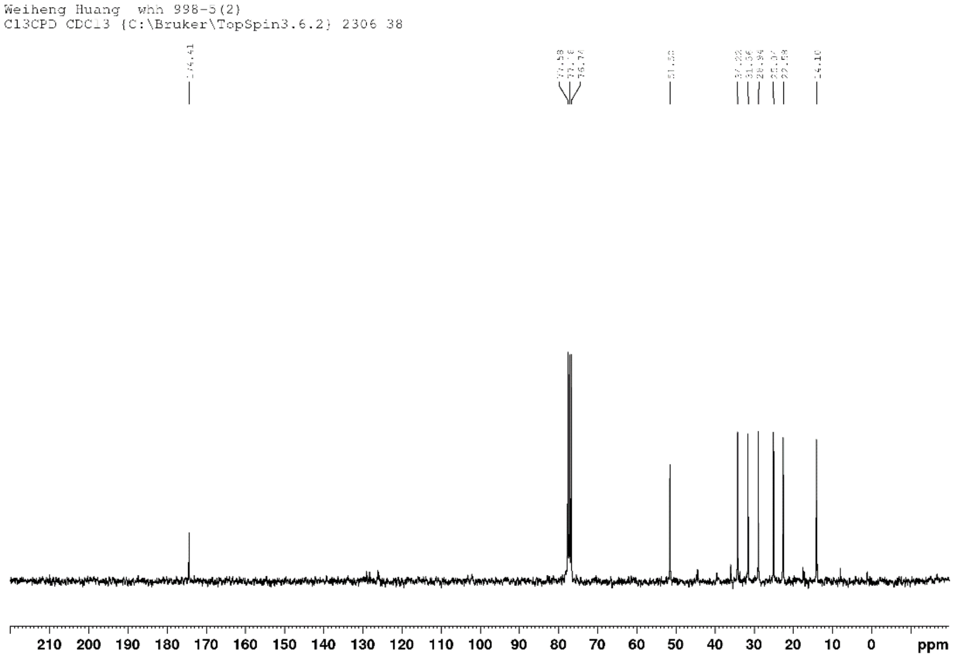

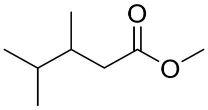

Methyl 3,4-dimethylpentanoate (**2q**)

1H NMR (300 MHz, $CDCl_3$):

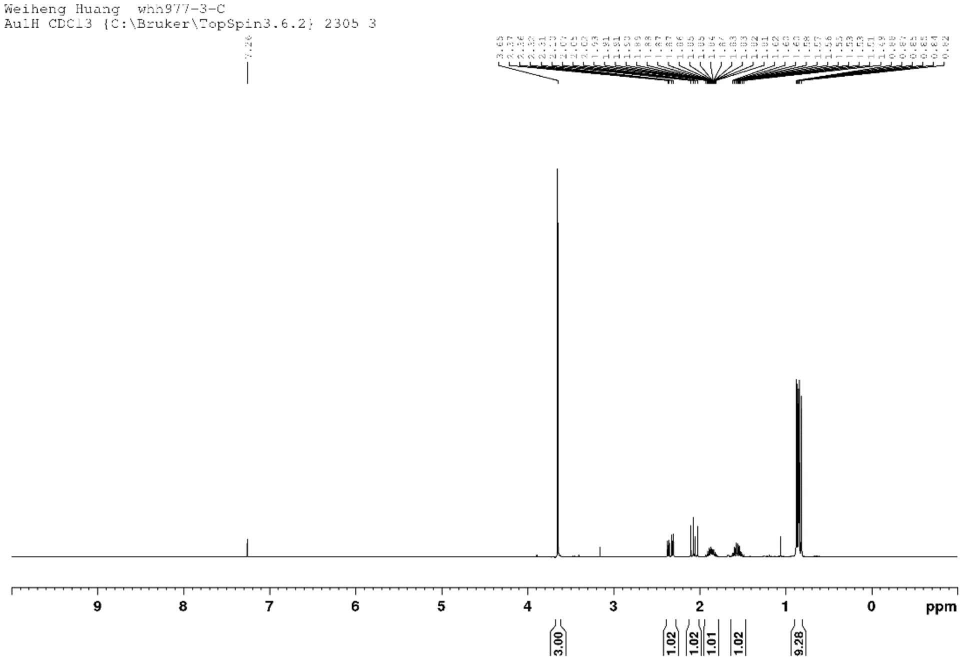

13C NMR (75 MHz, $CDCl_3$):

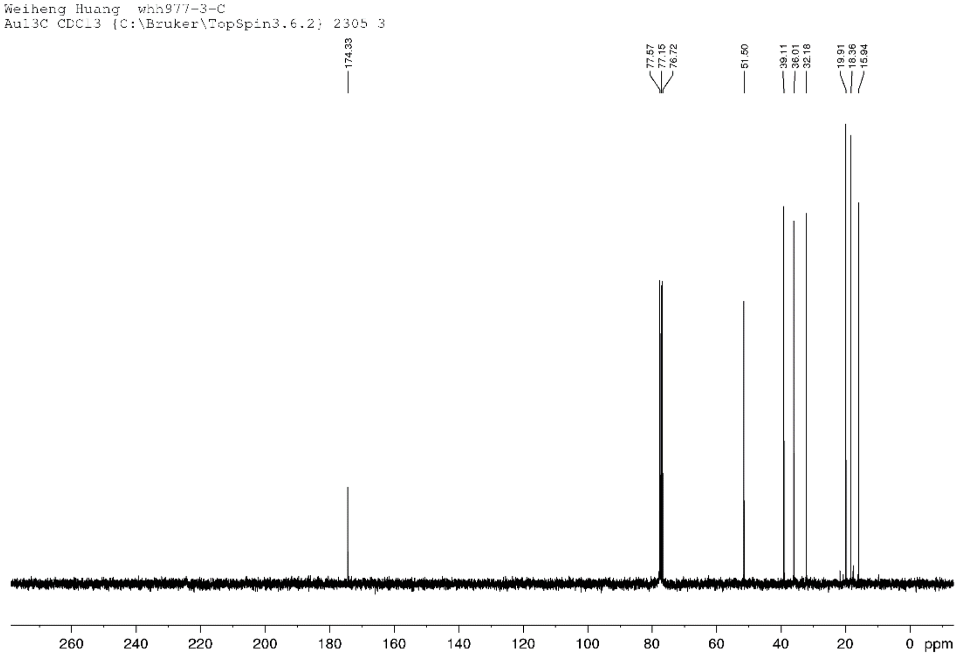

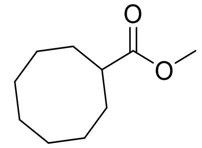

Methyl cyclooctanecarboxylate (**2r**)
1H NMR (300 MHz, $CDCl_3$):

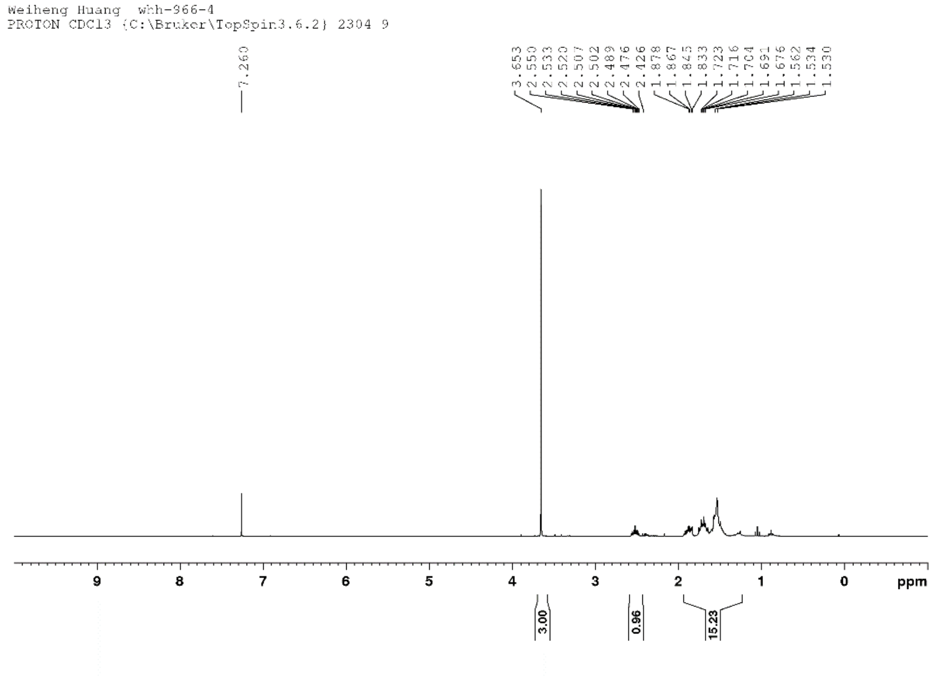

13C NMR (75 MHz, $CDCl_3$):

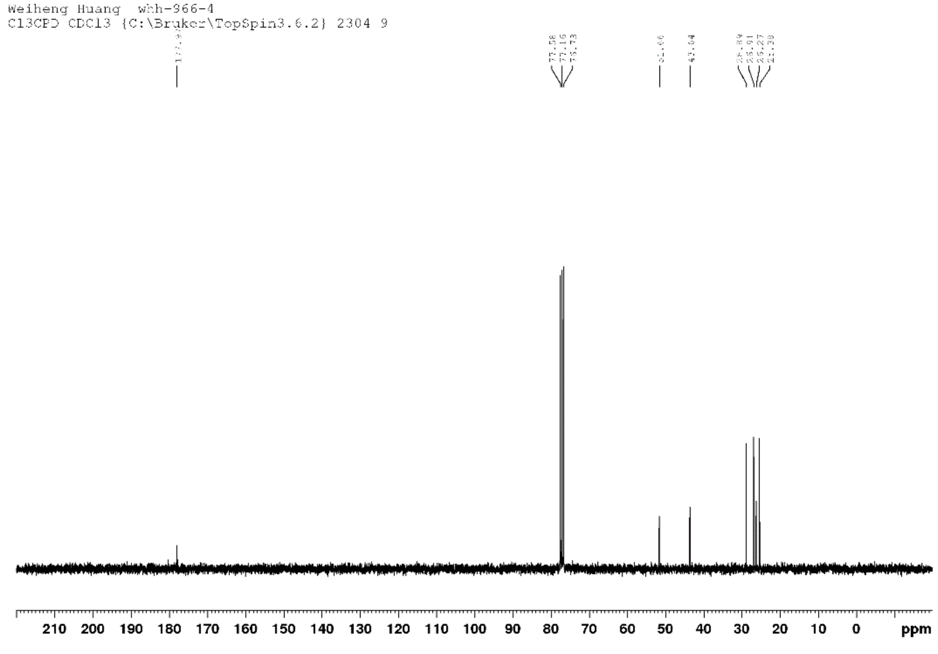

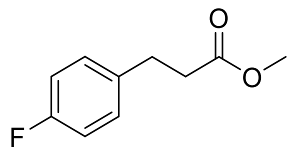

Methyl 3-(4-fluorophenyl)propanoate (**2s**)

1H NMR (300 MHz, $CDCl_3$):

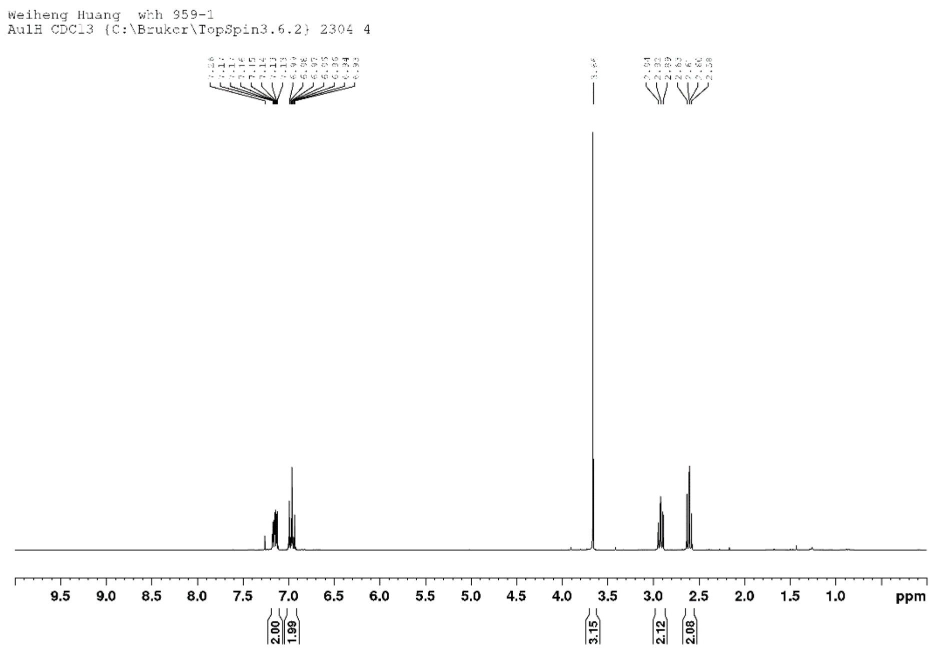

13C NMR (75 MHz, $CDCl_3$):

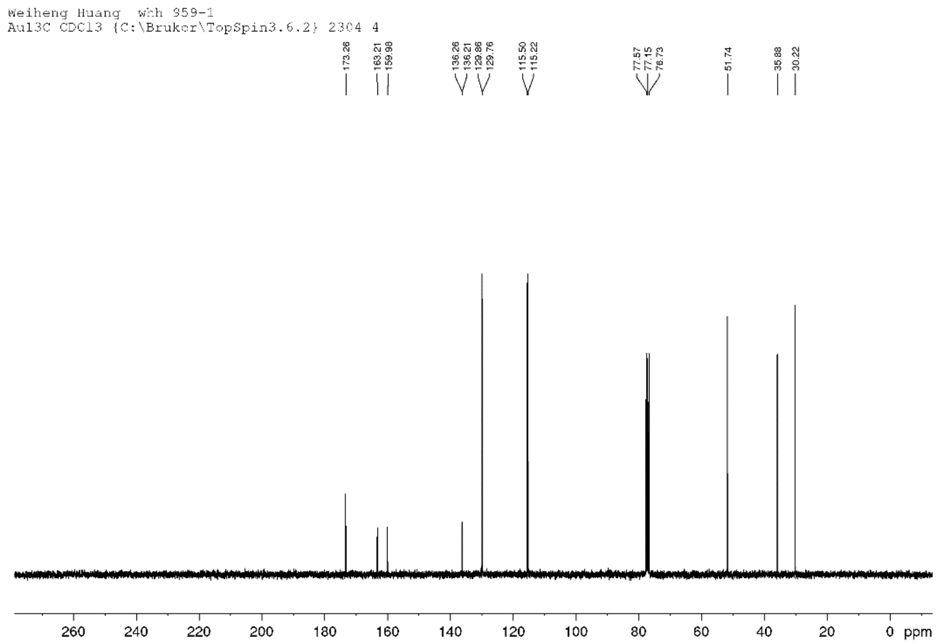

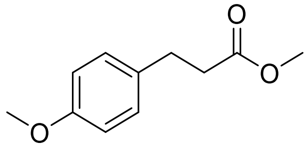

Methyl 3-(4-methoxyphenyl)propanoate (**2t**)

1H NMR (300 MHz, $CDCl_3$):

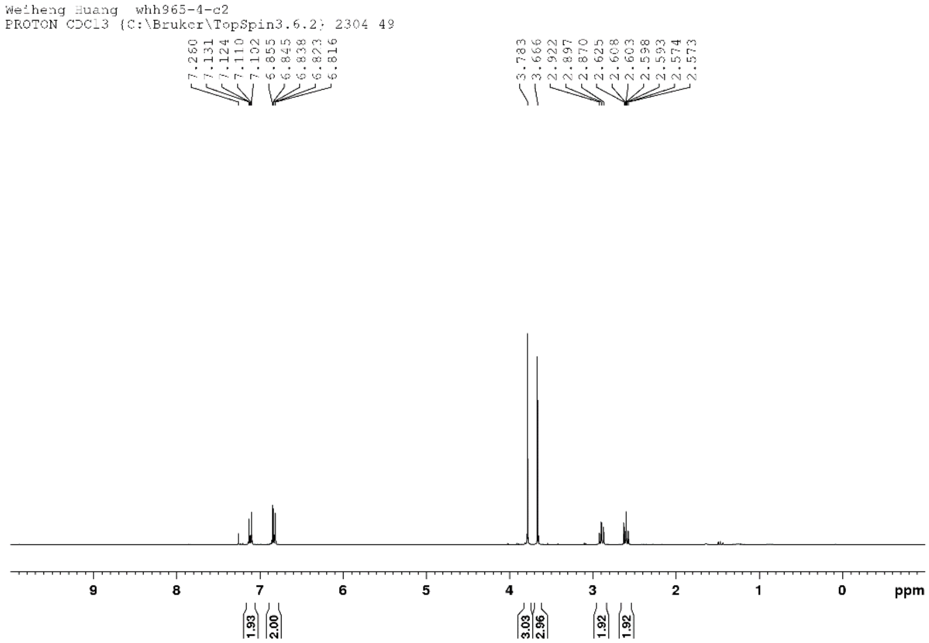

13C NMR (75 MHz, $CDCl_3$):

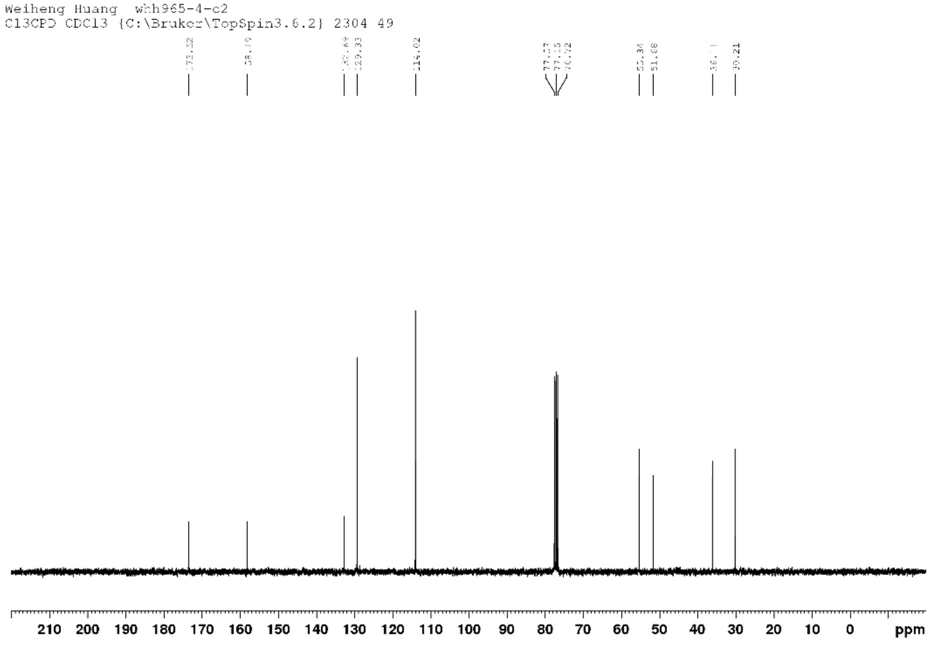

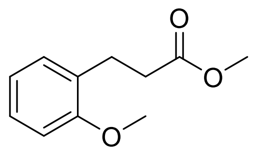

Methyl 3-(2-methoxyphenyl)propanoate (**2u**)

1H NMR (300 MHz, $CDCl_3$):

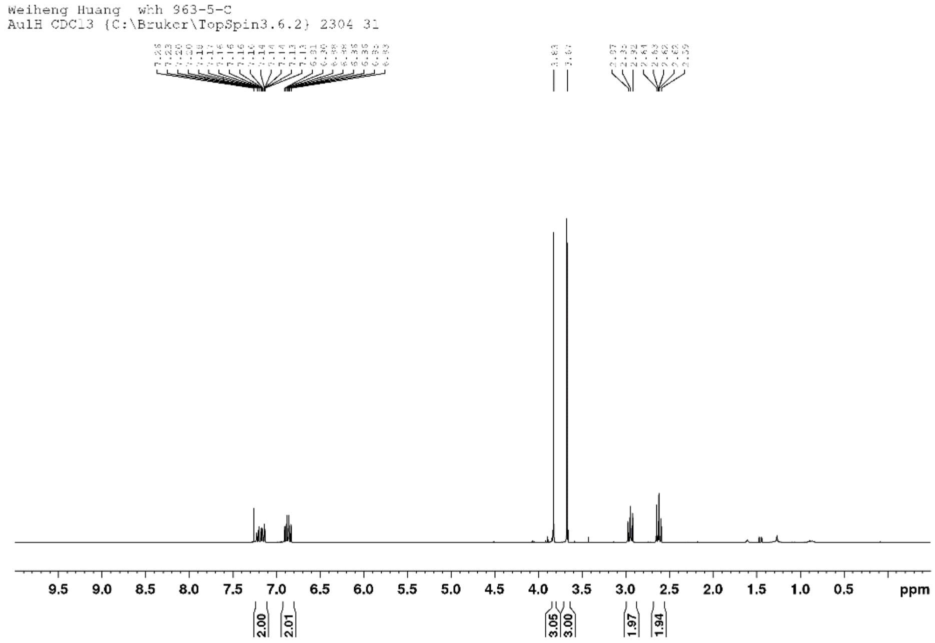

13C NMR (75 MHz, $CDCl_3$):

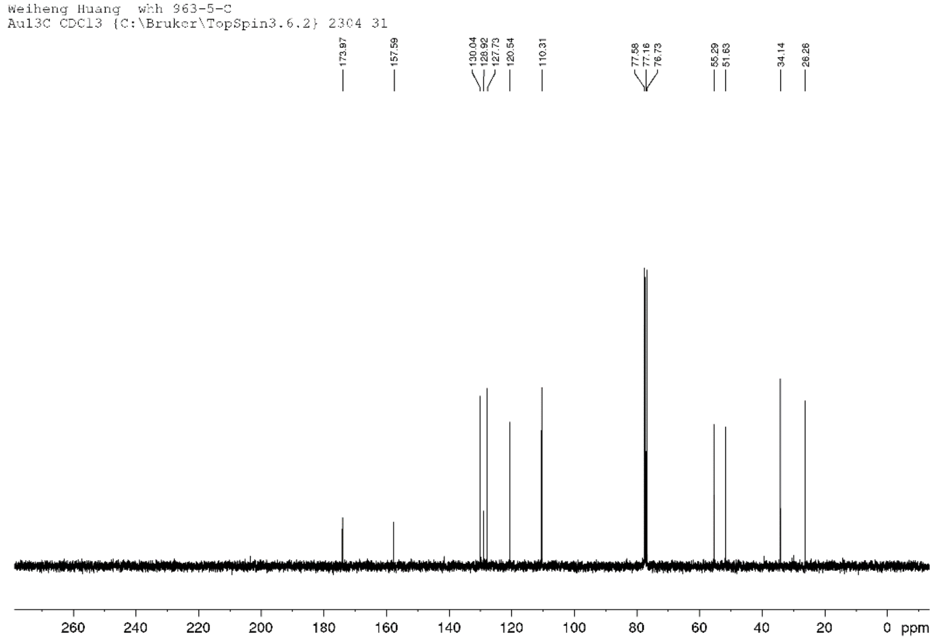

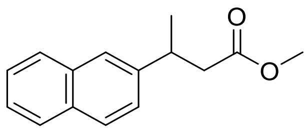

Methyl 3-(naphthalen-2-yl)butanoate (**2v**)
1H NMR (300 MHz, $CDCl_3$):

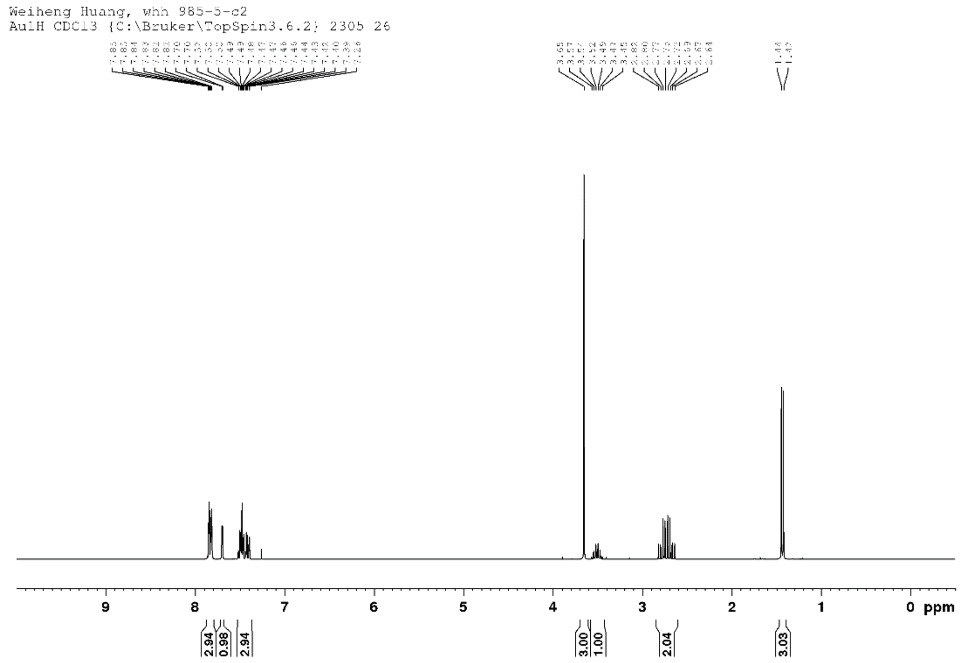

13C NMR (75 MHz, $CDCl_3$):

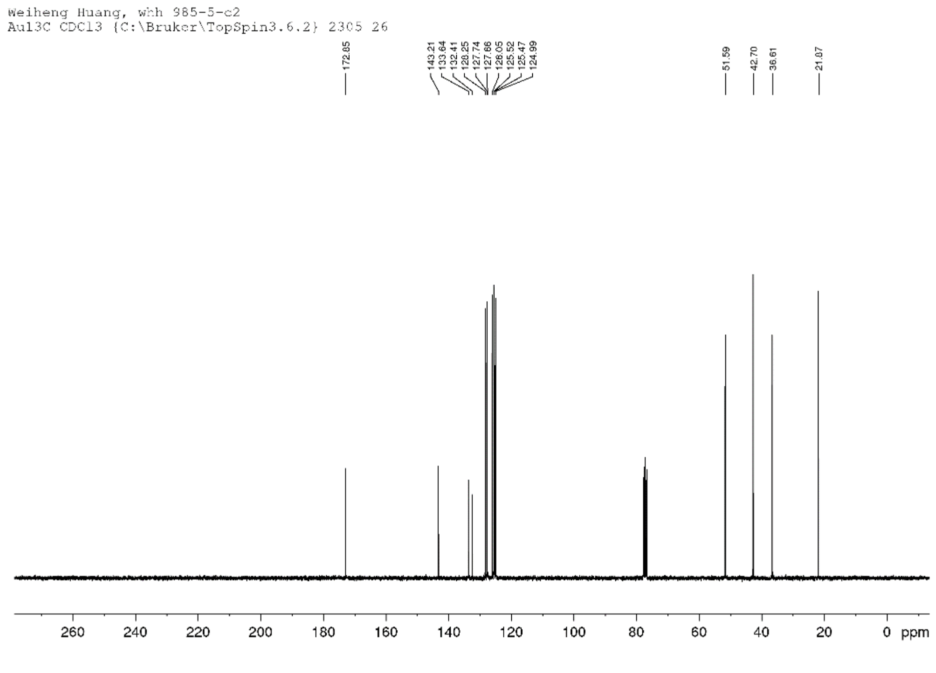

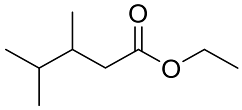

Ethyl 3,4-dimethylpentanoate (**2w**)

1H NMR (300 MHz, $CDCl_3$):

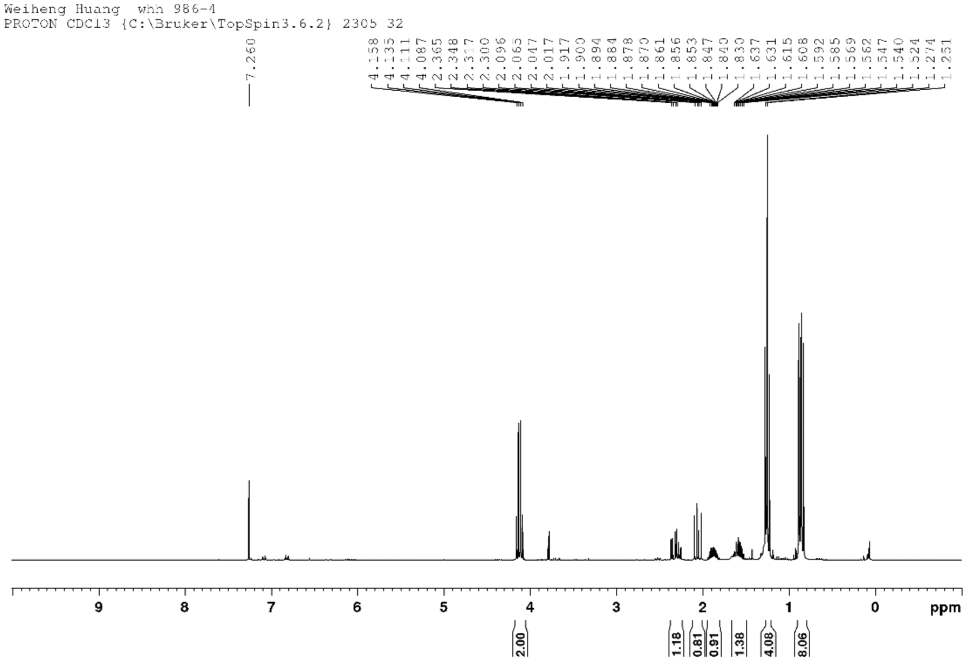

13C NMR (75 MHz, $CDCl_3$):

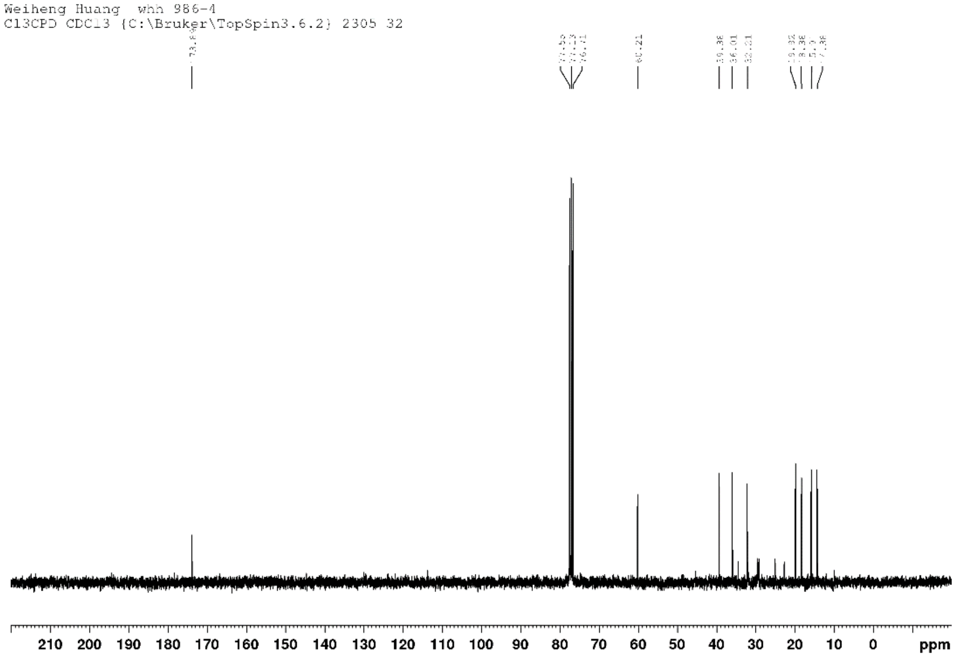

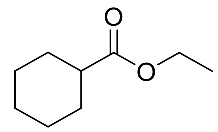

Ethyl cyclohexanecarboxylate (**2x**)

1H NMR (300 MHz, $CDCl_3$):

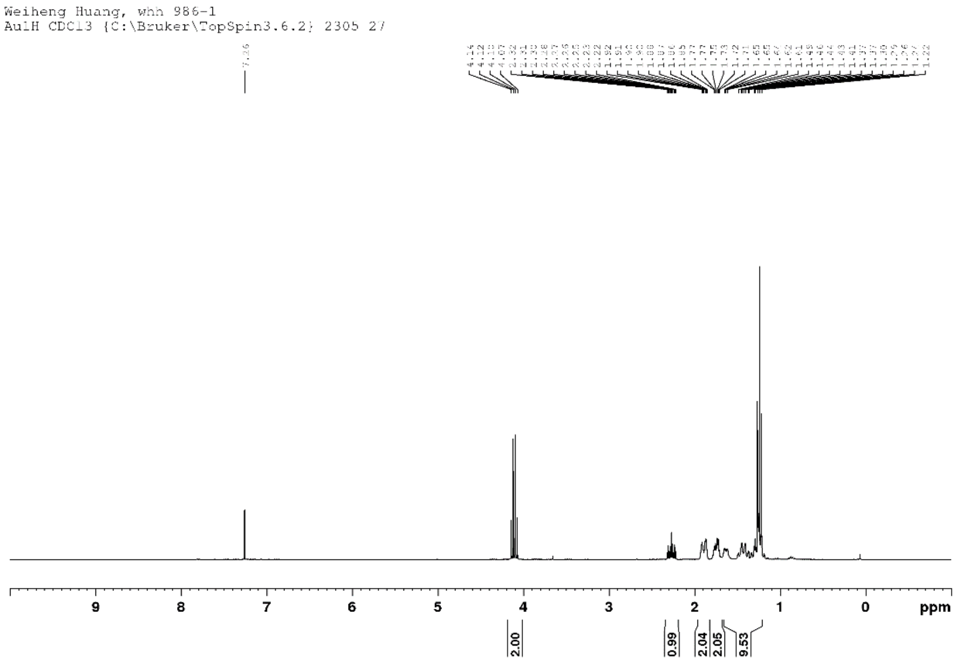

13C NMR (75 MHz, $CDCl_3$):

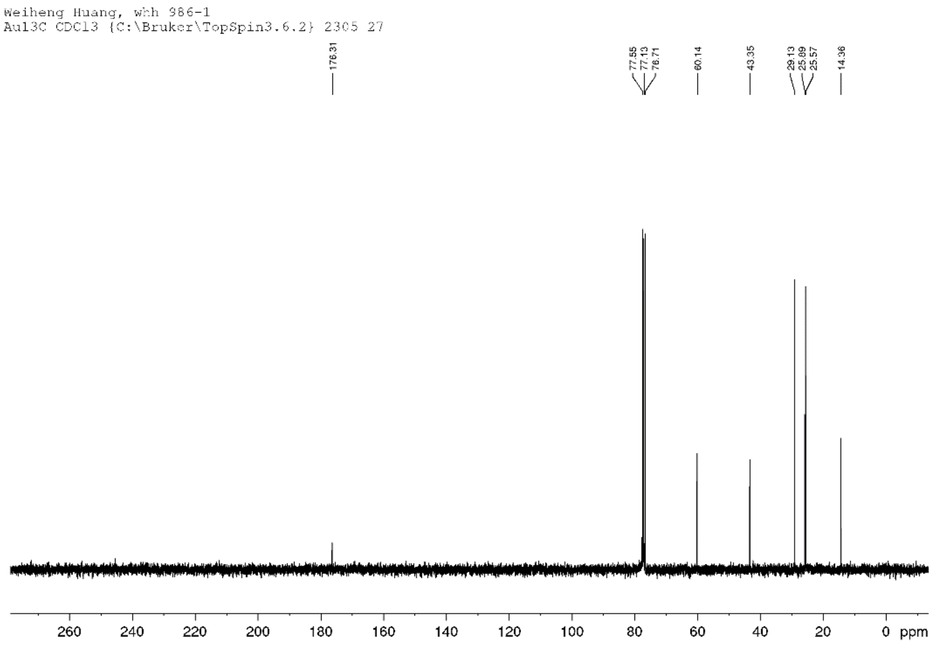

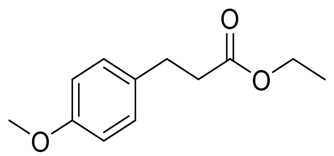

Ethyl 3-(4-methoxyphenyl)propanoate (**2y**)
1H NMR (300 MHz, $CDCl_3$):

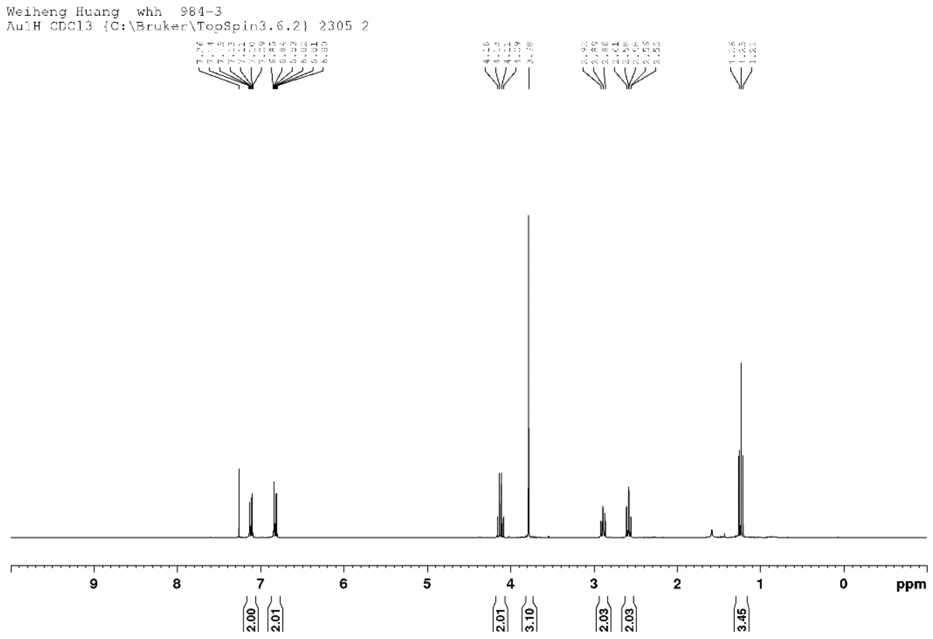

13C NMR (75 MHz, $CDCl_3$):

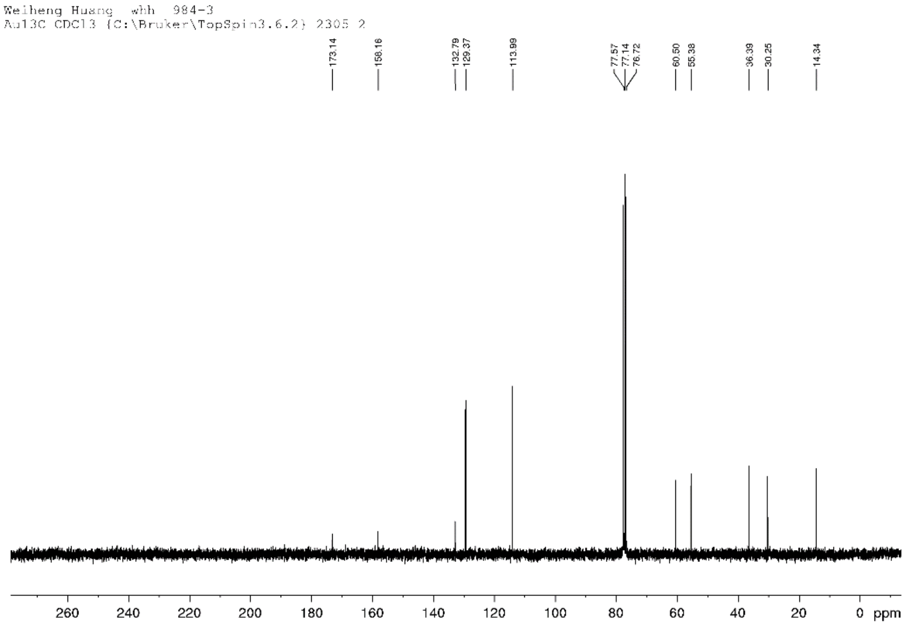

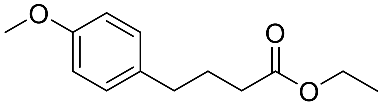

Ethyl 4-(4-methoxyphenyl)butanoate (**2z**)

1H NMR (300 MHz, $CDCl_3$):

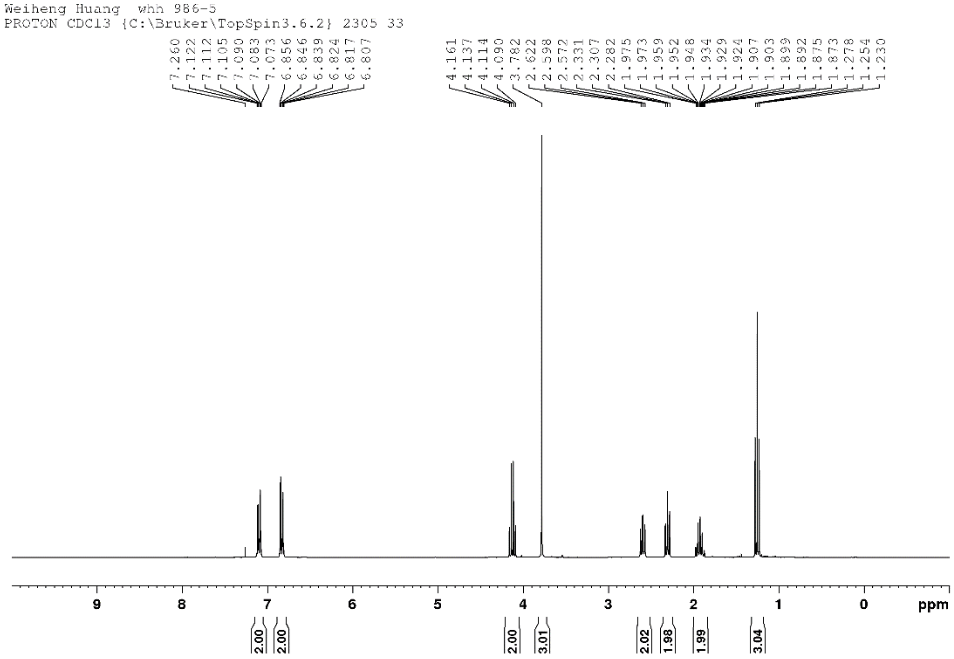

13C NMR (75 MHz, $CDCl_3$):

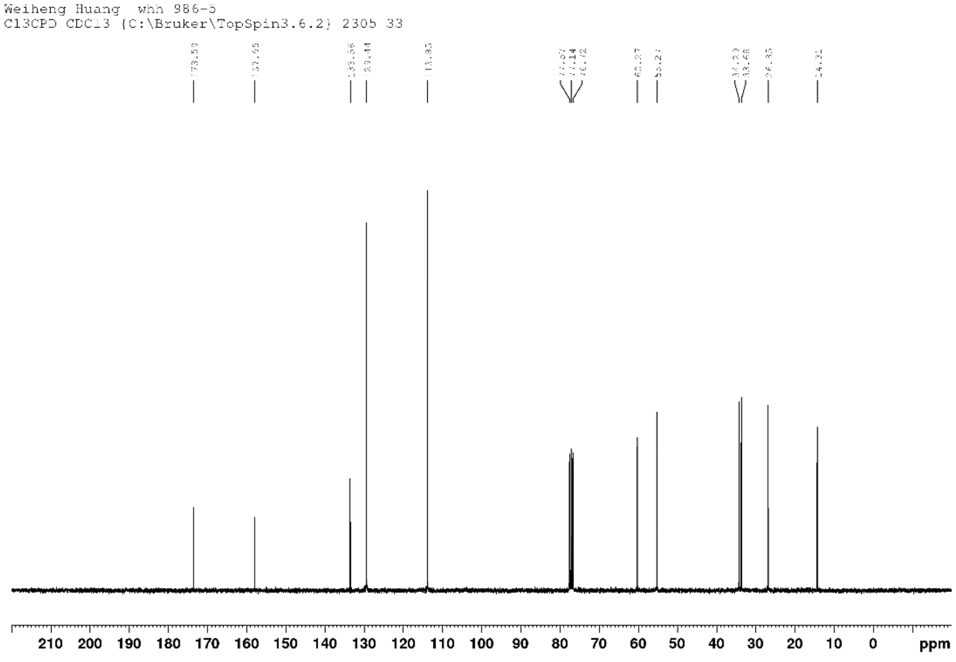

10. Supplementary References

28. Frisch, M. J. et al. Gaussian 16, Revision A.03 (Gaussian, Inc., 2016).
29. Zhao, Y., & Truhlar, D. G. A new local density functional for main-group thermochemistry, transition metal bonding, thermochemical kinetics, and noncovalent interactions. *J. Chem. Phys.* **125**, 194101 (2006).
30. Hay, P. J., & Wadt, W. R. Ab initio effective core potentials for molecular calculations. Potentials for K to Au including the outermost core orbitals. *J. Chem. Phys.* **82**, 299-310 (1985).
31. Schäfer, A., Huber, C., & Ahlrichs, R. Fully optimized contracted Gaussian basis sets of triple zeta valence quality for atoms Li to Kr. *J. Chem. Phys.* **100**, 5829-5835 (1994).
32. Marenich, A. V., Cramer, C. J., & Truhlar, D. G. Universal solvation model based on solute electron density and on a continuum model of the solvent defined by the bulk dielectric constant and atomic surface tensions. *J. Phys. Chem. B* **113**, 6378-6396 (2009).
33. Purification of Laboratory Chemical (Eds: Perrin, D. D.; Armarego, W. L. F.-). Pergamon Press, Oxford, **1988**, *3*.
34. J. D. Nobbs, C. H. Low, L. P. Stubbs, C. Wang, E. Drent, M. van Meurs, Organometallics, **2017**, *36*, 391-398.
35. P. Renaud, C. Ollivier, V. Weber, *J. Org. Chem.*, **2003**, 68, 5769-5772; F. Blank, H. Scherer, C. Janiak, *J. Mol. Catal. A Chem.*, **2010**, 330, 1-9; M. Nakazaki, K. Naemura, S. Nakahara, *J. Org. Chem.*, **1978**, 43, 4745-4750.
